# Antioxidant activity, molecular docking, and modeling pharmacokinetics study of some benzo[*f*]quinoline candidates

**DOI:** 10.1038/s41598-025-99811-1

**Published:** 2025-05-13

**Authors:** Sara F. El-Fagal, Eman A. E. El-Helw, Eman A. El-Bordany, Eman A. Ghareeb

**Affiliations:** Chemistry Department, Faculty of Science, Ain Shams University, Cairo, 11566 Egypt

**Keywords:** Antioxidant, Benzo[*f*]quinolone, Benzoxazinone, In silico, Docking, Chemical biology, Chemistry

## Abstract

Benzoquinolines were found in many pharmaceuticals and natural products and were utilized as templates for the synthesis of many drugs. Thus, 3-(3-chlorobenzo[*f*]quinolin-2-yl)-2-(4-oxo-4*H*-benzo[*d*][1,3]oxazin-2-yl)acrylonitrile was prepared as a key building substrate, using arylidene ethyl cyanoacetate derivative **3**, and reacted with diverse mono- and bi-dentate nitrogen nucleophiles aiming to construct new heterocycles based on a benzo[*f*]quinoline core, for example quinazolinone, imidazoline, oxadiazolinone, and benzimidazole derivatives. The antioxidant activity of the synthesized compounds was evaluated, using ascorbic acid as a reference, and revealed the highest potency of benzimidazole derivative **19**, which may be attributed to the aromaticity and extended conjugation. These findings were supported by in silico studies. A molecular docking simulation was performed to disclose the modes of interactions of benzimidazole **19** toward HCV NS5B polymerase. It exhibited a binding energy greater than that of co-crystallized ligand, referring to strong binding to certain key nucleobases and amino acids (CYS 366 and ASN 411) of HCV NS5B polymerase through hydrogen bonding and pi-hydrogen interactions, revealing its potential usage as an antioxidant agent. DFT simulation for the active compounds were studied to determine the molecular geometry and frontier orbitals of the potent compounds. Regarding ADME simulation, compounds** 3**,** 9**, and **17** exhibited a high GI absorption and good bioavailability score of 0.85, 0.55, and 0.55, respectively. The variance in GI absorption might depict the differences in observed antioxidant efficacy of compounds. Also, they showed gastrointestinal tract (GIT) absorption due to their being in the BOILED-EGG chart white area. The potent compounds **3, 9, 13, 17,** and **19** exhibited fair TPSA and predicted to exhibit good passive oral absorption.

## Introduction

Benzoquinoline and its derivatives play a crucial role in pharmaceutical chemistry, and they are considered as a precursor for the construction of heterocyclic compounds that possess a wide spectrum of biological activities including antioxidant^1,2^, antibacterial, antifungal, anti-inflammatory, anticancer, anti-Alzheimer’s, and insecticidal properties^3–13^. Quinoline and its candidates are extensively employed as devoted compounds to prepare a diversity of heterocycles with a broad array of pharmacological actions^14–17^. Also, benzoquinolines were found in many pharmaceuticals and natural products and were utilized as templates for the synthesis of many drugs^4,8^. These compounds might act as an antioxidant by reacting with various free radicals through hydrogen atom or single electron transfer. Oxidative stress is one the primary contributors to significant severe diseases, which arises from the over-production of reactive oxygen species (ROS) in the human body.

ROS describes several reactive molecules and free radicals derived from molecular oxygen such as hydrogen peroxide (H_2_O_2_), hydroxyl radical (^•^OH), peroxyl radical (ROO^•^), and superoxide anion radical (O_2_^•−^). The high levels of such species can damage biological molecules such as proteins, carbohydrates, lipids, and DNA, leading ultimately to cellular dysfunction. Therefore, there is a significant demand for the development of new potent antioxidants that can delay or prevent oxidative damage in the human body via scavenging or regulating the formation and elimination of ROS.

Otherwise, benzoxazinone derivatives are an essential class of benzo-fused heterocycles due to their facile ring opening with nitrogen nucleophiles producing acyclic and heterocyclic candidates with diverse biological activity^18–23^. In continuation to our program for the synthesis of various biologically active candidates^24–28^, these findings directed our attention to a design and synthesize new heterocyclic system that incorporated both benzoquinoline and benzoxazine frameworks and studied its reactivity towards some mono- and bi-dentate nitrogen nucleophiles aiming to obtain new heterocycles with potential antioxidant activity (to protect cellular components from free radical damage) and supported by in silico studies.

### Rationale and design

**Fig. 1:**
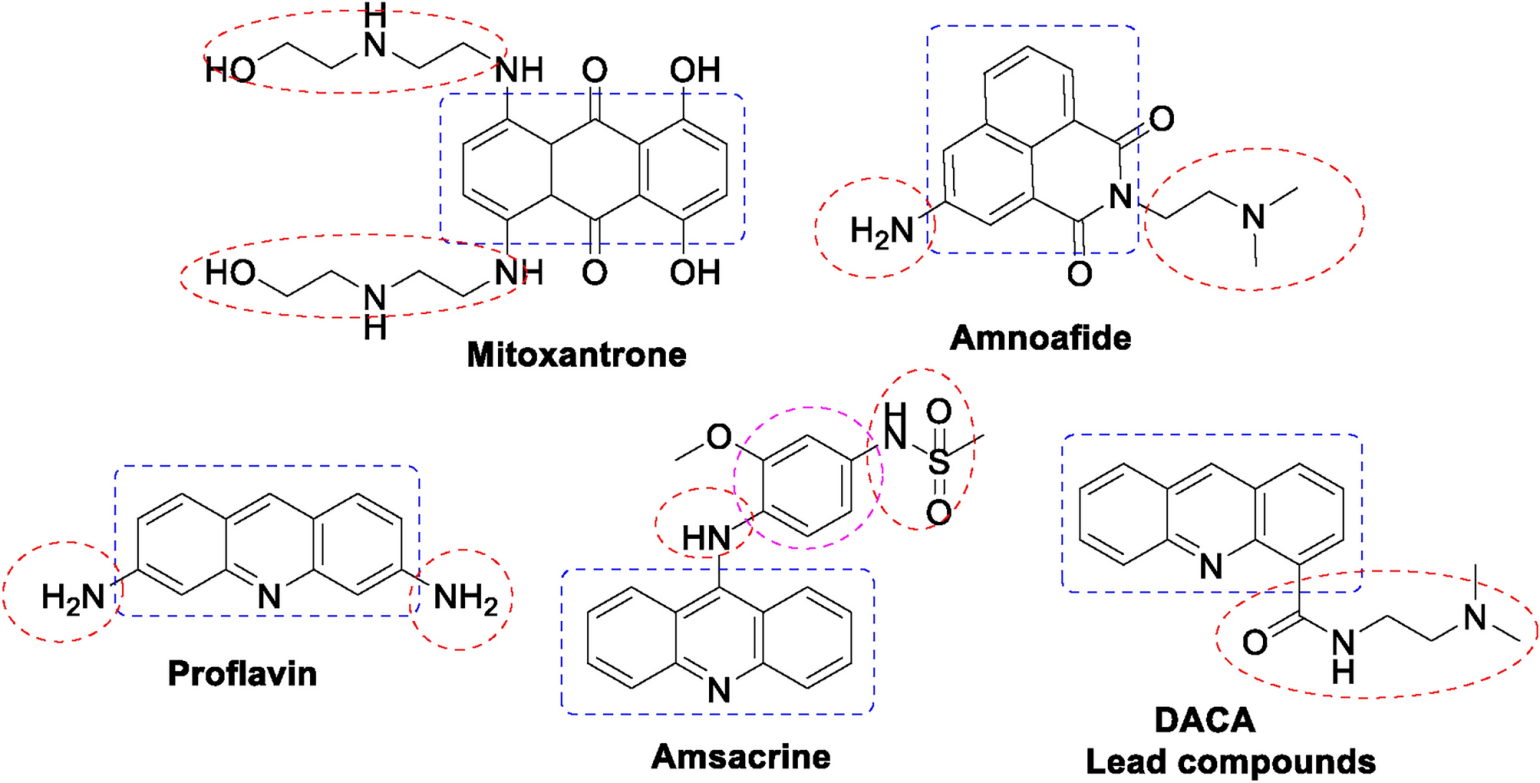
Some biologically active agents.

One of the most important targets for the design of new antioxidant drugs is polyaromatic ligands of larger surface area and applicable steric properties^1^. Some of these planar polyaromatic candidates were biologically active agents, which were shown in Fig. 1. The nature, tautomerism, and size of chromophores are important parameters that govern the radical scavenging activity^26,28^. Recently, much consideration has been promoted to design and synthesize novel and efficient targeted antioxidant agents encompassing benzo[*f*]quinoline scaffold^29,30^.

**Fig. 2:**
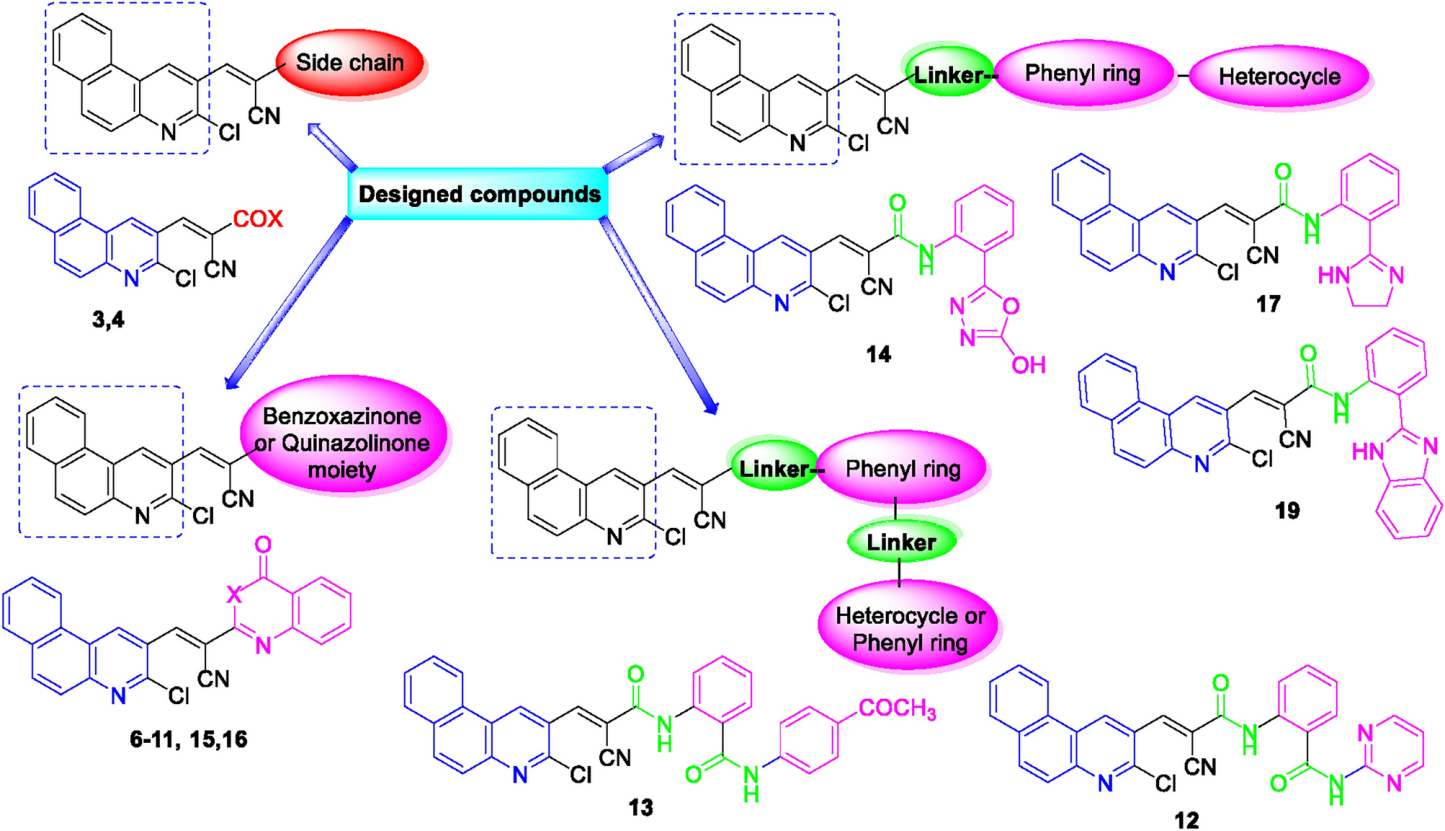
Our designed substrates.

The candidates with proper functional substituents (e.g., OH or NH) can exhibit great efficacy in antioxidant activity due to their potent abilities to scavenge ROS directly through hydrogen or electron transfer. Thus, this work reports the molecular design and synthesis of a series of new benzo[*f*]quinolines (planar polyaromatic ligands) encompassing either side chain (hydrophilic and hydrophobic groups as compounds **3** and **4**), phenyl, or heterocyclic scaffolds (enhancing lipophilic interactions) at position-3 of quinoline core. The rationale for the design of these substrates can be shown in Fig. 2 starting from a benzoquinoline scaffold through an insertion of side chains and heterocycles (like benzoxazinone, quinazolinone, pyrimidine, oxadiazole, imidazole, and benzimidazole) with or without linkers (amide groups). Compound **19**, a benzo[*f*]quinoline bearing a benzimidazole scaffold, displayed the best antioxidant properties among the synthesized candidates, through possible tautomerism and extended conjugation increasing its scavenging activity via increasing hydrogen bonding interactions and enhancing the lipophilicity properties.

## Results and discussion

### Chemistry

Owing to their diverse biological activities, our research focused on developing a framework of benzoxazinone skeleton based on a benzo[*f*]quinoline core. Thus, 3-chlorobenzo[*f*]-quinoline-2-carbaldehyde **(1)**^31^ condensed with ethyl cyanoacetate to afford the arylidene derivative **2** which was easily transformed into the corresponding acid **3** via hydrolysis using alcoholic NaOH followed by an acidification with dilute hydrochloric acid (10%). The IR spectrum of acid **3** lacked the ester carbonyl group and exhibited absorption bands for OH at ν 3440 cm^-1^ and acid C = O at ν 1706 cm^-1^. Treating the acid **3** with thionyl chloride yielded the corresponding acid chloride **4**. The IR spectrum of acid chloride **4** revealed that the absence of acid carbonyl and OH groups and the presence of an absorption band for C = O of acid chloride group at ν 1761 cm^−1^.

**Fig. 3:**
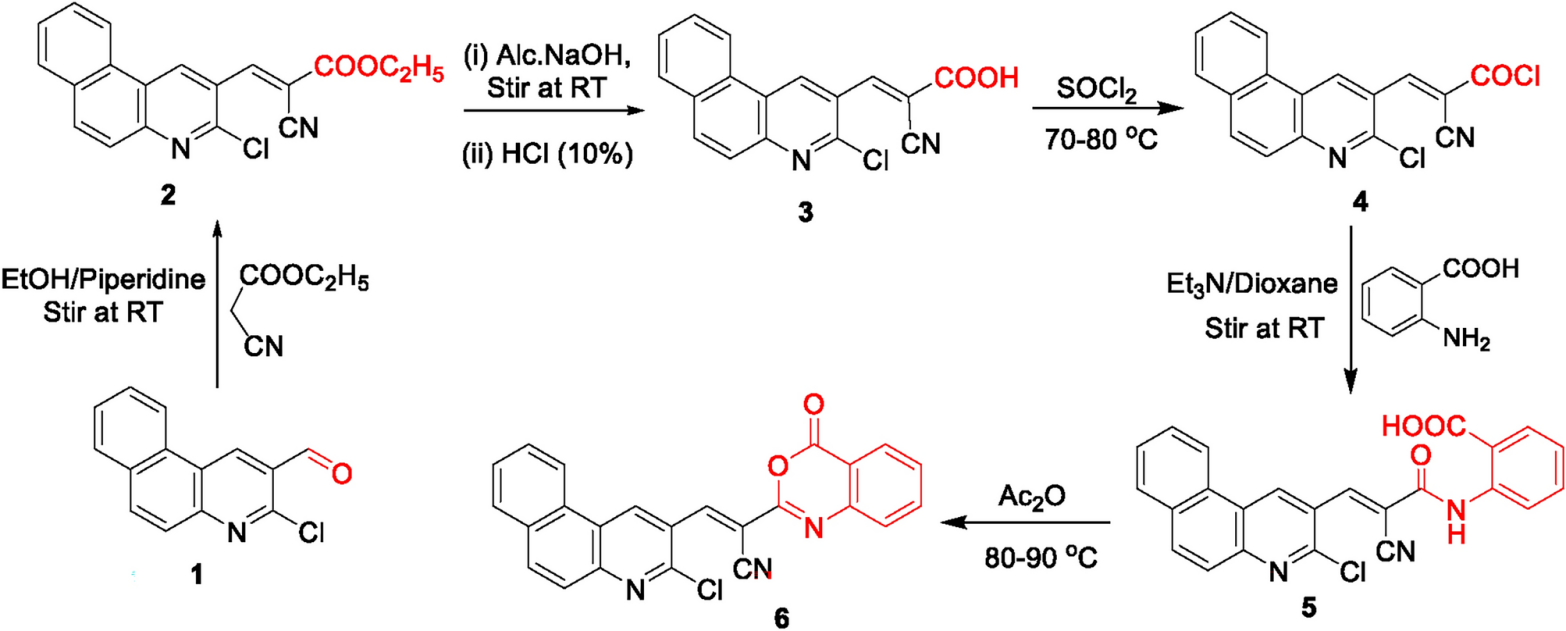
Synthesis of benzoxazinone derivative 6.

The benzoic acid derivative **5** was obtained from the treatment of the acid chloride derivative **4** with anthranilic acid in dry dioxane and triethyl amine (Et_3_N) at room temperature. The IR spectrum of compound **5** exhibited an absorption band for acid C = O at ν 1695 cm^-1^ and amide C = O at ν 1667 cm^-1^. Its ^1^H NMR spectrum exhibited an exchangeable OH proton in the downfield region δ 12.85 ppm, in addition to a singlet signal for the NH proton at δ 9.00 ppm. Cyclization of the benzoic acid derivative in the presence of acetic anhydride furnished the benzoxazinone derivative **6** (Fig. 3). The IR spectrum of benzoxazinone **6** showed an absorption band for lactone C = O at ν 1774 cm^-1^. Another evidence for its structure was achieved from its ^1^H NMR spectrum which lacked the labile protons of OH and NH. The mass spectra of compounds **3–6** provided the correct molecular ion peaks.

**Fig. 4:**
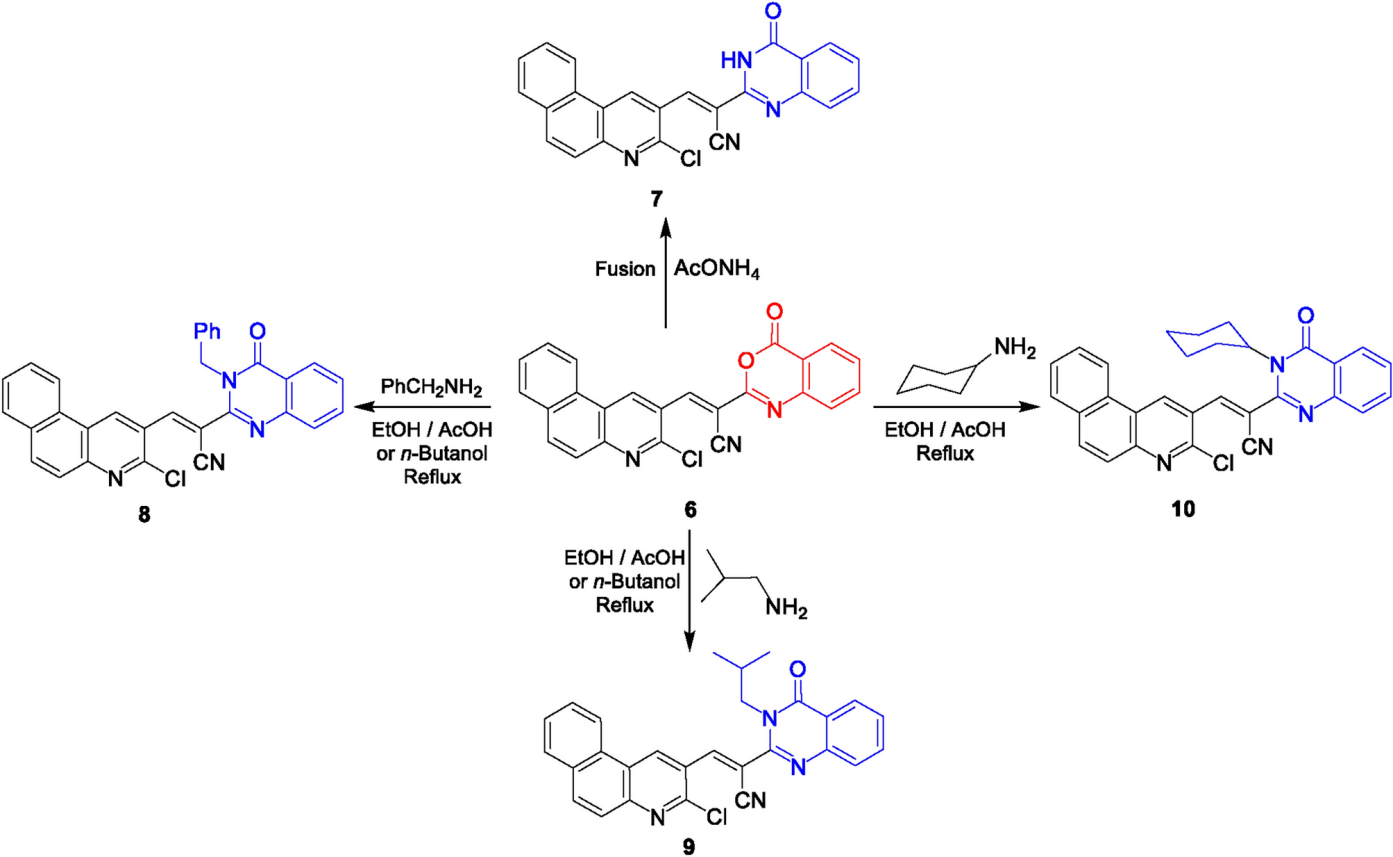
Reactions of benzoxazine **6** with ammonium acetate and some aliphatic amines.

The benzoxazinone **6** was submitted to react with some mono- and bi-nitrogen nucleophiles to construct other heterocyclic systems. Initially, the ammonolysis of benzoxazinone **6** with ammonium acetate under fusion condition for 6 h afforded the quinazoline derivative **7**. The IR spectrum lacked the lactone absorption band and the ^1^H NMR spectrum showed one proton of NH group at δ 10.42 ppm. Treating the benzoxazine derivative **6** with benzylamine, isobutyl amine or cyclohexylamine in refluxing *n*-butanol or ethanol/glacial acetic acid yielded the corresponding quinazoline derivatives **8–10** (Fig. 4).

The IR spectra of compounds **8–10** exposed the absence of the lactone C = O and the appearance of an absorption band for amide C = O. The ^1^H NMR of compound **8** revealed a singlet signal integrated into two protons at the up-field region δ 3.40 ppm corresponding to CH_2_ protons. The ^1^H NMR of compound **9** exposed a doublet signal for CH_2_ at δ 3.29 ppm integrated for two protons, multiplet signal for CH at δ 2.62 ppm, and doublet signal for two CH_3_ at δ 0.90 ppm integrated for six protons. The ^1^H NMR of compound **10** was quite consistent with the proposed structure.

**Fig. 5:**
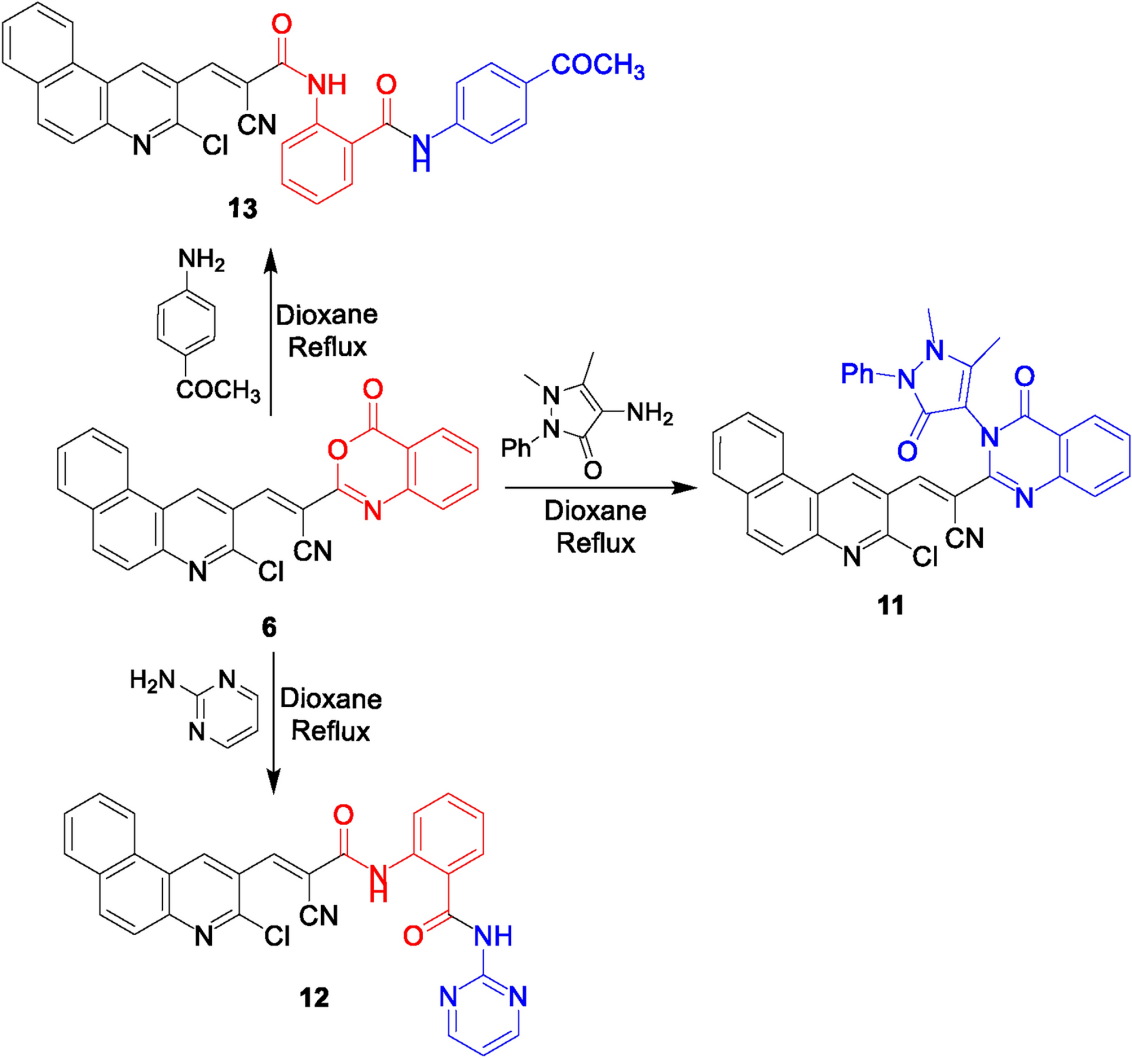
Reactions of benzoxazinone **6** with certain heterocyclic and aromatic amines.

Quinazoline bearing a pyrazolone ring **11** was achieved via the reaction of benzoxazinone derivative **6** with 4-amino-1,5-dimethyl-2-phenyl-1,2-dihydropyrazol-3-one in 1,4-dioxane. The IR spectrum lacked lactone carbonyl absorption and displayed the amide carbonyl absorption. Heating benzoxazinone **6** with aromatic amines as 2-aminopyrimidine or 4-aminoacetophenone furnished the benzamide derivatives **12** and **13**, respectively. Their IR spectra showed an absorption band for amide instead of lactone carbonyl absorption. The ^1^H NMR spectra of **12** disclosed two singlet signals at δ 12.69 and δ 9.02 ppm for 2 NH protons. While for compound **13**, it displayed two singlet peaks at δ 12.71 ppm and δ 8.90 ppm for 2 NH and one singlet peak integrated for three protons for CH_3_ group at δ 2.47 ppm which indicate the formation of compound **13** (Fig. 5).

**Fig. 6:**
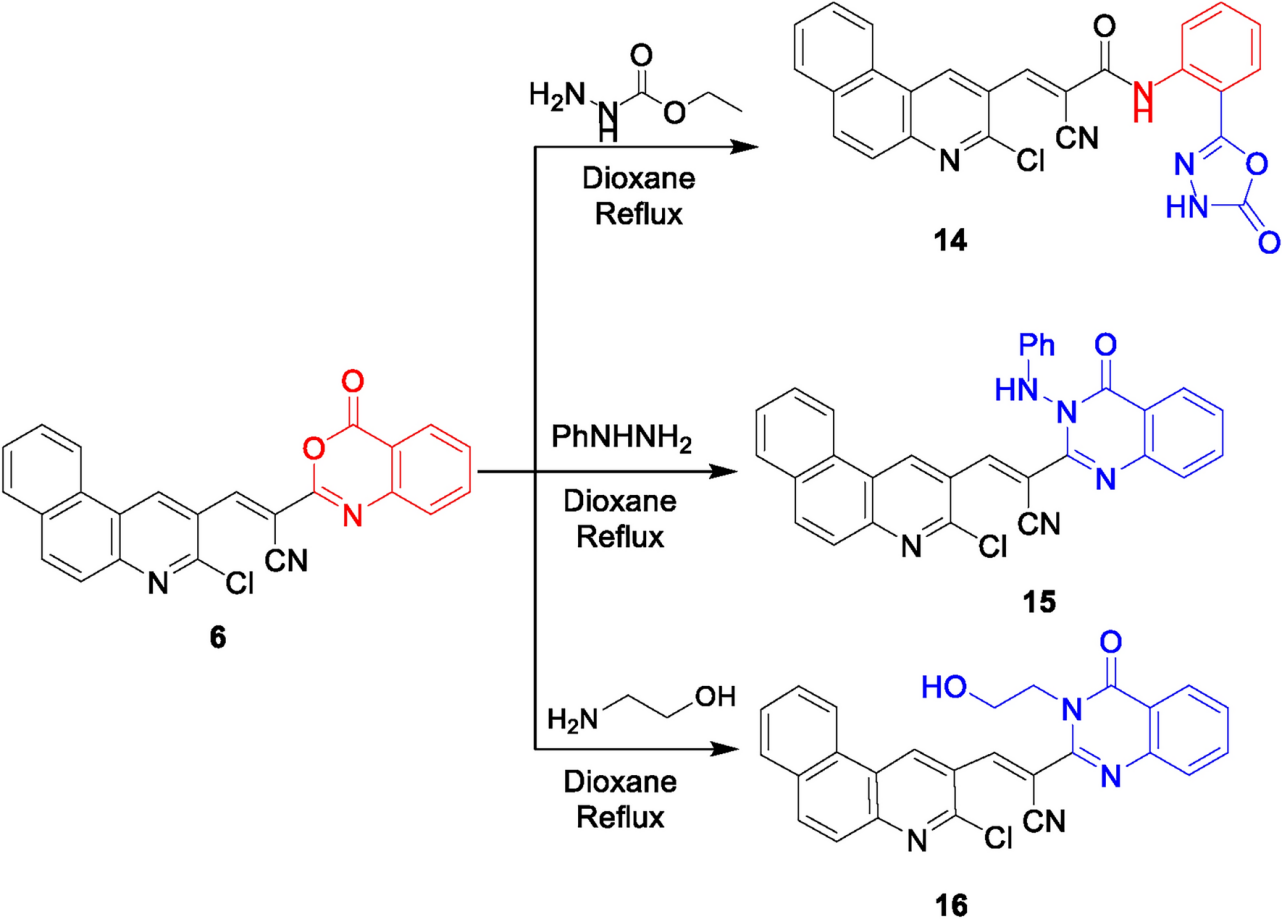
Reactions of benzoxazine derivative **6** with ethyl carbazate, phenylhydrazine, and ethanolamine.

**Fig. 7:**
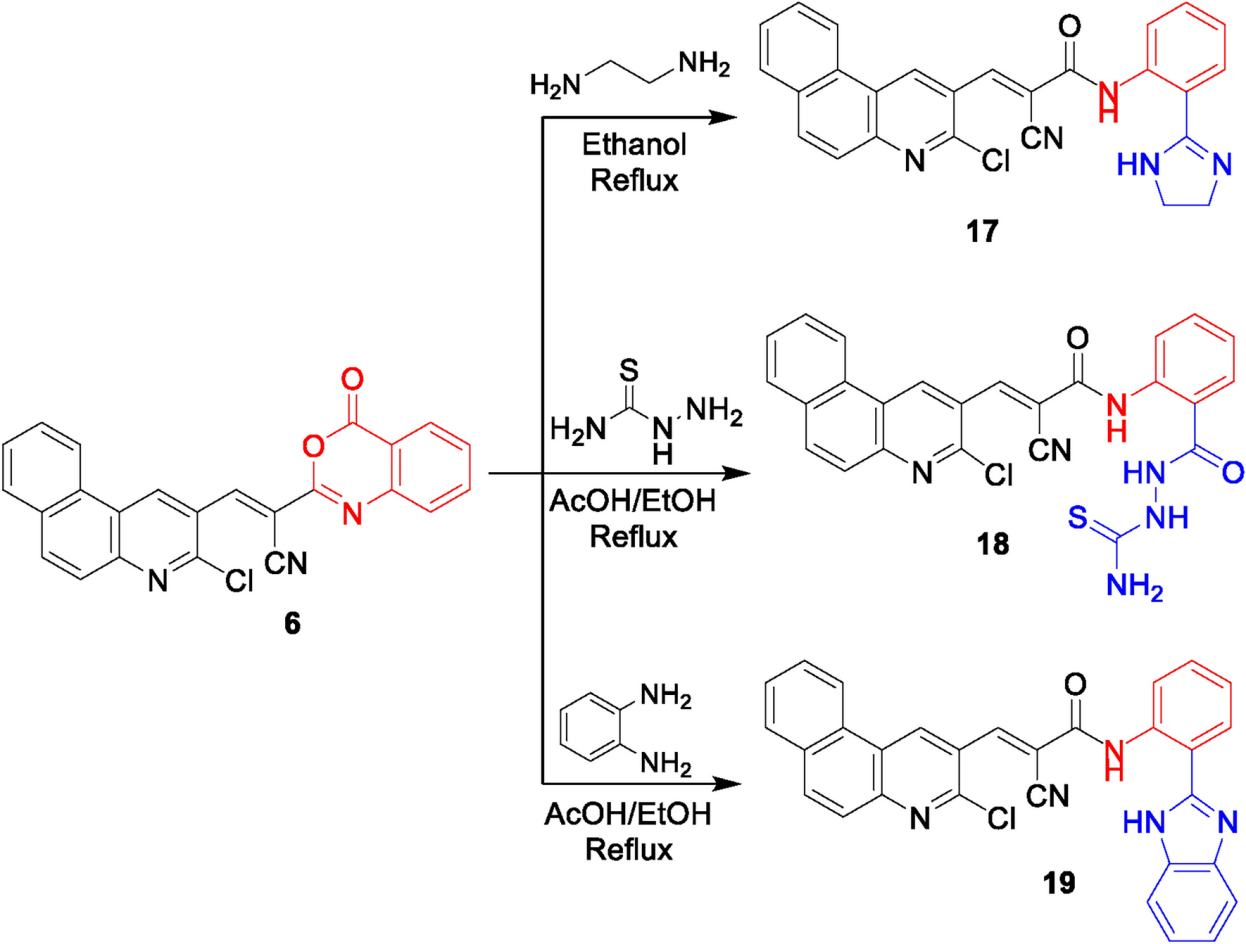
Reactions of benzoxazine with some 1,4-bidentate nucleophiles.

On the other hand, to investigate the behavior of benzoxazine derivative **6** towards different 1,2-and 1,4-bidentate nucleophiles, it was submitted to react with ethyl carbazate, phenylhydrazine, ethanolamine, ethylenediamine, thiosemicarbazide and 2-aminoaniline (Figs. 6, 7). Indeed, treating benzoxazinone **6** with ethyl hydrazine carboxylates or phenylhydrazine in refluxing dioxane afforded the 5-hydroxy-1,3,4-oxadiazole derivative **14** and the quinazoline derivative **15**, respectively. The IR spectra of compounds **14** and **15** lacked lactone carbonyl and showed an absorption band for C = O amide. The ^1^H NMR spectrum of compound **14** disclosed two broad singlet peaks at δ 13.04 and 8.92 ppm for OH and NH, respectively. Meanwhile, ^1^H NMR spectrum of compound **15** exposed a broad singlet signal in the downfield region at δ 13.38 ppm for NH. Conducting the benzoxazinone **6** with 1,4-binucleophile as 2-aminoethanol in dioxane yielded the quinazoline derivatives **16**. Its ^1^H NMR spectrum demonstrated a singlet signal at δ 3.73 ppm for OH and two triplet signals for CH_2_-CH_2_ groups at δ 3.02 and 2.61 ppm each integrated for 2 protons which confirmed the assigned structure (Fig. 6).

On the other hand, the imidazoline derivative **17** was obtained from the condensation of benzoxazine derivative **6** with ethylenediamine in ethanol (Fig. 7). Its IR spectrum lacked lactone carbonyl absorption, and displayed the amide carbonyl absorption, The ^1^H NMR displayed two singlet signals at δ 13.20 ppm and δ 8.97 ppm for 2 NH and two triplet signals at δ 3.99 ppm and δ 3.22 ppm for two CH_2_ groups. A plausible pathway for this reaction was represented in Fig. S1. Initially, a lactone ring opening of substrate **6** had occurred by nucleophilic attack of primary amino group of ethylenediamine followed by 5-exo-trig cyclization to remove a water molecule to form an imidazoline candidate **17**.

The reaction of benzoxazinone **6** with thiosemicarbazide in ethanol and acetic acid underwent lactone ring opening to obtain the thiosemicarbazide derivative **18**. The IR spectrum showed absorption bands for NH, NH_2_, CN, and C = O groups. Its ^1^H NMR spectrum showed three singlet signals at δ 12.10, 10.72, and 9.43 ppm for 3 NH, and a singlet signal integrated for 2 protons at δ 7.83 ppm corresponding for NH_2_. Like the behavior with ethylenediamine, the benzimidazole derivative **19** was formed from the reaction of the benzoxazinone **6** with 2-aminoaniline in refluxing ethanol containing acetic acid. Its ^1^H NMR spectrum exposed two singlet signals for 2 NH at δ 12.46 ppm and 8.99 ppm (cf. Figure 7). The suggested pathways for the formation of compounds **18** and **19** can be visualized via Figs. S2 and S3, respectively.

## Determination of total antioxidant capacity (TAC)

Most of the prepared compounds were tested for their in vitro antioxidant activity according to phosphomolybdenum method using ascorbic acid as a reference^32,33^. The total antioxidant capacity of a sample was measured in mg ascorbic acid equivalents (AAE)/g sample. The results in Table 1 showed that the benzimidazole derivative **19** exhibited very strong activity. In addition, compounds **3**, **9**, **13**, and **17** showed strong activity. Otherwise, compounds **4**, **7**, **10**, **16**, and **18** displayed moderate activity while the other compounds showed weak activity. The higher potency of compound **19** may be attributed to the aromaticity and extended conjugation^34,35^ integrated with the benzimidazole skeleton.

**Table 1:** Total antioxidant capacity (TAC) of the tested compounds.

Compds	Total antioxidant capacity (mg AAE/g compound)^1,2^
3	343.95 ± 3.01
4	249.01 ± 1.97
6	135.46 ± 1.65
7	237.18 ± 1.15
8	118.72 ± 1.99
9	311.15 ± 2.29
10	203.58 ± 1.38
12	180.34 ± 3.03
13	373.96 ± 1.15
16	256.82 ± 3.05
17	306.77 ± 1.99
18	240.76 ± 3.03
19	*426.95* ± *2.14*

^1^Results are (means ± S.D.) (n = 3).

^2^AAE (Ascorbic acid equivalent).

Italic value indicates the highest activity.

### Molecular docking study

**Fig. 8:**
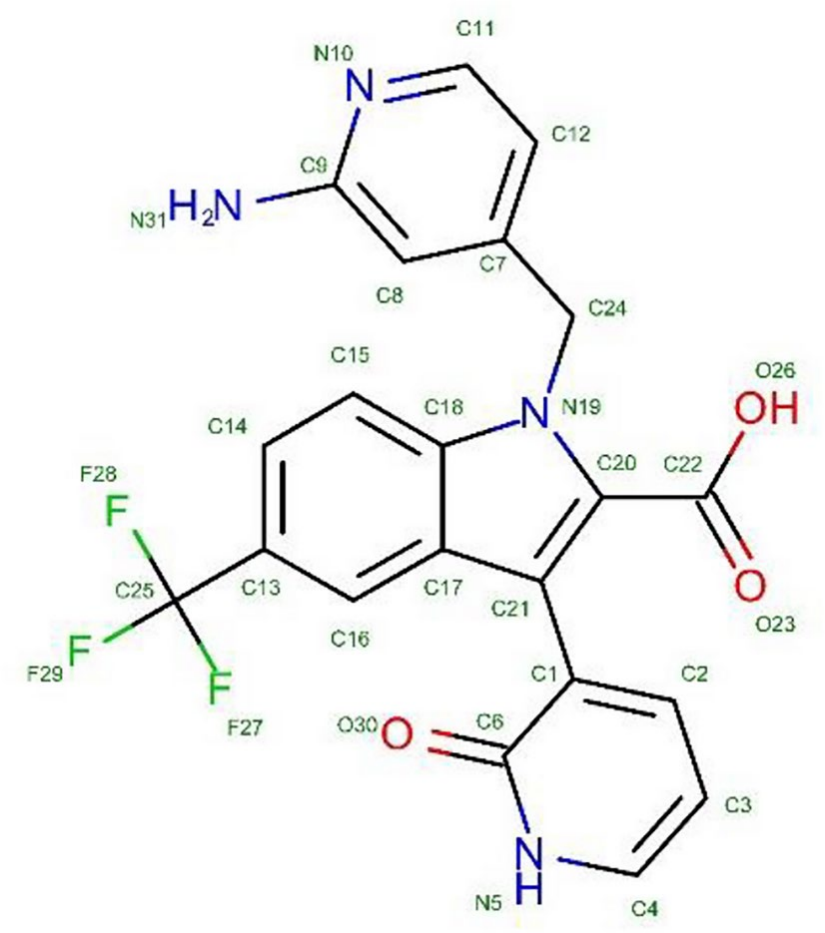
Co-crystallized ligand (053) of HCV NS5B polymerase.

A molecular docking simulation was performed to disclose the modes of interactions of the most potent benzimidazole **19** toward HCV NS5B polymerase (PDB ID: 3SKA, Resolution: 1.73 Å)^36^. The binding affinity was measured by the binding energies (S-score, kcal/mol) and hydrogen bonds. The synthesized complexes were docked in the same groove of binding site of native co-crystallized ligand (053) (cf. Figure 8).

The results of docking analysis of benzimidazole **19** with respect to the co-crystallized ligand (053) were depicted in 2D and 3D graphical representations (cf. Table S1). According to the data presented (cf. Table 2), ligand **19** exhibited binding energy of − 8.4812 kcal/mol, which was greater than that of co-crystallized ligand, referring to strong binding to certain key nucleobases and amino acids (CYS 366 and ASN 411) of HCV NS5B polymerase through hydrogen bonding and pi-hydrogen interactions, revealing its potential usage as an antioxidant agent.

**Table 2:** Docking results of compound **19** with respect to co-crystallized ligand (053) of HCV NS5B polymerase.

Compds	S-score (kcal/mol)	Ligand	Receptor	Interaction	Distance	E (kcal/mol)
**19**	− 8.4812	N 14	SG CYS 366	H-donor	3.17	− 2.7
		Cl 53	0D1 ASN 411	H-donor	3.40	− 0.8
		6-ring	CA CYS 366	pi-H	4.07	− 1.0
Co-crystalized ligand (053)	− 7.0234	O23 33	OG SER 556	H-acceptor	2.66	− 1.7
		O26 38	NE2 GLN 446	H-acceptor	2.74	− 3.8
		O26 38	N GLY 449	H-acceptor	2.79	− 1.6
		O30 42	N TYR 448	H-acceptor	2.98	− 2.5
		5-ring	CA TYR 448	pi-H	4.00	− 1.6

### DFT study

**Fig. 9:**
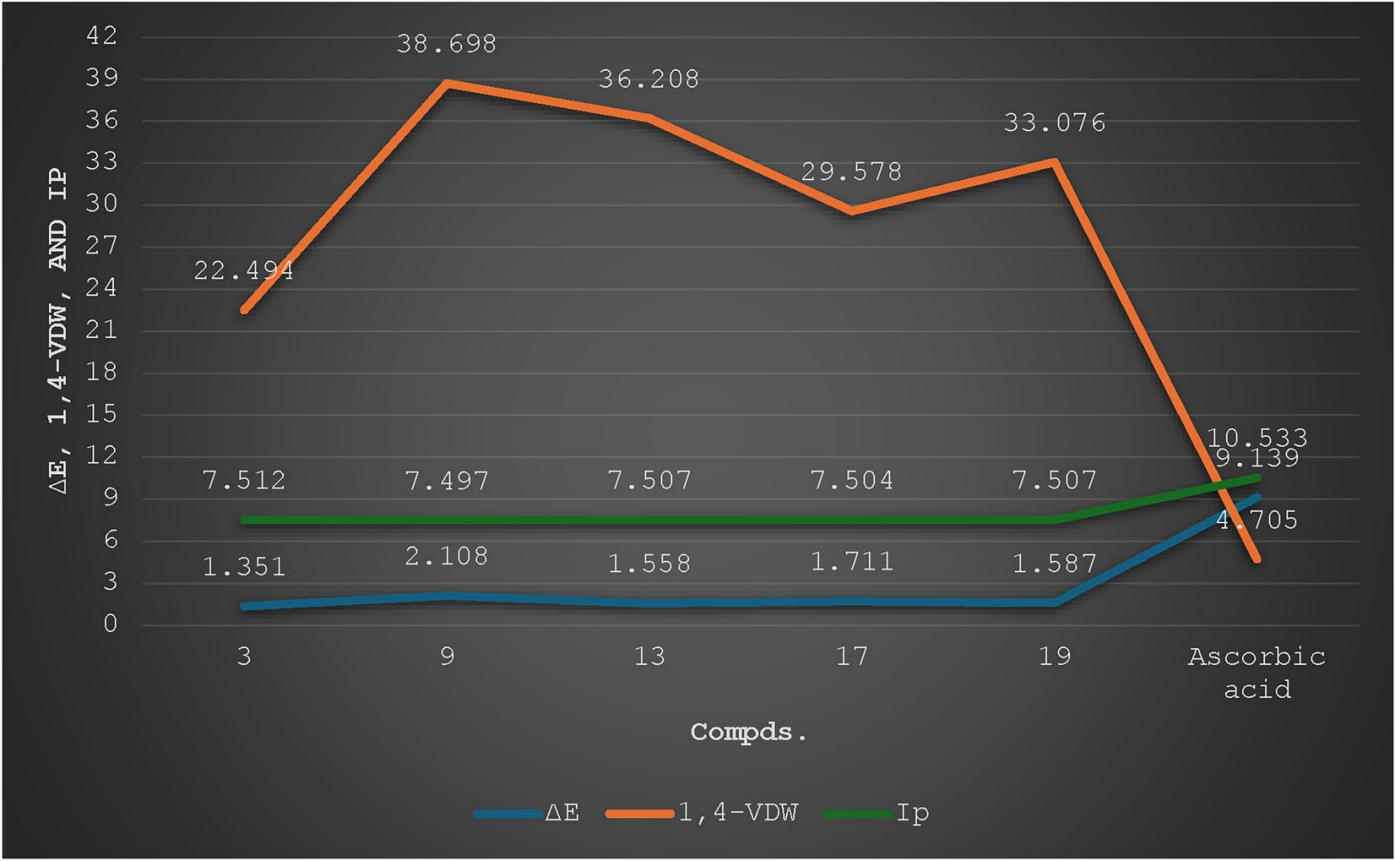
DFT parameters of the potent compounds.

DFT parameters and global descriptors were calculated for the most potent compounds to correlate the relation between chemical structure and activity (cf. Table S2 and Fig. 9)^37–39^. The structures of these compounds were drawn using ChemBio3D 14.0. The ∆E (E_LUMO_—E_HOMO_) values decrease in the order of ascorbic acid, **9**, **17**, **19**, **13**, **3**, respectively. Among them, the lowest ∆E value (1.351 kcal/mol) was discovered by compound **3**, making it more reactive towards radical surface interactions, being efficient of donating electrons easily to hole surface^40,41^ and therefore might display strong antioxidant efficiency. According to ionization potential (Ip, eV) values, compound **9** exhibited the lowest value (7.497 eV) which means that it is more feasible to remove electrons from its last orbitals, HOMO. Regarding the 1,4-VDW (van-der Waal) interactions, the values increase in the order of ascorbic acid, **3, 17, 19, 13,** and **9**, respectively. Compound **9** exhibited the highest 1,4-VDW value.

### Modeling pharmacokinetics study

The ADME profiles of the potent compounds including their physicochemical properties, lipophilicity, and drug-likeness were calculated in order to decrease the time required to select substrates from a huge collection of compounds in the early stages of drug discovery, biological activities, and development for potent drugs (cf. Table S3)^42–45^. The antioxidant activity revealed a range of TAC values which can be also attributed to compound’s ADME profile. TPSA (topological polar surface area) is a key property linked to drug bioavailability, which should be less than 140 Å^2^. The potent compounds **3**, **9**, **13**, **17**, and **19** exhibited TPSA of 73.98, 71.57, 111.95, 90.17, and 94.46 Å^2^, respectively, which are predicted to exhibit good passive oral absorption. Also, their consensus Log P_o/w_ values were 3.25, 5.52, 5.24, 4.26, and 5.62, respectively, which offered good lipophilicity and found to comply with Lipinski’s rule of five. Compounds **3**, **17**, and **19** showed no violations to Lipinski’s rule. Based on the pink area on the radar chart for substances (cf. Figures S4-S20), the bioavailability of those substances was also calculated.

According to calculations, compounds** 3**,** 9**, and **17** exhibited a high GI absorption and an excellent bioavailability score of 0.85, 0.55, and 0.55, respectively, as shown in Table S3. The variance in GI absorption might depict the differences in observed antioxidant efficacy of compounds. High GI absorption enables effective internal distribution and interaction with receptors, enhancing antioxidant activity. These compounds were fully included in the pink area, and this supported their well-predicted oral bioavailability. Their skin permeation (Log K_P_) parameters were -5.14, -4.38, -4.90, -5.44, and -4.12 cm/s, which made the bioactive compounds easier to access through the skin, protecting the body from free radical cell damage, supporting the immune system and assisting the body in the absorption process.

**Fig. 10:**
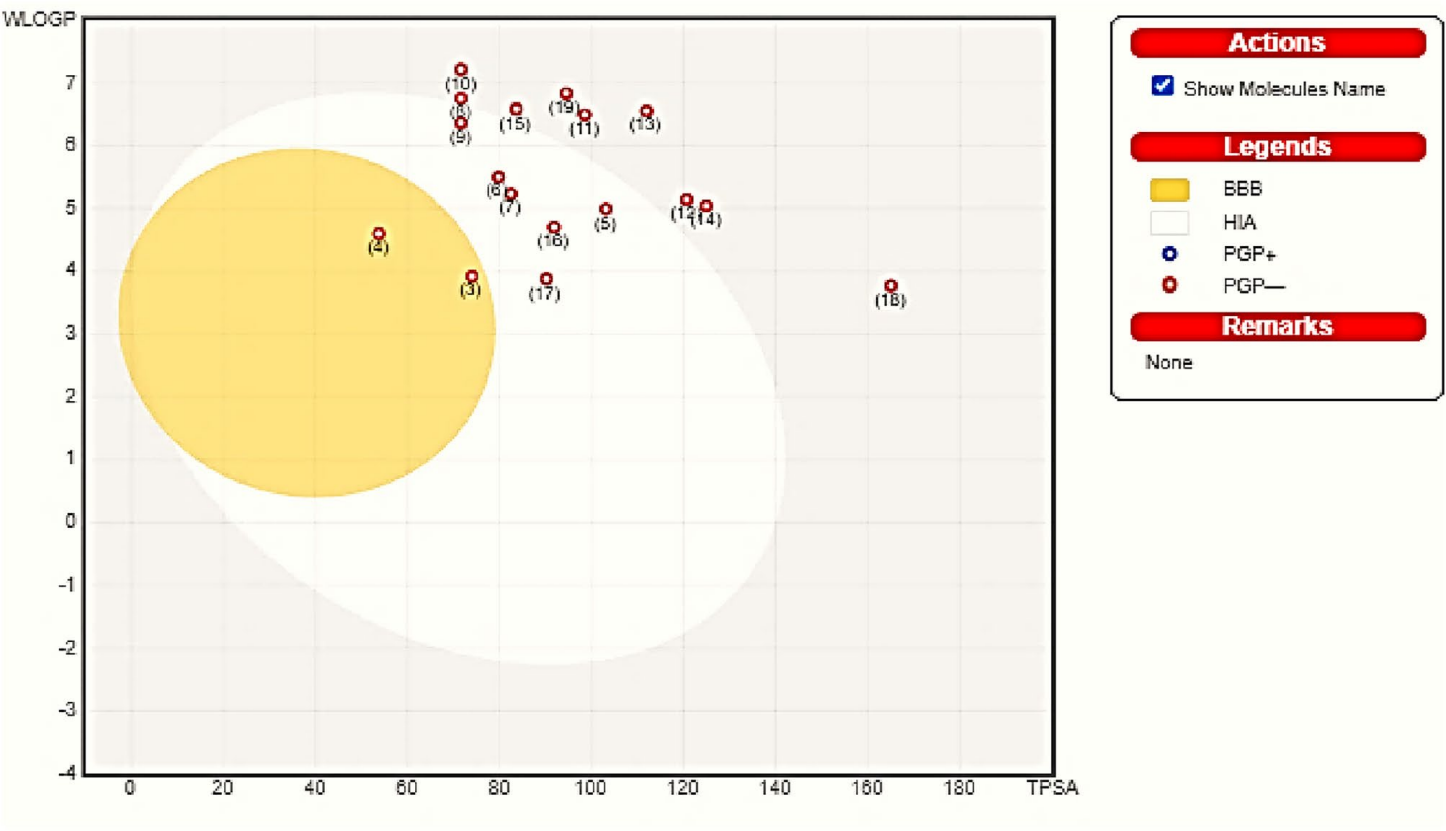
BOILED-EGG chart of compounds **3–19**.

Also, their cytochrome P450 isoenzymes (CYP1A2, CYP2D6, CYP2C9, CYP2C19, and CYP3A4), which play a substantial role in the biotransformation of medicines through *O*-type oxidation processes, were also estimated. All compounds are predicted as non-inhibitors of CYP2D6 and hence side effects (i.e. liver dysfunction) are not expected upon administration of them. Regarding the absorption property, compounds **5**, **6**,** 7**, **9**, **16**, and **17** showed gastrointestinal tract (GIT) absorption due to their being in the BOILED-EGG chart white area (cf. Figure 10). Also, compounds **3** and **4** are expected to penetrate the blood–brain barrier (BBB), existed inside chart yellow area, which selectively regulates the permeability of drugs to the brain. Furthermore, they are not potential substances for permeability glycoprotein (PGP) which is indicated by red.

**Fig. 11:**
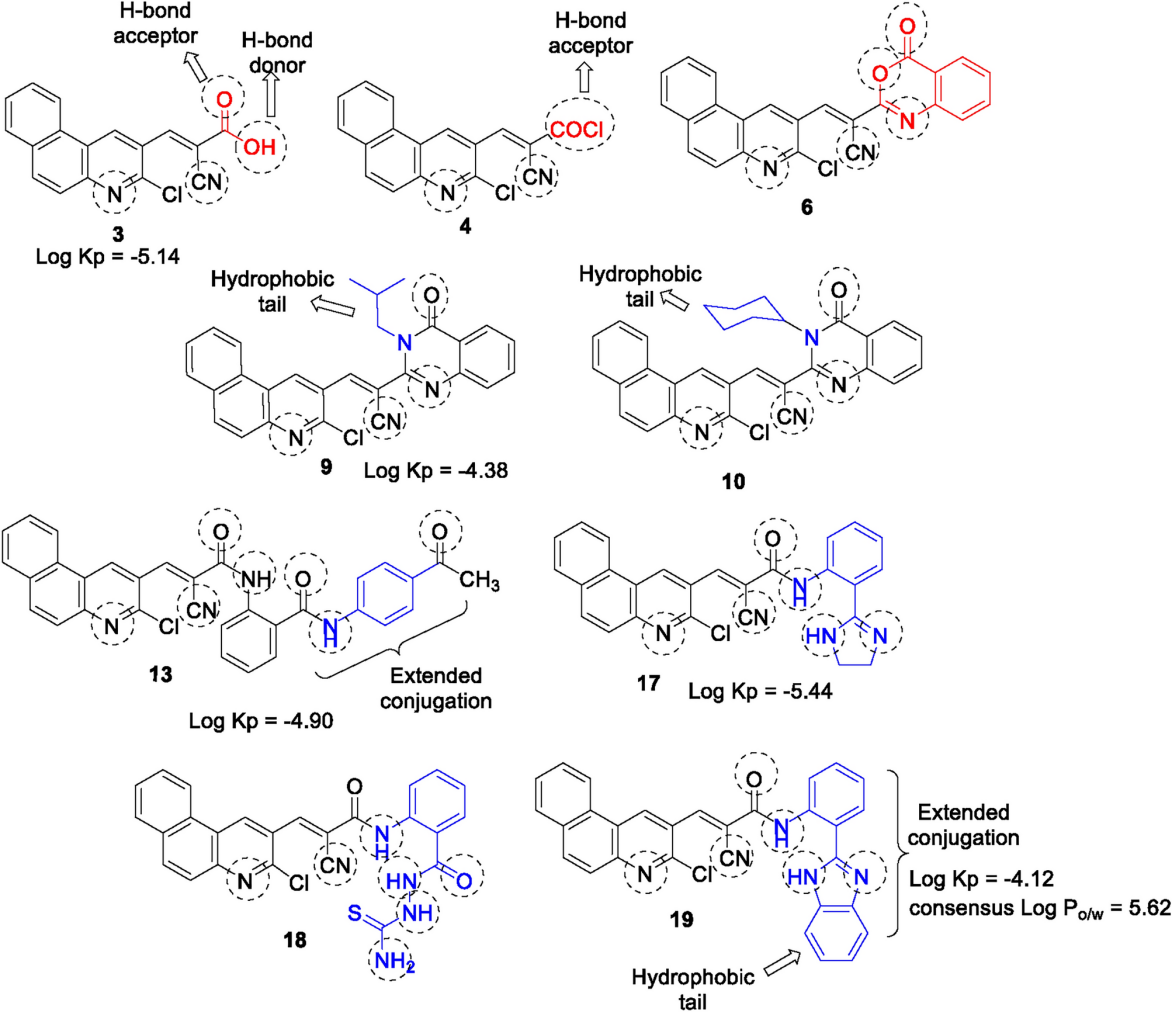
SAR and in silico pharmacokinetics and drug-likeness properties.

Regarding SAR of the antioxidant properties, it was noted that the active compounds satisfy the Lipinsk, Veber, and Ghose rules, with no violations. They exhibited a higher number of H-bond donors and acceptors (cf. Figure 11 and Table S3). Also, they exhibited higher values of bioavailability score, Log K_P_ (skin permeation), and consensus Log P_o/w_ (lipophilicity). The benzimidazole **19** had the highest consensus Log P_o/w_ value (5.62), which strongly enhanced lipophilicity and the binding interactions with receptors (cf. Table 2). Also, the existence of tautomerism in compounds increases antioxidant activity via increasing hydrogen bonding interactions. Also, the extended conjugation increases the scavenging activity by enhancing the lipophilicity properties.

## Materials and methods

“Melting points are uncorrected and were measured on a MEL-TEMP II electric melting point apparatus. The IR spectra (*ν*, cm^-1^) were recorded by KBr disks on Fourier Transform Infrared Thermo Electron Nicolet iS10 Spectrometer (Thermo Fisher Scientific Inc., Waltham, MA, USA) at Faculty of Science, Ain Shams University. The ^1^H and ^13^C NMR spectra (δ, ppm) were run at a BRUKER 400 and 100 *MHz* using tetramethyl silane as an internal standard in deuterated dimethyl sulfoxide (DMSO-*d*_*6*_) at Faculty of Pharmacy, Ain Shams University. Electron impact mass spectra were carried out on direct probe controller inlet part to single quadrupole mass analyzer in Thermo Scientific GCMS MODEL (ISQ LT) using Thermo X-CALIBUR software at the regional center for mycology and biotechnology (RCMB), Azhar University, Cairo, Egypt. Elemental analyses were measured using Perkin-Elmer 2400 CHN elemental analyzer at Faculty of Science, Ain Shams University, and satisfactory analytical data (±0.4) were obtained. The reactions were monitored by thin-layer chromatography (TLC) using Merck Kiesel gel 60F_254_ obtained from Fluka, Switzerland.”

### Ethyl 3-(3-chlorobenzo[f]quinolin-2-yl)-2-cyanoacrylate (2)

A mixture of 3-chlorobenzo[*f*]quinoline-2-carbaldehyde **(1)** (0.01 mol, 2.41 g) and ethyl cyanoacetate (0.01 mol, 1.13 ml) in ethanol (20 ml) and piperidine (0.1 mL) was refluxed for 30 min. The formed product was filtered, washed with ethanol, dried, and recrystallized from ethanol to afford beige crystals, mp 206–208 °C, Yield (90%). Anal. Calcd. for C_19_H_13_ClN_2_O_2_ (336.8): C, 67.76; H, 3.89; N, 8.32; Found: C, 67.74; H, 3.92; N, 8.35%. FTIR (ν, cm^-1^): 2224 (CN), 1712 (C = O ester), 1621 (C = N). ^1^H NMR (DMSO-*d*_6_, *δ*, ppm): 9.10 (s, 1H, CH olefinic), 9.02 (d, 1H, C5-Ar, *J* = 7.4 Hz), 8.62 (s, 1H, C4-H quinoline), 8.11–7.86 (m, 5H, Ar–H), 4.39 (q, 2H, CH_2_, *J* = 6.7 Hz), 1.35 (t, 3H, CH_3_, *J* = 6.7 Hz). ^13^C NMR (DMSO-*d*_6_, *δ*, ppm): 191.1, 161.4, 150.1, 148.9, 147.1, 139.5, 130.7, 129.8 (2), 128.9 (2), 128.5 (2), 125.5 (2), 124.7, 108.4, 63.4, 14.5.

### 3-(3-Chlorobenzo[f]quinolin-2-yl)-2-cyanoacrylic acid (3)

To a solution of ethyl-3-(3-chlorobenzo[*f*]quinolin-2-yl)-2-cyanoacrylate **(2)** (0.01 mol, 3.37 g) in ethyl alcohol (20 ml), sodium hydroxide (0.01 mol, 0.4 g) in ethyl alcohol (3 ml) was added and then the reaction mixture was stirred for 6 h at room temperature. The reaction mixture was poured onto crushed ice and diluted solution of HCl. The formed precipitate was filtered, dried, and recrystallized from dioxane to form a yellow precipitate, mp. > 300 oC, Yield (90%). Anal. Calcd. for C_17_H_9_ClN_2_O_2_ (308.7): C, 66.14; H, 2.94; N, 9.07; Found: C, 66.18; H, 2.90; N, 9.11%. FTIR (ν, cm^-1^): 3440 (*br*. OH), 2225 (CN), 1706 (C = O acid). ^1^H NMR (DMSO-*d*_6_, *δ*, ppm): 9.05 (s, 1H, CH olefinic), 8.54 (s, 1H, C4-H quinoline), 8.97 (d, 1H, C_5_-Ar, *J* = 7.6 Hz), 8.10–7.80 (m, 6H, Ar–H). MS, *m*/*z* (%): 308 (12), 265 (61), 116 (28), 77 (100).

### 3-(3-Chlorobenzo[f]quinolin-2-yl)-2-cyanoacryloyl chloride (4)

A mixture of acid derivative **3** (0.01 mol, 3.08 g) and thionyl chloride (15 ml) was heated on water bath for 8 h. The reaction mixture was left until all thionyl chloride evaporated. The formed precipitate was washed with hot petroleum ether (60–80) to furnish a yellow solid, mp. 252–254 oC, Yield (85%). Anal. Calcd. for C_17_H_8_Cl_2_N_2_O (327.2): C, 62.41; H, 2.46; N, 8.56; Found: C, 62.45; H, 2.50; N,8.52%. FTIR (ν, cm^-1^): 2224 (CN), 1761, 1715 (C = O). ^1^H NMR (DMSO-*d*_6_, *δ*, ppm): 9.01 (s, 1H, CH olefinic), 8.93 (d, 1H, CH, C_5_-Ar, *J* = 7.8 Hz), 8.50 (s, 1H, C4-H quinoline), 8.07–7.97 (m, 5H, Ar–H). ^13^C NMR (DMSO-*d*_6_, *δ*, ppm): 162.7, 152.7, 149.2, 148.8, 147.1, 146.9, 139.1, 130.5, 129.6, 128.7, 128.4, 125.3, 125.2, 124.6, 115.4, 114.8, 113.1, 109.5, 103.5, 99.9. MS, *m*/*z* (%): 327 (22), 283 (100), 257 (40), 78 (92).

### 2-(3-(3-Chlorobenzo[f]quinolin-2-yl)-2-cyanoacrylamido)benzoic acid (5)

A mixture of acid chloride derivative **4** (0.01 mol, 3.7 g) and anthranilic acid (0.01 mol, 1.37 g) in dry dioxane (20 ml) containing triethylamine (0.1 mL) was stirred at room temperature for 10 h. The solid obtained was filtered, dried, and recrystallized from dioxane to provide yellow crystals, mp.180–182 oC, Yield (82%). Anal. Calcd. for C_24_H_14_ClN_3_O_3_ (427.8): C, 67.38; H, 3.30; N, 9.82; Found: C, 67.34; H, 3.33; N, 9.86%. FTIR (ν, cm^-1^): 3422 (*br*. OH), 3212, 3172 (NH), 2207 (CN), 1695 (C = O acid), 1667 (C = O amide). ^1^H NMR (DMSO-*d*_6_, *δ*, ppm): 12.85 (*br*.s, 1H, OH, exchangeable), 9.20 (*br*.s, 1H, NH, exchangeable), 9.15 (s, 1H, CH olefinic), 8.66 (s, 1H, C4-H quinoline), 8.64–723 (m, 10H, Ar–H). ^13^C NMR (DMSO-*d*_6_, *δ*, ppm): 179.9, 169.5 (C = O), 158.1 (C = N), 147.1 (C = C), 140.1, 138.9, 134.4, 133.7, 131.2, 130.0 (2), 129.2 (2), 128.3 (2), 127.8, 125.4 (2), 124.9 (2), 124.2, 123.9, 120.3, 117.0 (CN).

### 3-(3-Chlorobenzo[f]quinolin-2-yl)-2-(4-oxo-4H-benzo[d][1,3]oxazin-2-yl)acrylonitrile (6)

A suspension of benzoic acid derivative **5** (0.01 mol, 4.27 g) in acetic anhydride (25 ml) was heated on water steam for 6 h. The formed precipitate was filtered, dried, and recrystallized from benzene to yield a pale-yellow precipitate, mp. 280–282 oC, Yield (78%). Anal. Calcd. for C_24_H_12_ClN_3_O_2_ (409.8): C, 70.34; H, 2.95; N, 10.25; Found: C, 70.38; H, 2.90; N, 10.19%. The FTIR (ν, cm^-1^): 2226 (CN), 1774 (C = O), 1620 (C = N). ^1^H NMR (DMSO-*d*_6_, *δ*, ppm): 9.2 (s, 1H, CH olefinic), 8.69 (s, 1H, C4-H quinoline), 9.05 (d, 1H, C5-Ar, *J* = 8.3 Hz), 8.22–7.37 (m, 9H, Ar–H). ^13^C NMR (DMSO-*d*_6_, *δ*, ppm): 170.4, 158.4, 158.1, 148.9, 147.8, 147.0, 145.9, 139.4, 134.9, 130.6 (2), 130.2, 129.8 (2), 129.5 (2), 128.8 (2), 128.5 (2), 125.5 (2), 125.1, 124.6. MS, *m*/*z* (%): 411 (M^+.^ + 1, 19), 367 (44), 225 (10), 137 (18), 90 (29), 60 (100).

### 3-(3-Chlorobenzo[f]quinolin-2-yl)-2-(4-oxo-3,4-dihydroquinazolin-2-yl)acrylonitrile (7)

A mixture of benzoxazine derivative **6** (0.002 mol, 1 g) and ammonium acetate (2 g) was fused for 8 h. The formed precipitate was filtered, washed with ethanol, dried, and recrystallized from dioxane to form yellow crystals, mp. > 300 °C, Yield (80%). Anal. Calcd. for C_24_H_13_ClN_4_O (408.8): C, 70.51; H, 3.21; N,13.70; Found: C, 70.55; H, 3.18; N, 13.72%. FTIR (cm^-1^): 3338 (NH), 2213 (CN), 1689 (C = O amide), 1624 (C = N). ^1^H NMR (DMSO-*d*_6_, *δ*, ppm): 10.42 (*br*.s, 1H, NH, exchangeable), 9.10 (s, 1H, CH olefinic), 8.59 (s, 1H, C4-H quinoline), 8.53 (d, 1H, Ar–H, *J* = 7.9 Hz), 8.13–7.07 (m, 9H, Ar–H). MS, *m*/*z* (%): 408 (50), 354 (95), 313 (56), 298 (100), 220 (63), 164 (85), 91 (47).

### 2-(3-Benzyl-4-oxo-3,4-dihydroquinazolin-2-yl)-3-(3-chlorobenzo[f]quinolin-2-yl)acrylonitrile (8)

To a solution of benzoxazine derivative** 6** (0.002 mol, 1 g) in n-butanol (20 ml), benzylamine (0.002 mol, 0.21 ml) was added and then the reaction mixture was refluxed for 6 h. The precipitated solid was filtered and washed with *n*-butanol, dried, and recrystallized from dioxane to produce pale-yellow crystals, mp. 242–245 °C, Yield (85%). Anal. Calcd. for C_31_H_19_ClN_4_O (499): C, 74.62; H, 3.84; N, 11.23; Found: C, 74.68; H, 3.79; N, 11.27%. FTIR (ν, cm^-1^): 2207 (CN), 1687 (C = O amide), 1621 (C = N). ^1^H NMR (DMSO-*d*_6_, *δ*, ppm): 9.03 (s, 1H, CH olefinic), 8.96 (d, 1H, C_5_-H), 8.56 (s, 1H, C4-H quinoline), 8.03–7.36 (m, 12H, Ar–H), 7.09 (t, 2H, Ph-H, *J* = 7.6 Hz), 3.40 (s, 2H, CH_2_). ^13^C NMR (DMSO-*d*_6_, *δ*, ppm): 169.9 (C = O), 157.8, 148.23 (C = N), 146.15 (C = C), 145.6, 140.4, 138.7, 134.2, 131.1, 130.3, 129.8 (2), 129.1 (2), 129.0, 128.3 (2), 127.9 (2), 125.6, 125.1 (2), 124.9, 124.6 (2), 124.0 (2), 122.67, 119.04, 114.9, 113.4 (Ar–C).

### 3-(3-Chlorobenzo[f]quinolin-2-yl)-2-(3-isobutyl-4-oxo-3,4-dihydroquinazolin-2-yl)acrylonitrile (9)

A mixture of benzoxazine **6** (0.002 mol, 1 g) and isobutylamine (0.002 mol, 0.15 ml) in ethanol (20 ml) containing glacial acetic acid (0.1 mL) was refluxed for 10 h. The solid obtained was filtered off and recrystallized from dioxane to produce yellow crystals, mp. 268–270 °C, Yield (90%). Anal. Calcd. for C_28_H_21_ClN_4_O (465): C, 72.33; H, 4.55; N, 12.05; Found: C, 72.36; H, 4.50; N, 12.09%. FTIR (ν, cm^-1^): 2210 (CN), 1689 (C = O imide), 1611 (C = N). ^1^H NMR (DMSO-*d*_6_, *δ*, ppm): 9.13 (s, 1H, CH olefinic), 8.61 (s, 1H, C4-H quinoline), 9.03 (d, 1H, C_5_-H, *J* = 7.4 Hz), 8.56–7.12 (m, 9H, Ar–H), 3.29 (d, 2H, CH_2_, *J* = 6.4 Hz), 2.62 (m, 1H, CH), 0.90 (d, 6H, 2CH_3_, *J* = 6.8 Hz). MS, *m*/*z* (%): 465 (M^+.^, 7.6), 417 (75), 371 (100), 126 (66), 105 (43).

### 3-(3-Chlorobenzo[f]quinolin-2-yl)-2-(3-cyclohexyl-4-oxo-3,4-dihydroquinazolin-2-yl)acrylonitrile (10)

A mixture of benzoxazine **6** (0.002 mol, 1 g) and cyclohexylamine (0.002 mol, 0.2 g) in ethanol (20 ml) containing glacial acetic acid (0.1 mL) was refluxed for 10 h. The solid obtained was filtered, dried, and recrystallized from dioxane to produce yellow crystals, mp. 272–274 oC, Yield (88%). Anal. Calcd. for C_30_H_23_ClN_4_O (491): C, 73.39; H, 4.72; N, 11.41; Found: C, 73.44; H, 4.75; N, 11.37%. FTIR (ν, cm^-1^): 2207 (CN), 1693 (C = O amide), 1629 (C = N). ^1^H NMR (DMSO-*d*_6_, *δ*, ppm): 9.15 (s, 1H, CH olefinic), 8.63 (s, 1H, C4-H quinoline), 9.03 (d, 1H, C_5_-H, J = 7.4 Hz), 8.50–7.27 (m, 9H, Ar–H), 3.85 (m, 1H, CH–N), 1.86–1.35 (m, 10H, 5CH_2_ cyclohexyl ring). ^13^C NMR (DMSO-*d*_6_, *δ*, ppm): 194.0, 187.8, 167.6, 158.4, 147.9, 147.1, 139.6, 138.5, 129.8 (2), 128.9 (2), 128.6 (2), 125.6 (2), 121.6, 114.7, 103.1, 99.9, 40.6, 40.4, 40.3, 40.1, 39.9, 39.7 (2), 25.7, 25.4 (2). MS, *m*/*z* (%): 490 (18), 330 (24), 283 (100), 230 (50), 135 (36).

### 3-(3-Chlorobenzo[f]quinolin-2-yl)-2-(3-(1,5-dimethyl-3-oxo-2-phenyl-2,3-dihydro-1H-pyrazol-4-yl)-4-oxo-3,4-dihydroquinazolin-2-yl)acrylonitrile (11)

A solution of benzoxazine **6** (0.002 mol, 1 g) and 4-amino-1,5-dimethyl-2-phenyl-1,2-dihydro-3*H*-pyrazol-3-one (0.002 mol, 0.4 g) in dioxane (15 ml) was refluxed for 8 h. The obtained solid was filtered, dried, and recrystallized from ethanol to furnish orange crystals, mp. 263–265 °C, Yield (55%). Anal. Calcd. for C_35_H_23_ClN_6_O_2_ (595.1): C, 70.65; H, 3.90; N, 14.12; Found: C, 70.61; H, 3.93; N, 14.15%. FTIR (ν, cm^-1^): 2210 (CN), 1697 (C = O pyrazalone), 1650 (C = O imide), 1615 (C = N). ^1^H NMR (DMSO-*d*_6_, *δ*, ppm): 9.13 (s, 1H, CH olefinic), 9.02 (d, 1H, C5-H, *J* = 7.3 Hz), 8.63 (s, 1H, C4-H quinoline), 8.58–7.83 (m, 14H, Ar–H), 3.19 (s, 3H, CH_3_N), 1.20 (s, 3H, CH_3_C = C).

### 2-(3-(3-Chlorobenzo[f]quinolin-2-yl)-2-cyanoacrylamido)-N-(pyrimidin-2-yl)benzamide (12)

A mixture of benzoxazine **6** (0.002 mol, 1 g) and 2-aminopyrimidine (0.002 mol, 0.19 g) in dioxane (15 ml) was refluxed for 6 h. The precipitated solid was filtered off, dried, and recrystallized from dioxane to produce orange precipitate, mp. > 300 °C, Yield (85%). Anal. Calcd. for C_28_H_17_ClN_6_O_2_ (504.9): C, 66.60; H, 3.39; N, 16.64; Found: C, 66.63; H, 3.42; N, 16.61%. FTIR (ν, cm^-1^): 3333, 3186 (NH), 2212 (CN), 1678 (C = O). ^1^H NMR (DMSO-*d*_6_, *δ*, ppm): 12.69 (*br*.s, 1H, NH, exchangeable), 9.13 (s, 1H, CH olefinic), 9.02 (s, 1H, NHCO, exchangeable), 8.67 (s, 1H, C4-H quinoline), 8.62–6.51 (m, 13H, Ar–H). MS, *m*/*z* (%): 504 (21), 395 (36), 368 (100), 342 (68), 236 (66), 125 (42).

### N-(4-Acetylphenyl)-2-(3-(3-chlorobenzo[f]quinolin-2-yl)-2-cyanoacrylamido)benzamide (13)

Benzoxazine derivative **6** (0.002 mol, 1 g) was added to a solution of 4-aminoacetophenone (0.002 mol, 0.27 g) in dioxane (15 ml) containing glacial acetic acid (0.1 mL) was heated under reflux for 6 h. The deposited solid was collected and recrystallized from dioxane to form orange crystals, mp. 282–284 °C, Yield (75%). Anal. Calcd. for C_32_H_21_ClN_4_O_3_ (545): C, 70.52; H, 3.88; N, 10.28; Found: C, 70.48; H, 3.91; N, 10.23%. FTIR (ν, cm^-1^): 3325, 3165 (NH), 2222 (CN), 1727 (C = O acetyl), 1648 (C = O amide), 1626 (C = N). ^1^H NMR (DMSO-*d*_6_, *δ*, ppm): 12.71 (br.s, 1H, ArNHCO, exchangeable), 9.13 (s, 1H, CH olefinic), 8.90 (br.s, 1H, NHCO, exchangeable), 8.50 (s, 1H, C4-H quinoline), 7.98–7.64 (m, 14H, Ar–H), 2.47 (s, 3H, CH_3_). MS, *m*/*z* (%): 545 (14), 485 (72), 323 (52), 292 (64), 262 (100), 180 (96), 108 (48).

### 3-(3-Chlorobenzo[f]quinolin-2-yl)-2-cyano-N-(2-(5-oxo-4,5-dihydro-1,3,4-oxadiazol-2-yl)phenyl)acrylamide (14)

A mixture of benzoxazine **6** (0.002 mol, 1 g) and ethyl hydrazinecarboxylate (0.002 mol, 0.2 g) in dioxane (15 ml) was refluxed for 6 h. The formed solid was filtered, dried, and recrystallized from dioxane to produce yellow precipitate, mp. > 300 °C, Yield (60%). Anal. Calcd. for C_25_H_14_ClN_5_O_3_ (467.9): C, 64.18; H, 3.02; N, 14.97; Found: C, 64.21; H, 3.06; N, 14.92%. FTIR (ν, cm^-1^):3233 (NH), 2214 (CN),1694 (C = O oxadiazolone), 1670 (C = O amide). ^1^H NMR (DMSO-*d*_6_, *δ*, ppm): 13.04 (*br*.s, 1H, NH, exchangeable), 9.01 (s, 1H, CH olefinic), 8.92 (*br*.s, 1H, NHCO, exchangeable), 8.59 (s, 1H, C4-H quinoline), 8.57 (d, 1H, C_5_-H, *J* = 8.6 Hz), 8.04–7.76 (m, 9H, Ar–H).

### 3-(3-Chlorobenzo[f]quinoline-2-yl)-2-(4-oxo-3-(phenylamino)-3,4-dihydroquinazolin-2-yl) acrylonitrile (15)

Phenylhydrazine (0.002 mol, 0.26 ml) was added to a solution of benzoxazine **6** (0.002 mol, 1 g) in dioxane (15 ml) and then, the reaction mixture was refluxed for 8 h. The deposited solid was collected and recrystallized from dioxane to produce yellow crystals, mp. > 300 °C, Yield (75%). Anal. Calcd. for C_30_H_18_ClN_5_O (500): C, 72.07; H, 3.63; N,14.01. Found: C, 72.02; H, 3.65; N, 13.97%. FTIR (ν, cm^-1^): 3341 (NH), 2216 (CN), 1691 (C = O amide), 1605 (C = N). ^1^H NMR (DMSO-*d*_6_, *δ*, ppm): 13.38 (*br*.s, 1H, NHPh, exchangeable), 9.1 (s, 1H, CH olefinic), 8.99 (d, 1H, C_5_-H, *J* = 7.8 Hz), 8.64 (s, 1H, C4-H quinoline), 8.12–7.21 (m, 14H, Ar–H). MS, *m*/*z* (%): 500 (M^+.^, 16), 480 (32), 452 (90), 340 (39), 263 (100), 162 (52), 79 (56).

### 3-(3-Chlorobenzo[f]quinolin-2-yl)-2-(3-(2-hydroxyethyl)-4-oxo-3,4-dihydroquinazolin-2-yl)acrylonitrile (16)

A mixture of benzoxazine **6** (0.002 mol, 1 g) and ethanolamine (0.002 mol, 0.12 ml) in dioxane (15 ml) was refluxed for 6 h. The precipitated solid was filtered and recrystallized from dioxane to furnish yellow crystals, mp. > 300 °C, Yield (55%). Anal. Calcd. for C_26_H_17_ClN_4_O_2_ (452.9): C, 68.95; H, 3.78; N, 12.37; Found: C, 68.90; H, 3.73; N, 12.41%. FTIR (ν, cm^-1^): 2210 (CN), 1679 (C = O amide), 1645 (C = N). ^1^H NMR (DMSO-*d*_6_, *δ*, ppm): 9.19 (s, 1H, CH olefinic), 8.70 (s, 1H, C4-H quinoline), 9.11–7.20 (m, 10H, Ar–H), 3.73 (*br*.s, 1H, OH, exchangeable), 3.02 (t, 2H, CH_2_CH_2_OH, *J* = 6.7 Hz), 2.61 (t, 2H, CH_2_CH_2_OH, *J* = 6.8 Hz). MS, *m*/*z* (%): 452 (M^+.^, 30), 381 (43), 334 (100), 264 (89), 199 (66), 117 (40).

### 3-(3-Chlorobenzo[f]quinolin-2-yl)-2-cyano-N-(2-(4,5-dihydro-1H-imidazol-2-yl)phenyl)acrylamide (17)

Ethylenediamine (0.002 mol, 0.12 ml) was added to a solution of benzoxazine **6** (0.002 mol, 1 g) in ethanol (20 ml) and then, the reaction mixture was refluxed for 8 h. The precipitated solid was filtered and recrystallized from dioxane to produce brown crystals, mp. 260–262 °C, Yield (65%). Anal. Calcd. for C_26_H_18_ClN_5_O (451.9): C, 69.10; H, 4.01; N, 15.50; Found: C, 69.15; H, 4.04; N, 15.46%. FTIR (ν, cm^-1^): 3365 (NH), 2211 (CN), 1681 (C = O amide). ^1^H NMR (DMSO-*d*_6_, *δ*, ppm): 13.20 (*br*.s, 1H, NH imidazole, exchangeable), 9.03 (s, 1H, CH olefinic), 8.97 (*br*.s, 1H, NHCO, exchangeable), 8.57 (s, 1H, C4-H quinoline), 8.07–7.76 (m, 10H, Ar–H), 3.99 (t, 2H, CH_2_N = C, *J* = 7.2 Hz), 3.22 (t, 2H, CH_2_NH, *J* = 6.9 Hz). MS, *m*/*z* (%): 451 (M^+.^, 100), 436 (54), 322 (47), 163 (21), 128 (30).

### N-(2-(2-Carbamothioylhydrazine-1-carbonyl)phenyl)-3-(3-chlorobenzo[f]quinolin-2-yl)-2-cyanoacrylamide (18)

A mixture of benzoxazine **6** (0.002 mol, 1 g) and thiosemicarbazide (0.002 mol, 0.18 g) in ethanol (20 ml) containing glacial acetic acid (0.1 mL) was refluxed for 10 h. The precipitated solid was filtered, dried, and recrystallized from ethanol to furnish pale-brown crystals, mp. > 300 oC, Yield (69%). Anal. Calcd. for C_25_H_17_ClN_6_O_2_S (501): C, 59.94; H, 3.42; N, 16.78; Found: C, 59.90; H, 3.45; N, 16.74%. FTIR (cm^-1^): 3448 (NH), 3314, 3273 (NH_2_) 2204 (CN), 1688, 1650 (C = O amide), 1613 (C = N). ^1^H NMR (DMSO-*d*_6_, *δ*, ppm): 12.10 (s, 1H, NHCO, exchangeable), 10.72 (s, 1H, CONHNH, exchangeable), 9.43 (s, 1H, CONHNH, exchangeable), 9.12 (s, 1H, CH olefinic), 8.63 (s, 1H, C4-H quinoline), 8.38–7.30 (m, 10H, Ar–H), 7.83 (s, 2H, NH_2_, exchangeable). MS, *m*/*z* (%): 500 (M^+.^, 42), 340 (44), 300 (100), 281 (64), 209 (70), 147 (34), 99 (36).

### N-(2-(1H-Benzo[d]imidazol-2-yl)phenyl)-3-(3-chlorobenzo[f]quinolin-2-yl)-2-cyanoacrylamide (19)

A mixture of benzoxazine derivative **6** (0.002 mol, 1 g) and *o*-phenylenediamine (0.002 mol, 0.22 g) in ethanol (10 ml) containing acetic acid (0.1 mL) was refluxed for 6 h. The precipitated solid was filtered and recrystallized from dioxane to produce pale-yellow crystals, mp. > 300 °C, Yield (73%). Anal. Calcd. for C_30_H_18_ClN_5_O (500): C, 72.07; H, 3.63; N,14.01; Found: C, 72.02; H, 3.65; N,13.97%. FTIR (ν, cm^-1^): 2209 (CN), 1688 (C = O amide), 1606 (C = N). ^1^H NMR (DMSO-*d*_6_, *δ*, ppm): 12.46 (*br*.s, 1H, NH benzimidazole, exchangeable), 9.1 (s, 1H, CH olefinic),8.99 (*br*.s, 1H, NHCO, exchangeable), 8.66 (s, 1H, C4-H quinoline), 8.06–7.25 (m, 14H, Ar–H). MS, *m*/*z* (%): 500 (M^+.^, 52), 447 (60), 396 (68), 354 (95), 232 (88), 150 (82), 129 (100), 90 (80).

### Determination of total antioxidant capacity (TAC)

“Antioxidant activity of each extract was investigated via phosphomolybdenum method using ascorbic acid as a standard. This assay is based on the reduction of Mo (VI) to Mo (V) by the sample analyte and subsequent formation of a green-colored [phosphate=Mo (V)] complex at pH< 7 with maximal absorption at 695 nm. In this method, 0.5 mL of each extract (200 µg/mL) in methanol was combined in dried vials with 5 mL of reagent solution (0.6 M sulfuric acid, 28 mM sodium phosphate, and 4 mM ammonium molybdate). The vials of the reaction mixture were capped and incubated in a thermal block at 95 °C for 90 min^46^. After the samples had cooled at room temperature, the absorbance was measured at 695 nm against a blank experiment. All experiments were executed in triplicate. The antioxidant activity of the sample was formulated as the number of ascorbic acid equivalent (AAE)^30,31^. All data were recorded as mean ± S.D. using Graph Pad Prism software version 7 (https://www.graphpad.com). Differences between groups were considered statistically significant at *p* values < 0.05”.

### Molecular docking

In the docking study, the crystal structure of the binding protein, HCV NS5B polymerase was retrieved from the Protein Data Bank (https://www.rcsb.org, PDB ID: 3SKA, Resolution: 1.73 Å). AutoDock Vina (ADT, Version 1.5.7) available from Scripps Research Institute (http://autodock.scripps.edu/resources/adt) was employed to perform molecular docking study, focusing on the interaction between ascorbic acid, and the potent compound with HCV NS5B polymerase. To prepare the protein receptor, necessary steps such as 3D hydrogenation and energy minimization were carried out. Docking results were analyzed using Biovita Discovery Studio Visualizer (https://biovia-discovery-studio-64-bit-client.software.informer.com/4.5/).

### Modeling pharmacokinetics

The ADME profiles of the synthesized compounds including their physicochemical properties, lipophilicity, and drug-likeness were obtained from the SwissADME free web tool (http://www.swissadme.ch/).

## Supplementary Information

Supplementary Information 1.

Supplementary Information 2.

Supplementary Information 3.

## Data Availability

All data generated or analyzed during this study are included in this published article and its supplementary information files.
