# Supplementary material for: Antioxidant activity, molecular docking, and modeling pharmacokinetics study of some benzo[f]quinoline candidates
Source: Sci Rep. 2025 May 13;15:16522. doi: 10.1038/s41598-025-99811-1 (PMC12075698; doi:10.1038/s41598-025-99811-1)
Supplement: Supplementary file 1 — Supplementary Information 1. [file 41598_2025_99811_MOESM1_ESM.pdf]

Antioxidant Activity, Molecular Docking, and Modeling Pharmacokinetics Study of Some Benzo[*f*]quinoline Candidates

Sara F. El-Fagal [1,Ψ], Eman A. E. El-Helw [1,Ψ,*], Eman A. El-Bordany [1], Eman A. Ghareeb[1]

[1] Chemistry Department, Faculty of Science, Ain Shams University, Cairo, 11566, Egypt

***E-mail*: eman.abdelrahman@sci.asu.edu.eg**

Ψ: The first and second authors are equally contributed to this work.

Spectral data:

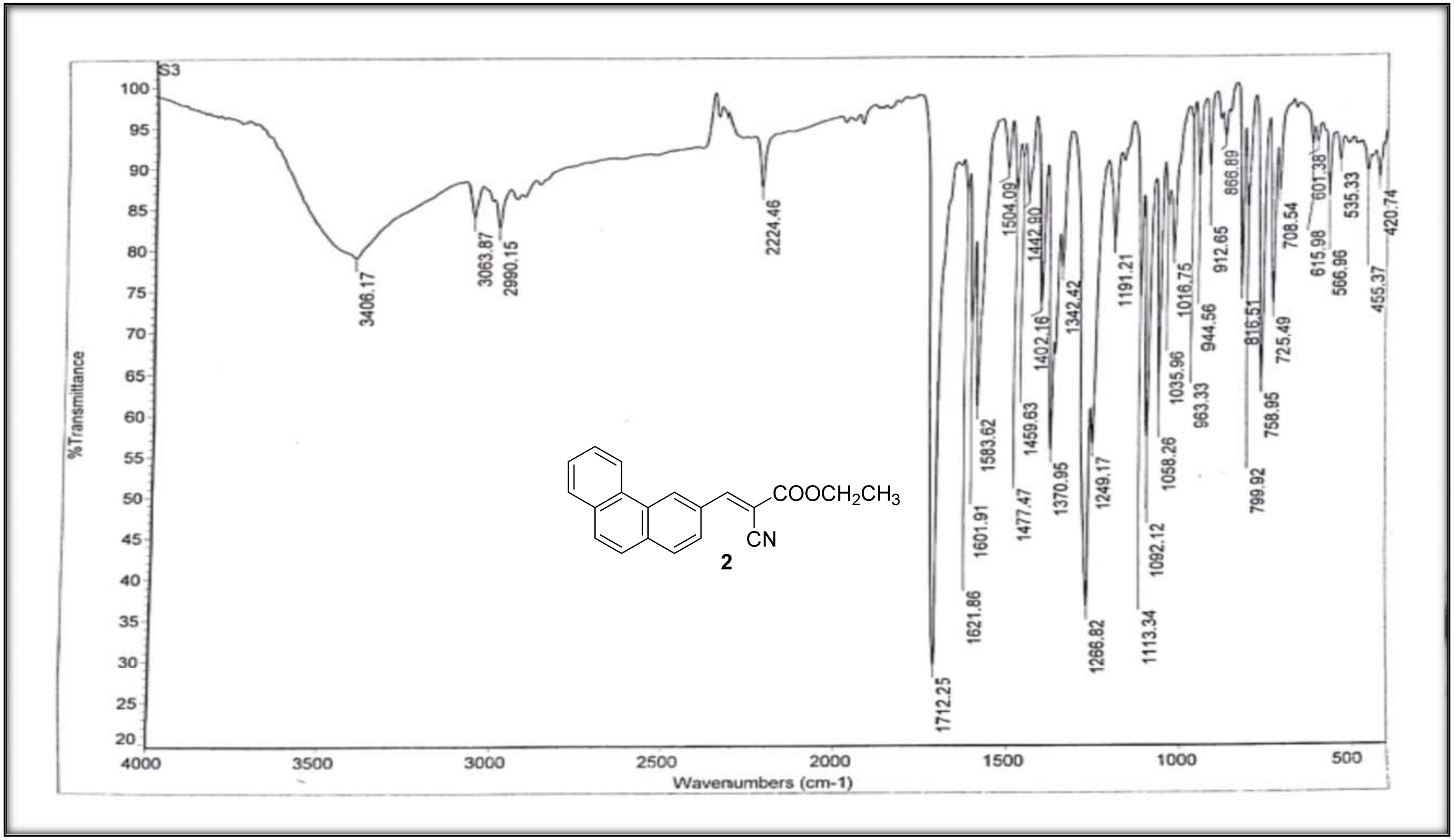

Fig. SD1. IR spectrum of compound **2**

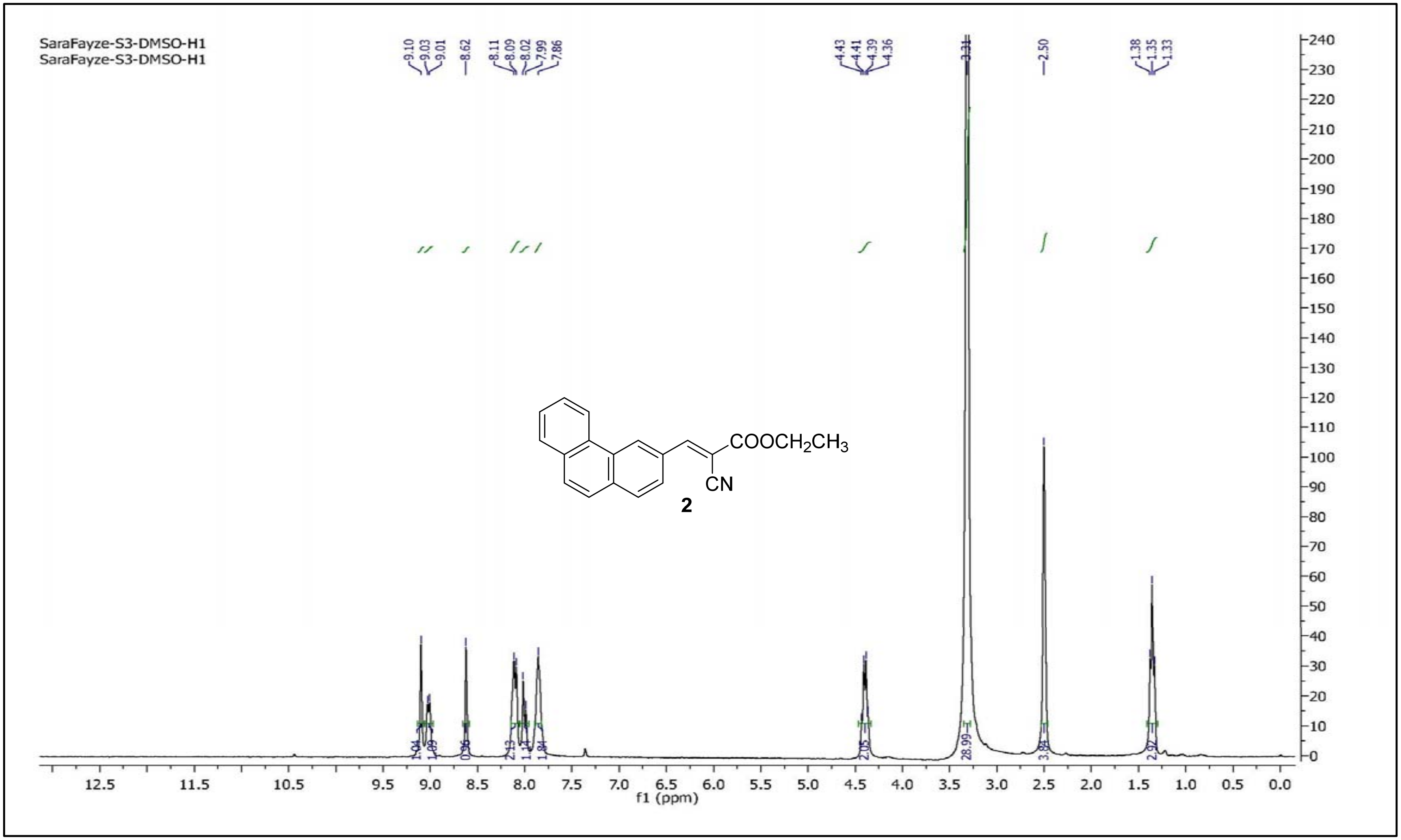

Fig. SD2. ^{1}H NMR spectrum (DMSO-d_6) of compound **2**

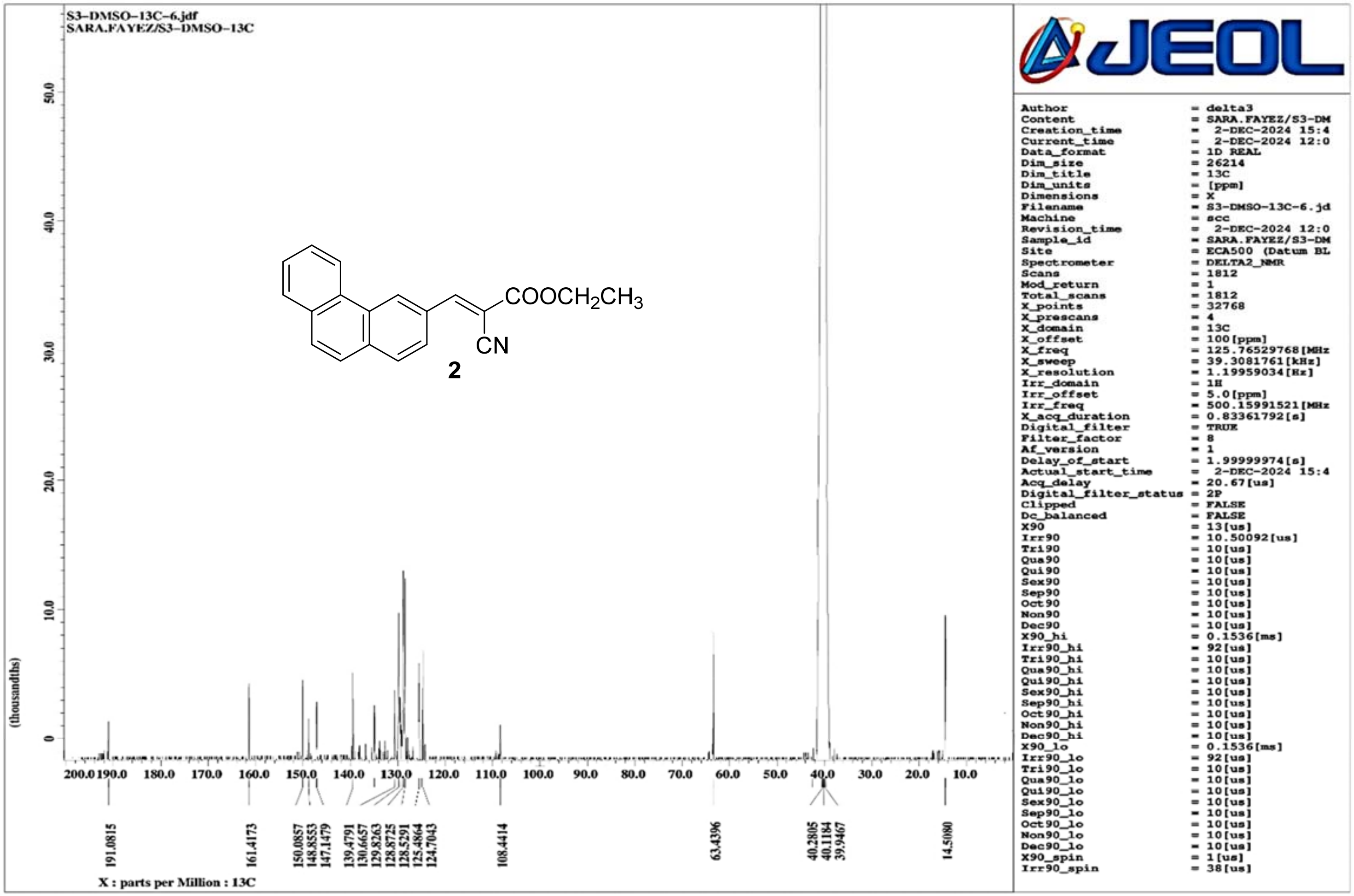

Cont. Fig. SD2. ^{13}C NMR spectrum (DMSO-d_6) of compound **2**

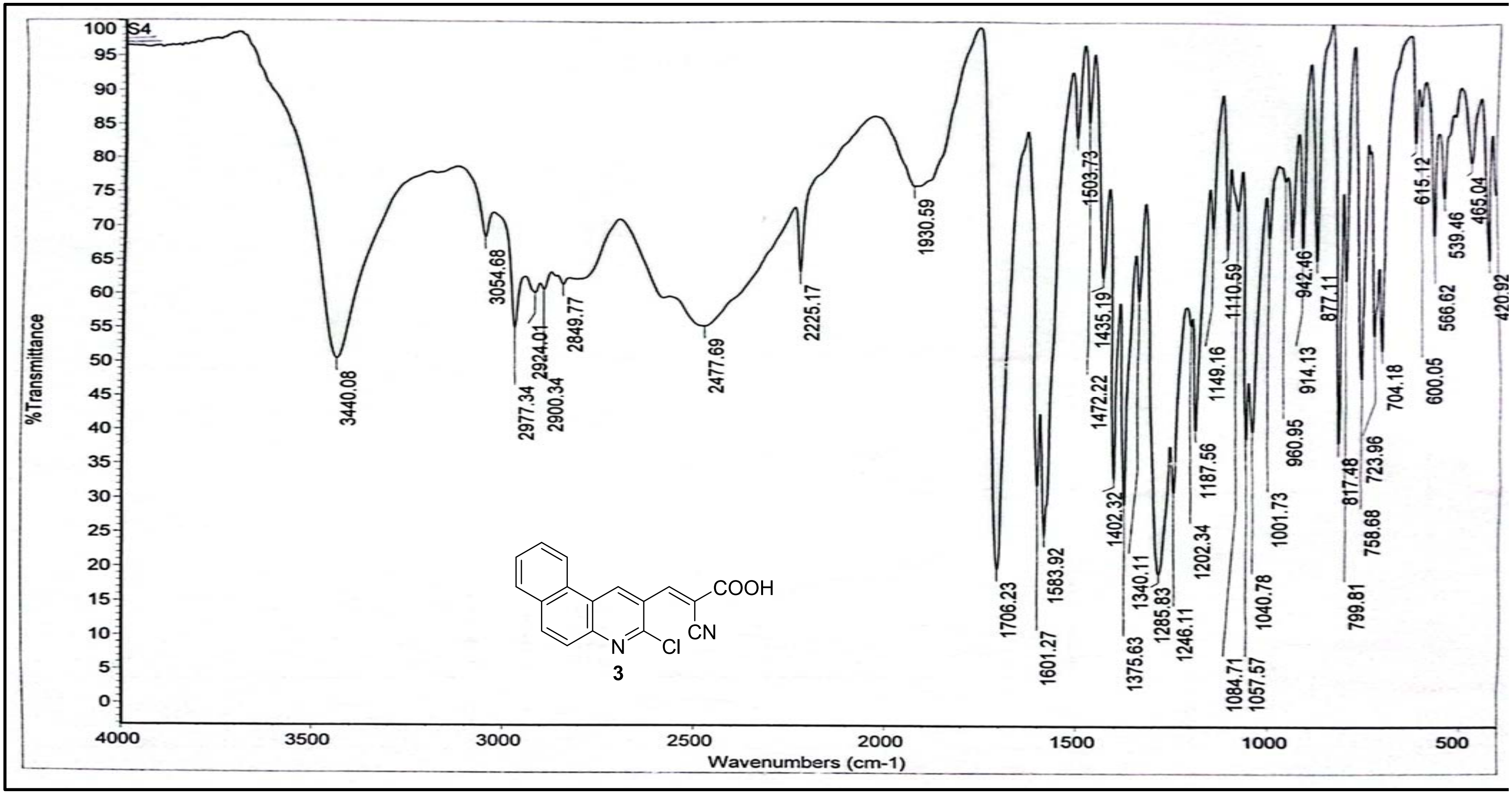

Fig. SD3. IR spectrum of compound **3**

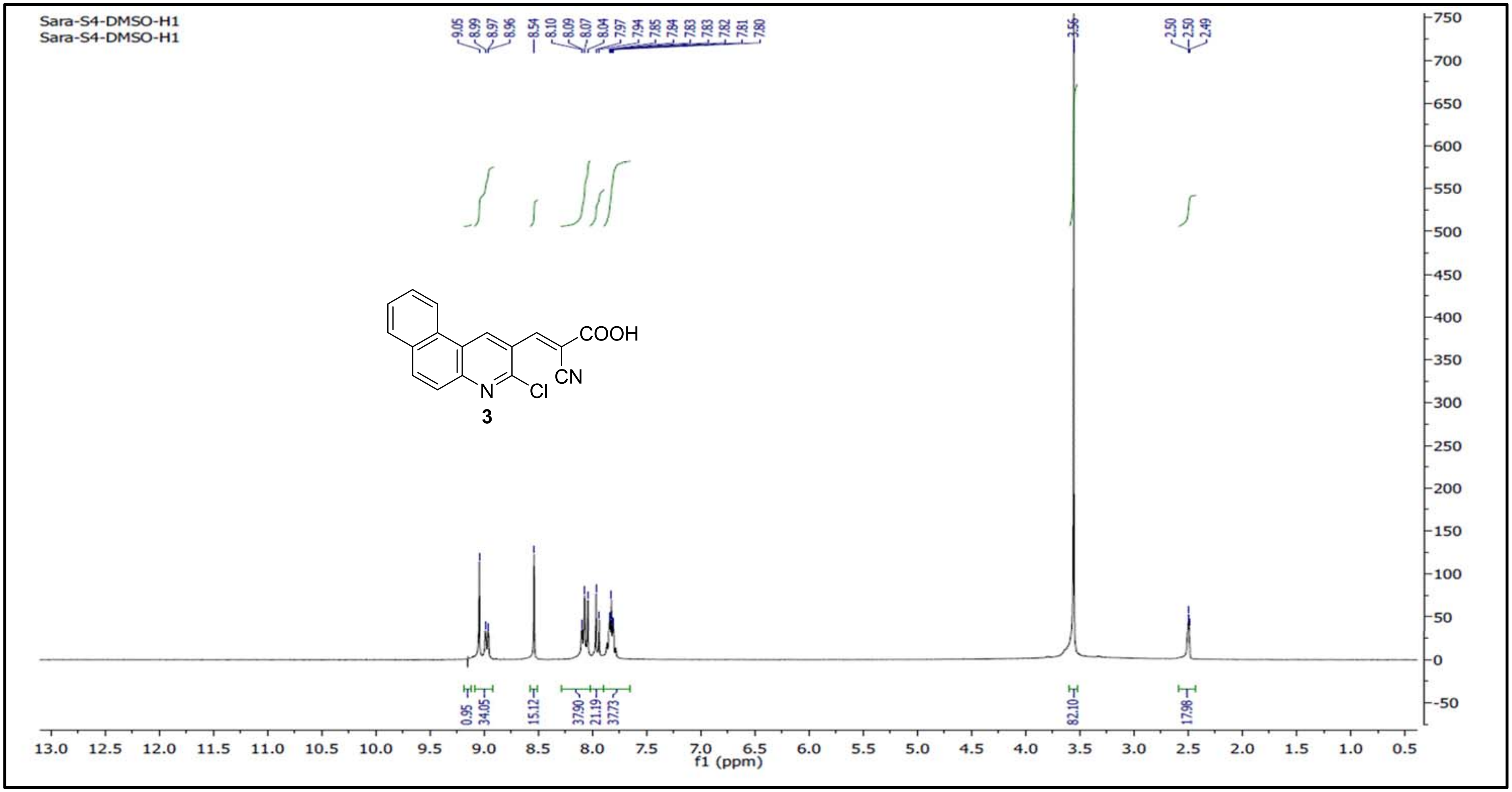

Fig. SD4. ^{1}H NMR spectrum (DMSO-d_6) of compound **3**

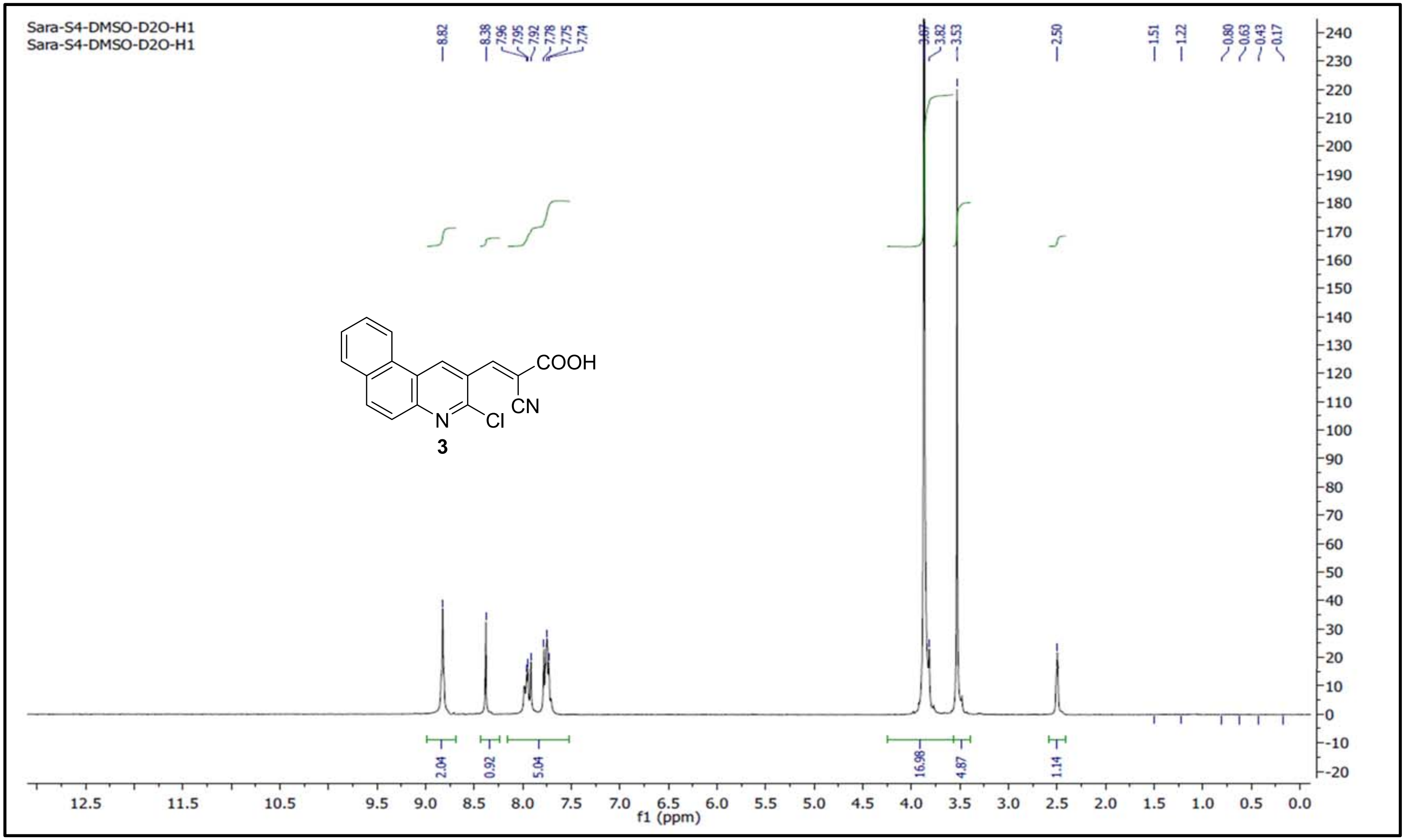

Fig. SD5. ^{1}H NMR spectrum (DMSO-d_6+D_2O) of compound **3**

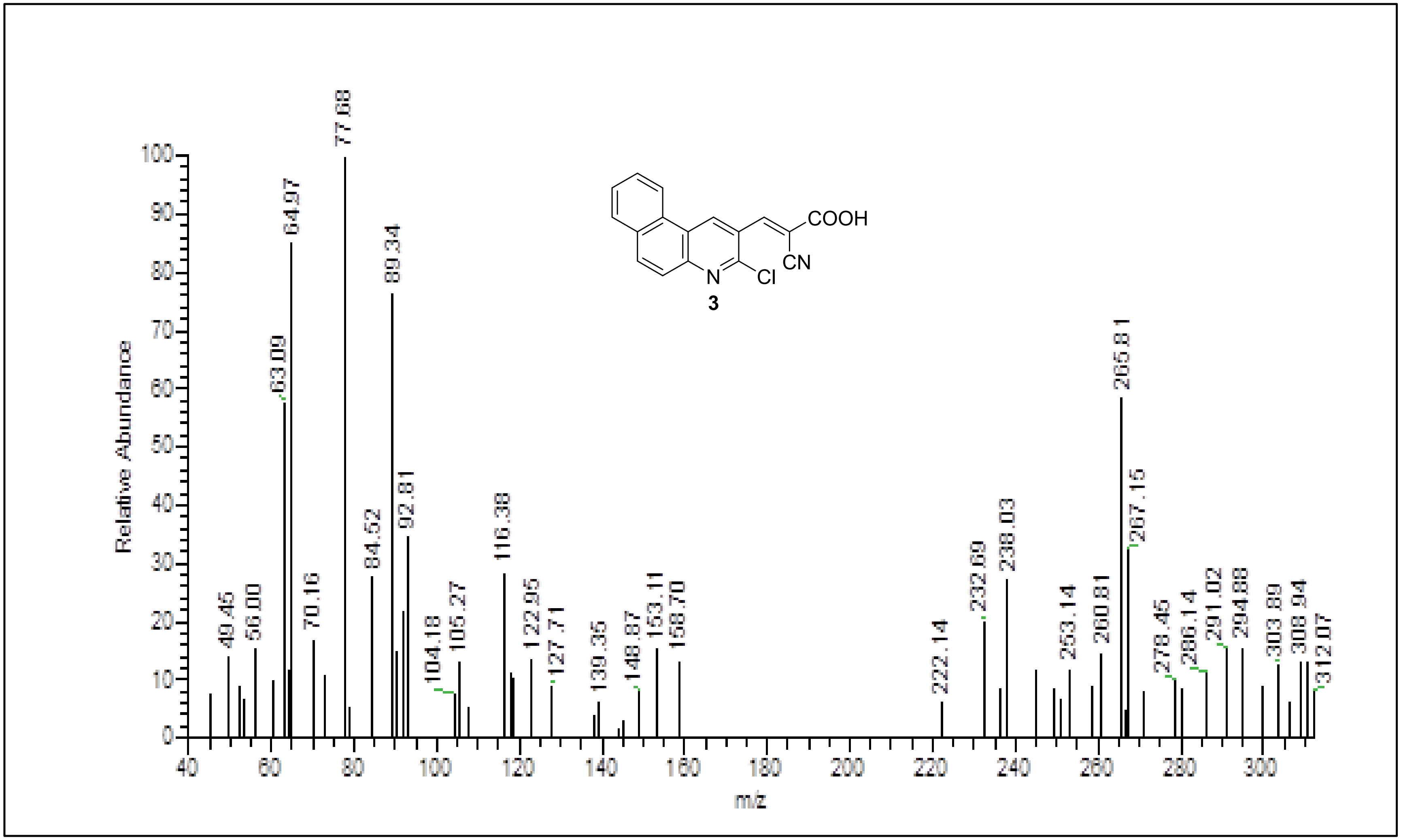

Fig. SD6. Mass spectrum of compound **3**

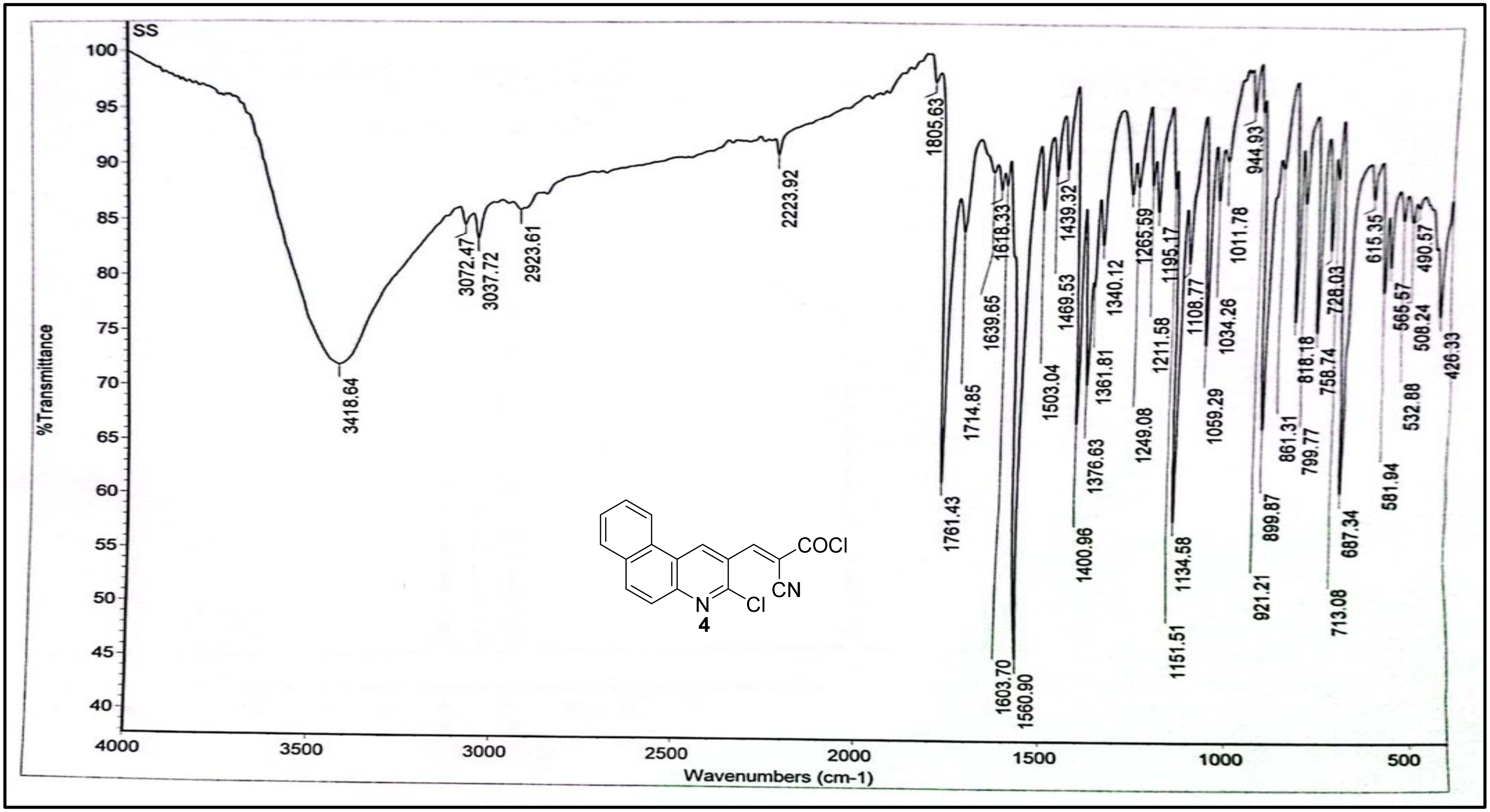

Fig. SD7. IR spectrum of compound **4**

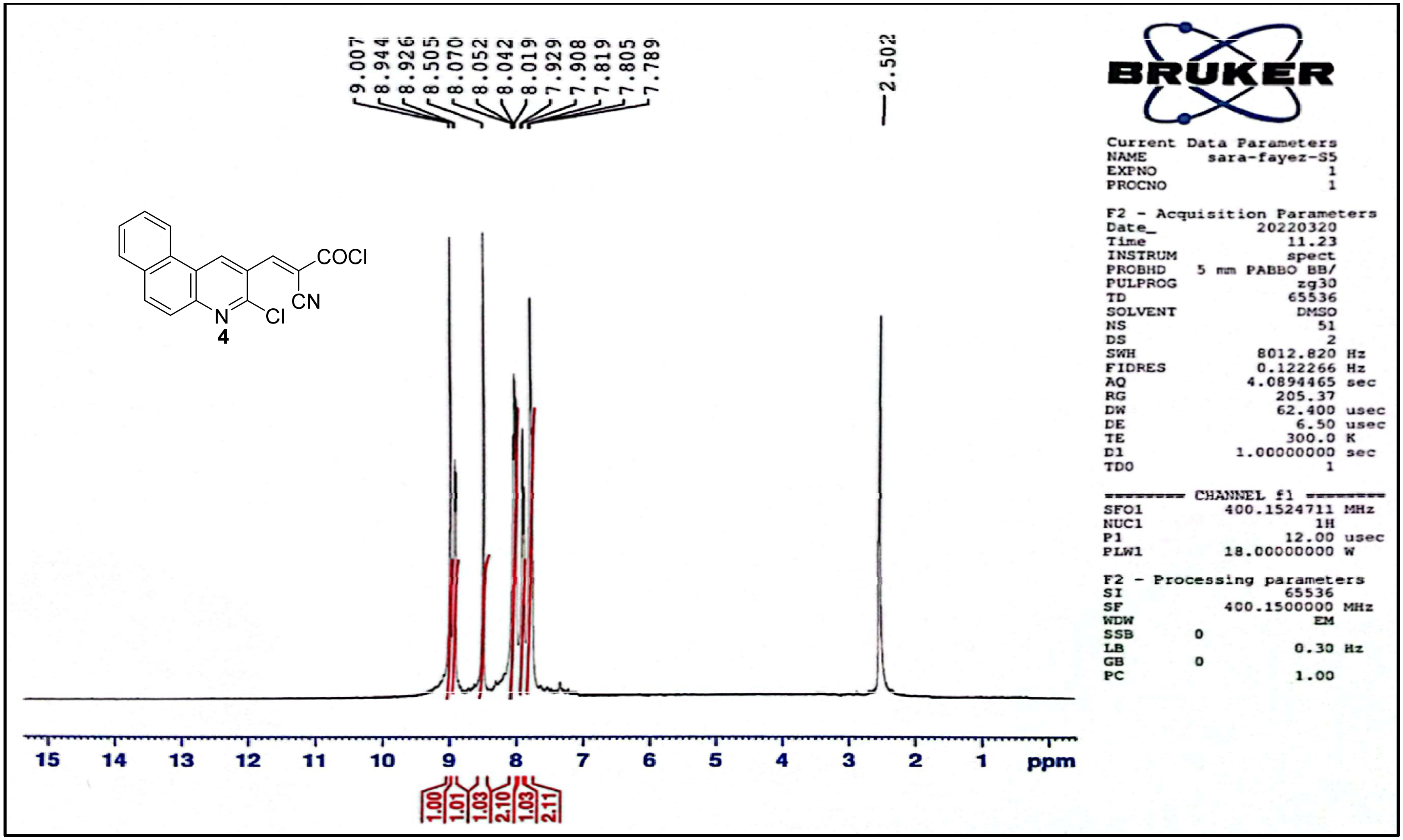

Fig. SD8. ^{1}H NMR spectrum (DMSO-d_6) of compound **4**

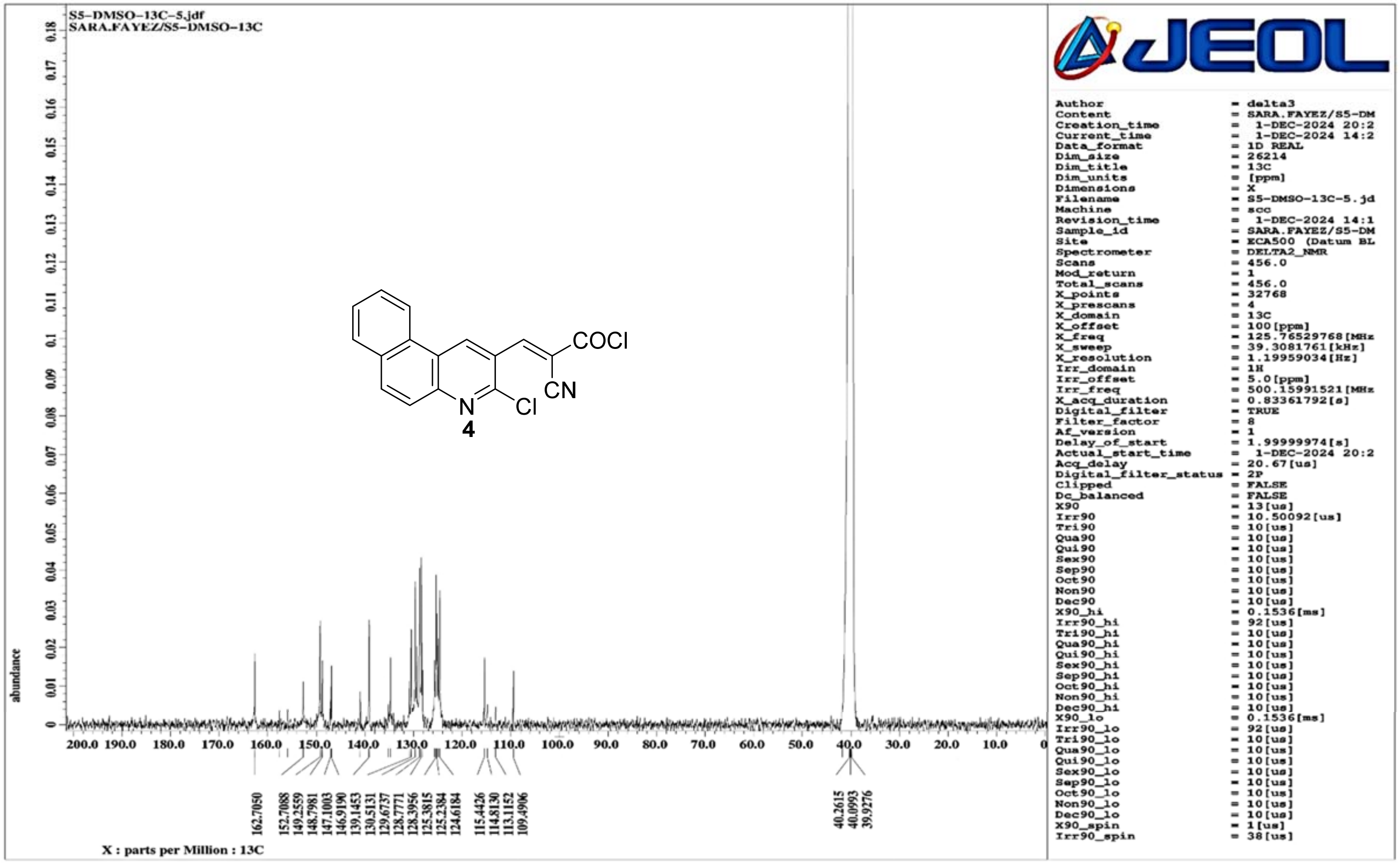

Cont. Fig. SD8. ^{13}C NMR spectrum (DMSO-d_6) of compound **4**

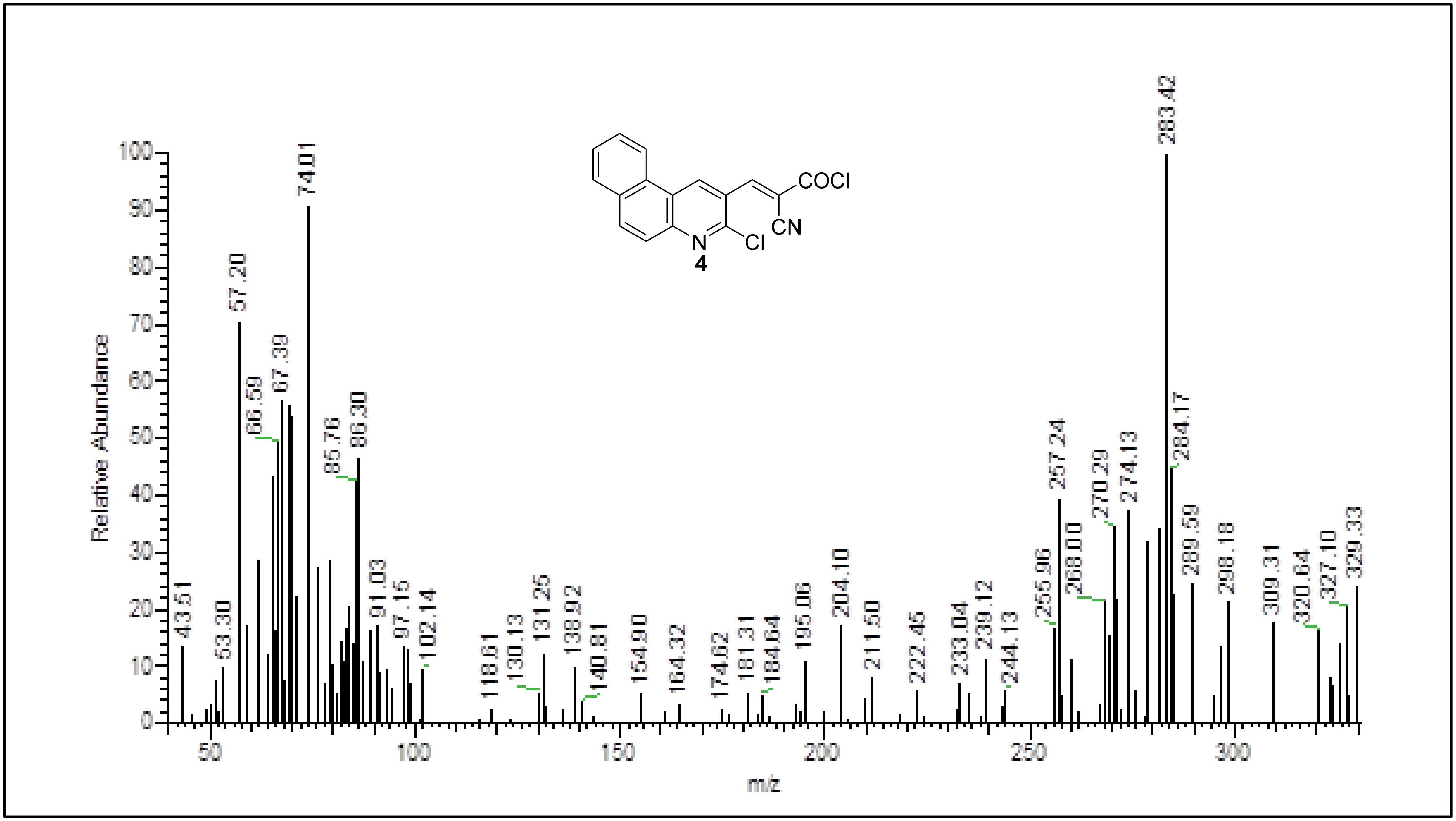

Fig. SD9. Mass spectrum of compound **4**

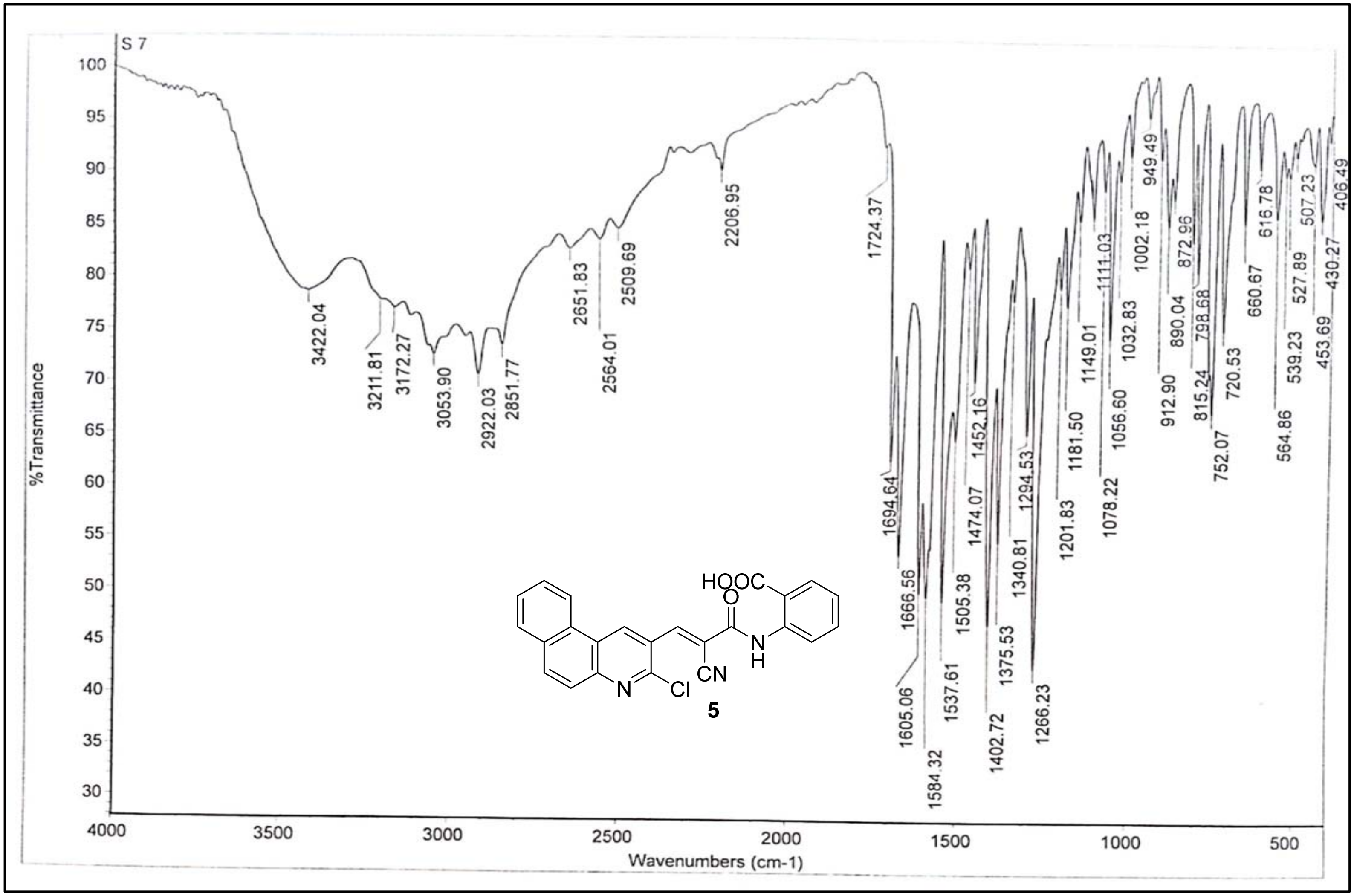

Fig. SD10. IR spectrum of compound **5**

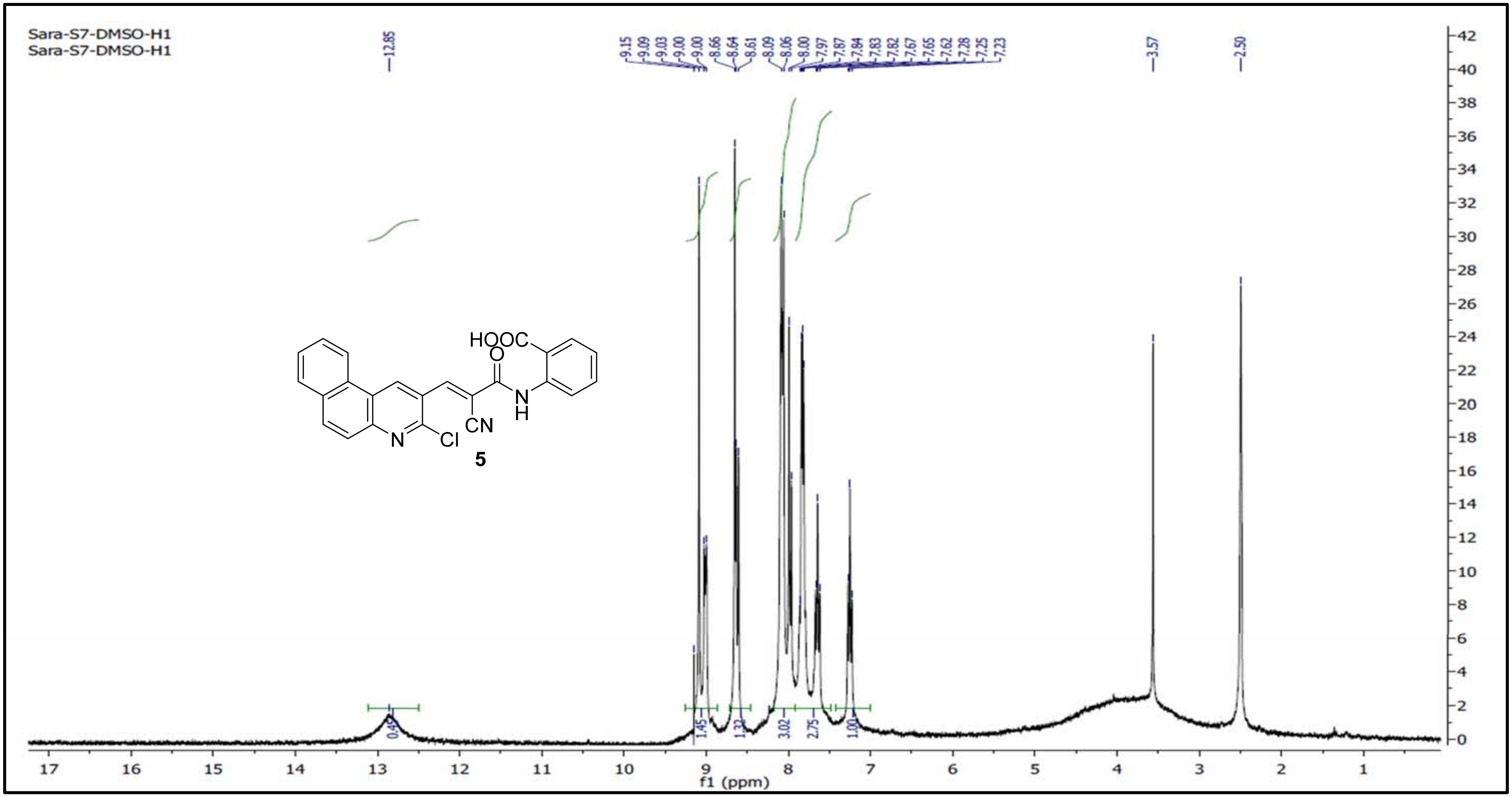

Fig. SD11. ^{1}H NMR spectrum (DMSO-d_6) of compound **5**

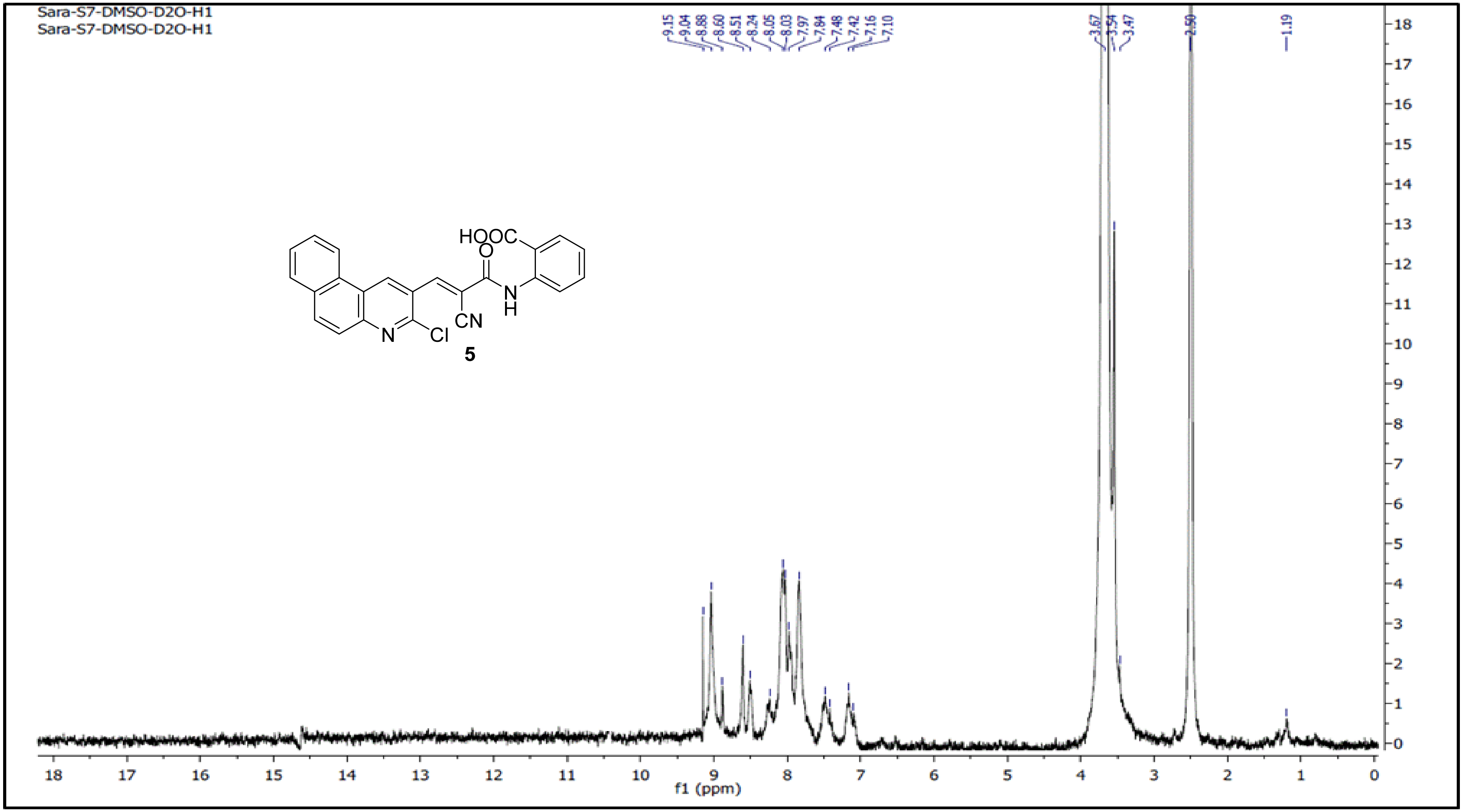

Fig. SD12. ^{1}H NMR spectrum (DMSO-d_6+D_2O) of compound **5**

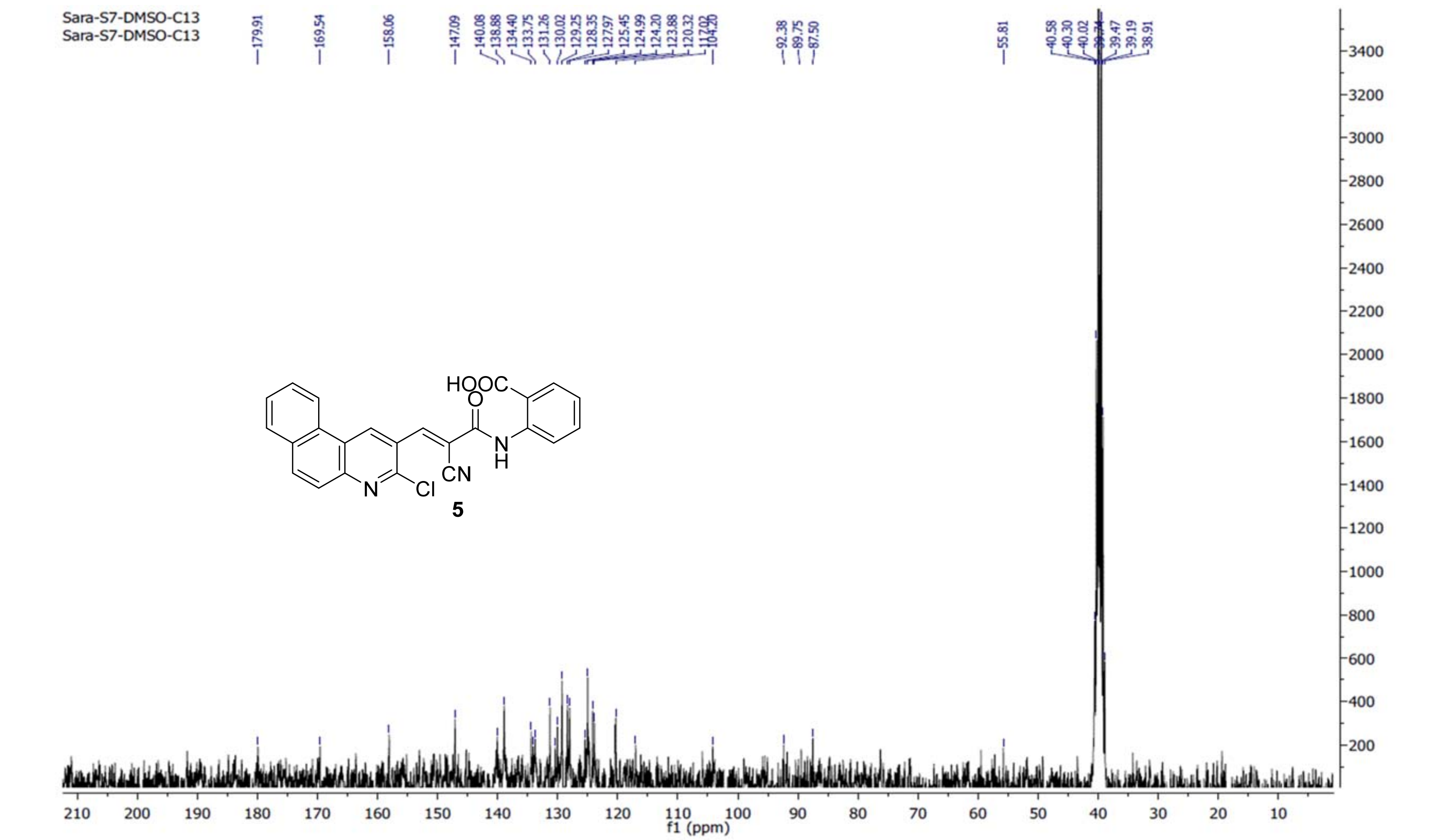

Fig. SD13. ^{13}C NMR spectrum (DMSO-d_6) of compound **5**

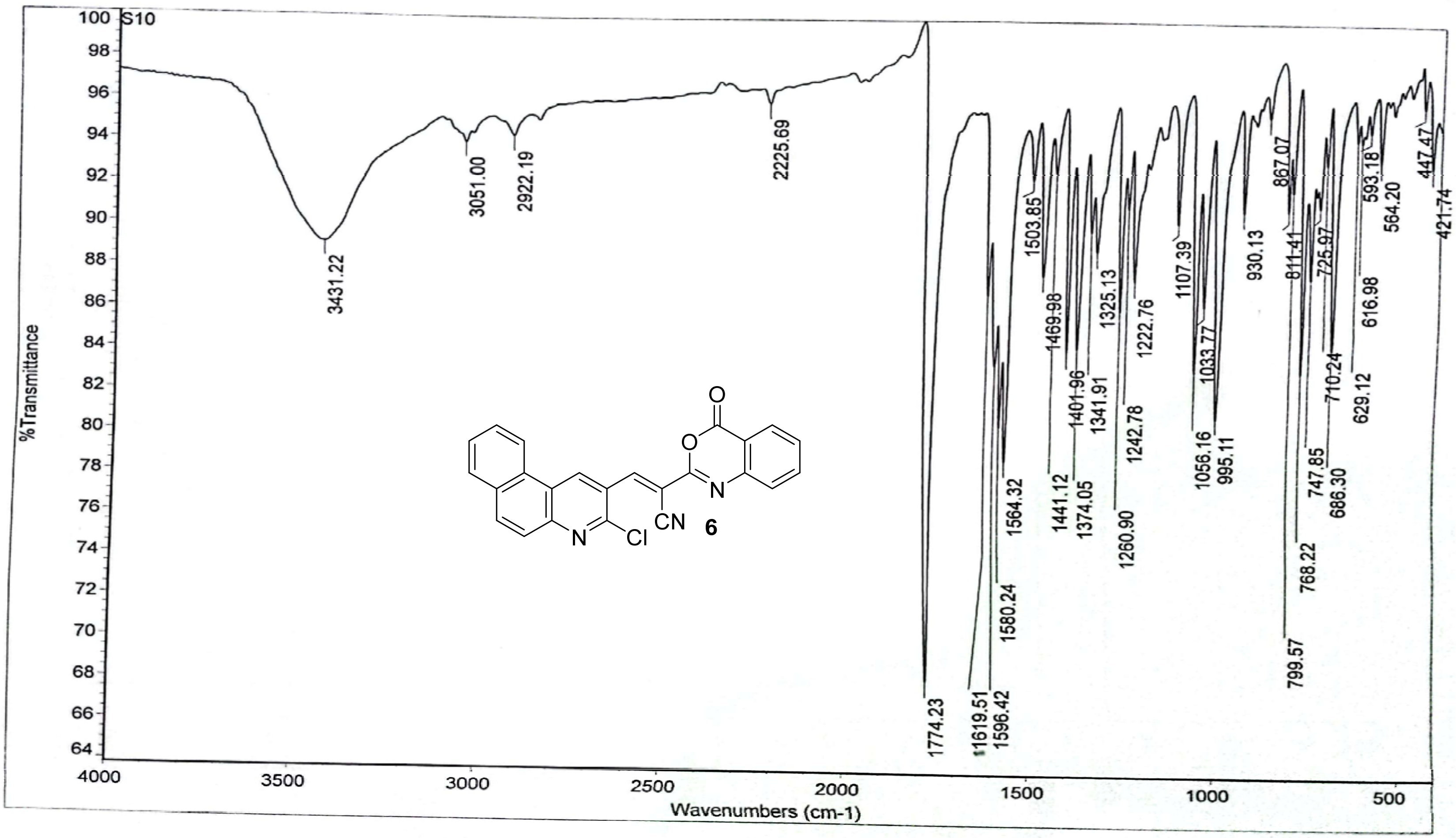

Fig. SD14. IR spectrum of compound **6**

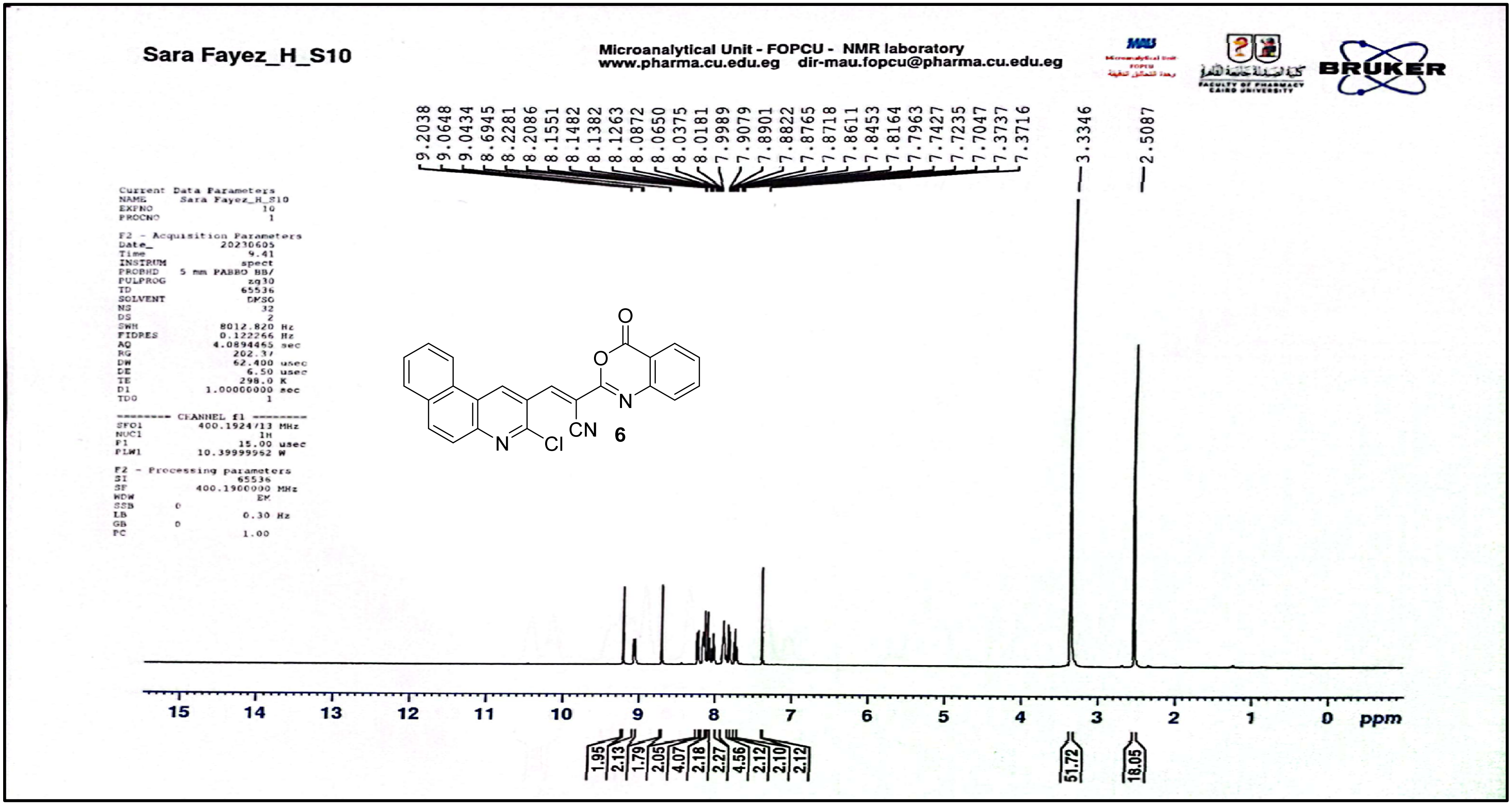

Fig. SD15. ^{1}H NMR spectrum (DMSO-d_6) of compound **6**

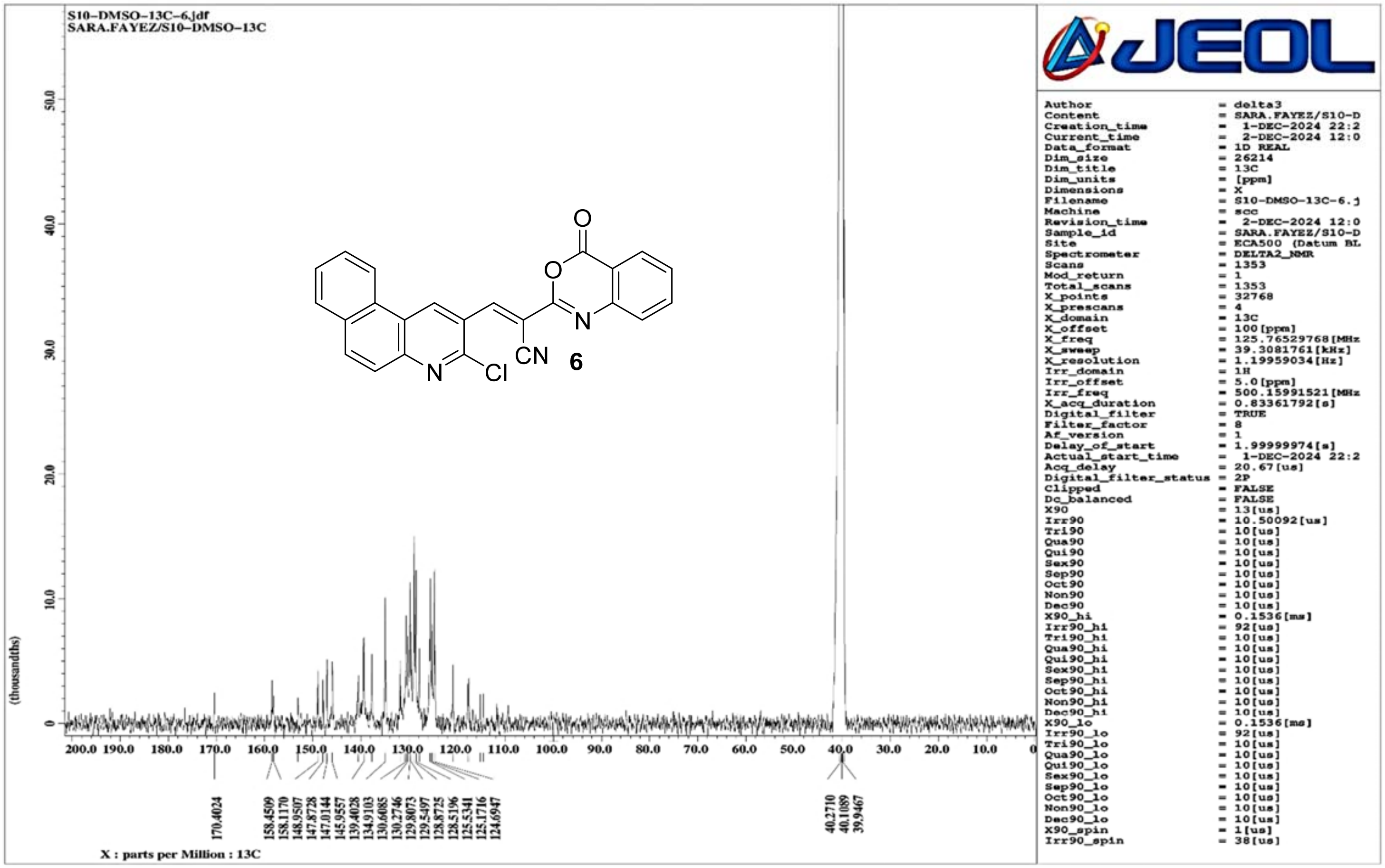

Cont. Fig. SD15. ^{13}C NMR spectrum (DMSO-d_6) of compound **6**

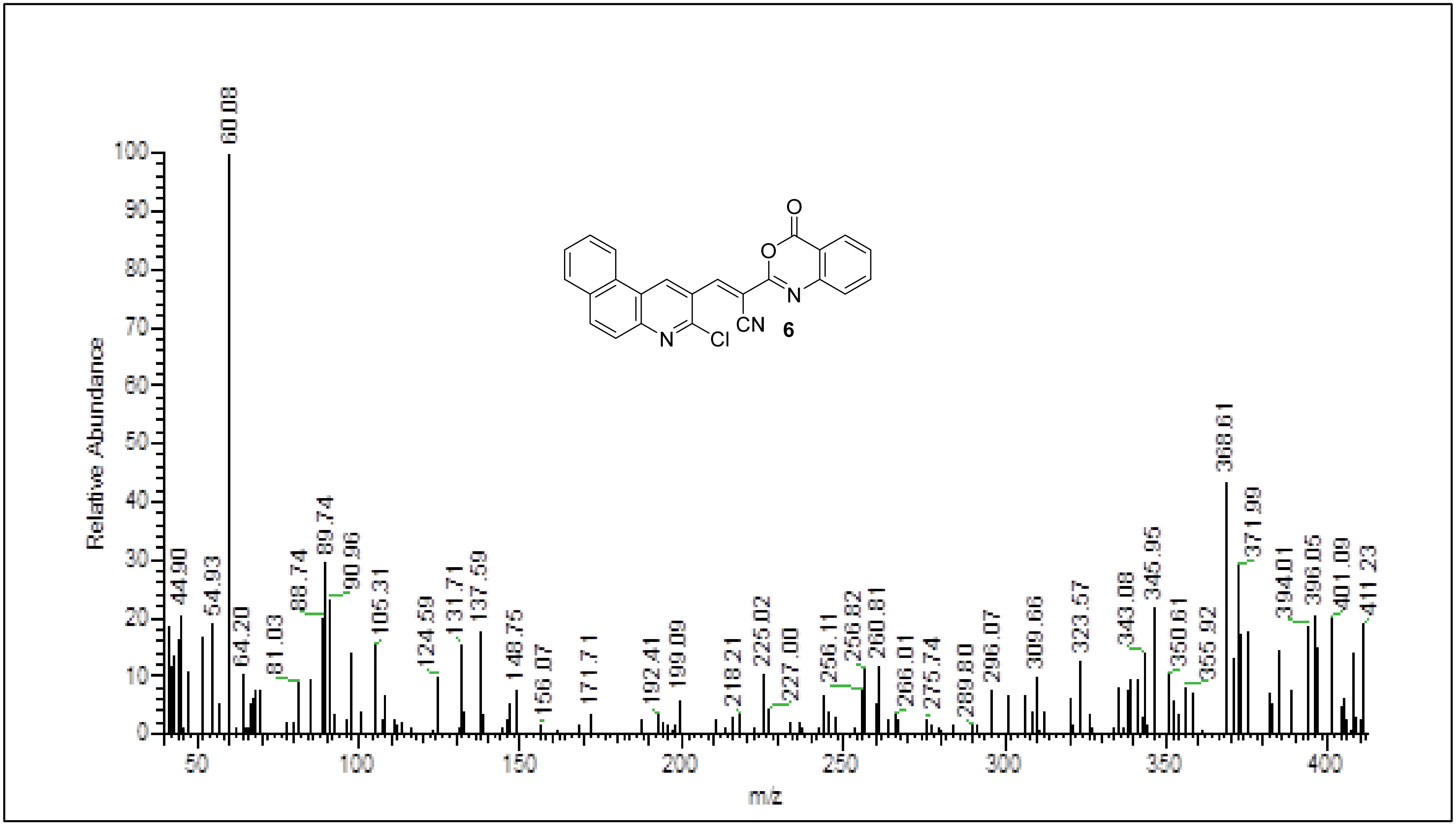

Fig. SD16. Mass spectrum of compound **6**

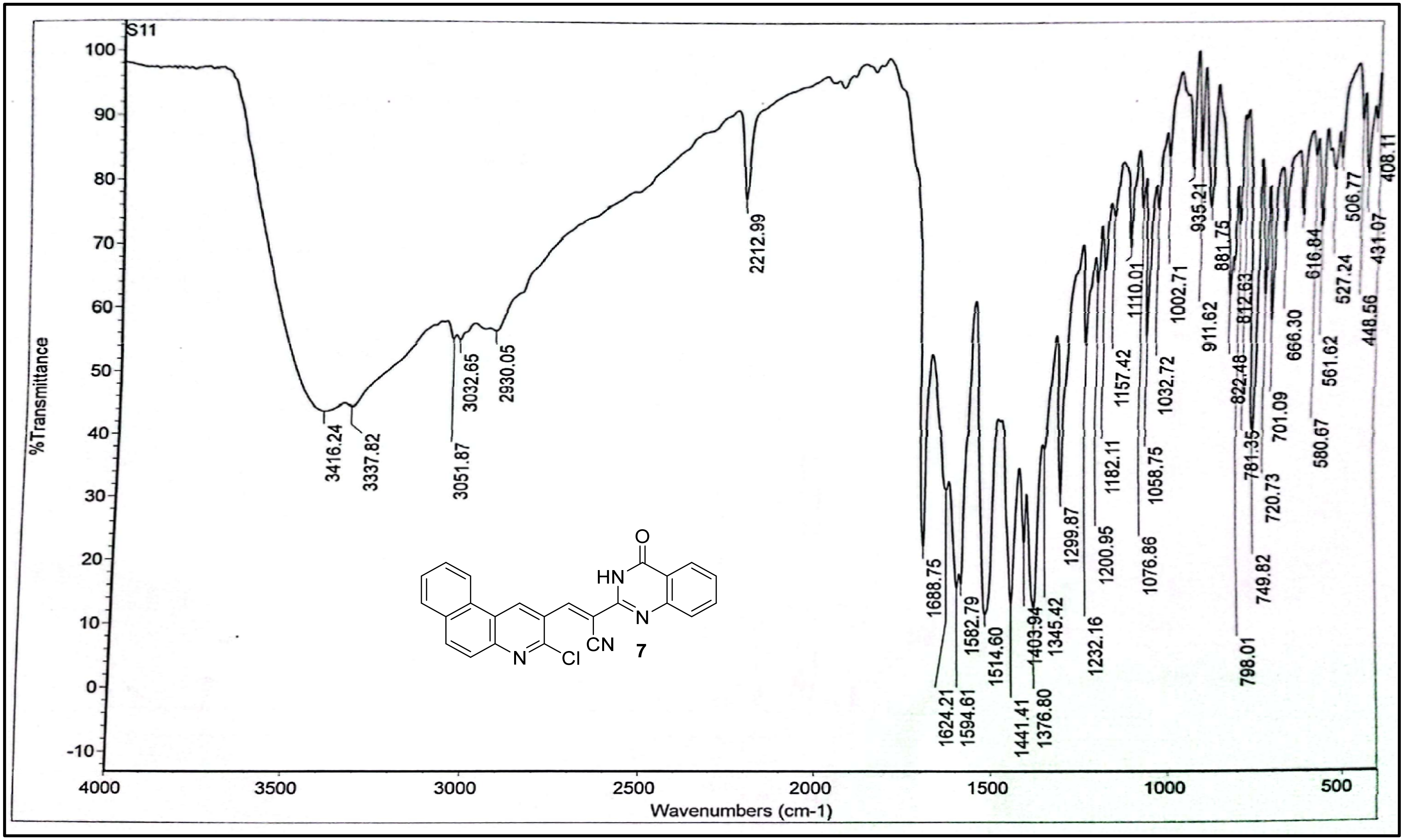

Fig. SD17. IR spectrum of compound **7**

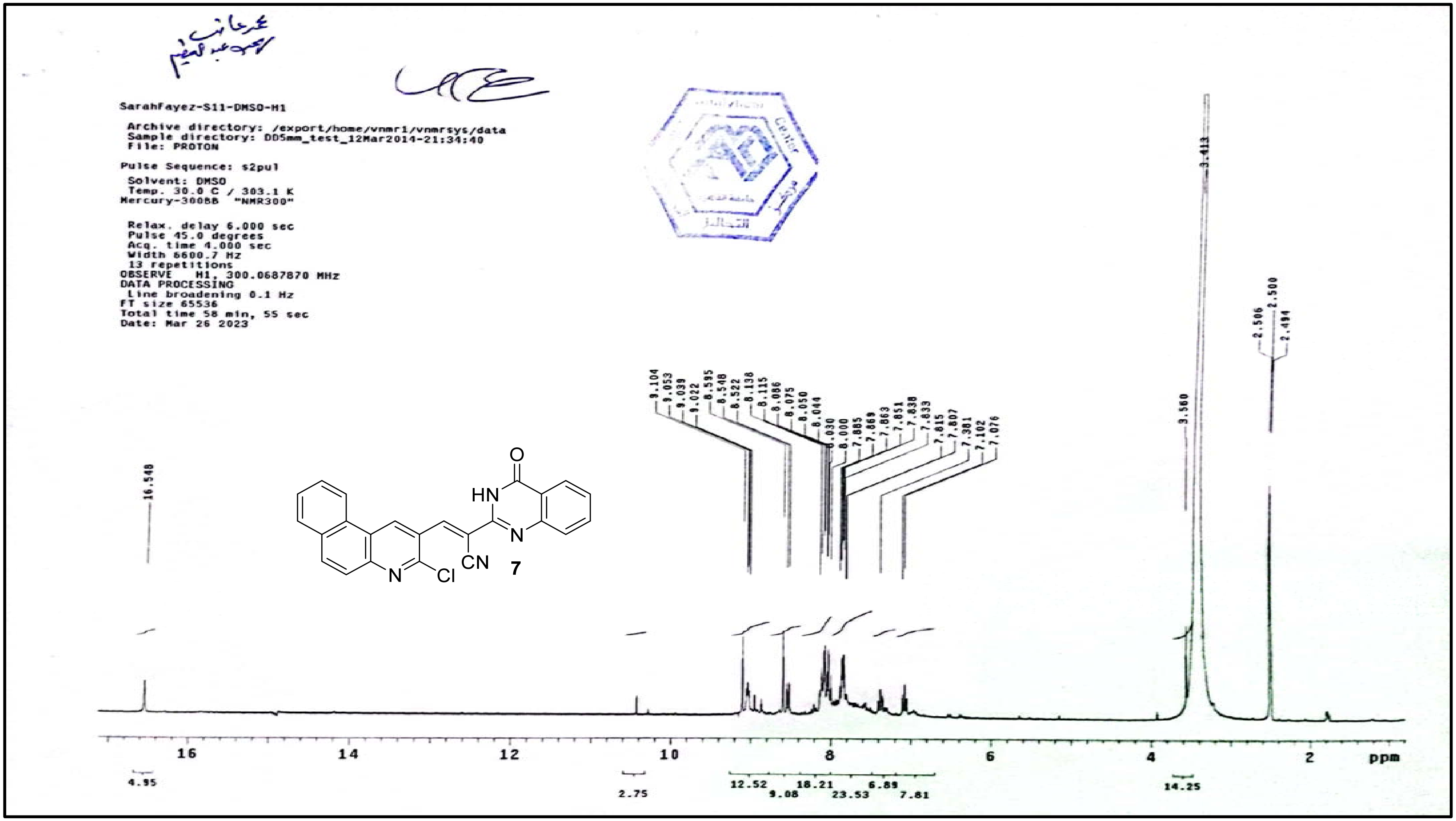

Fig. SD18. ^{1}H NMR spectrum (DMSO-d_6) of compound **7**

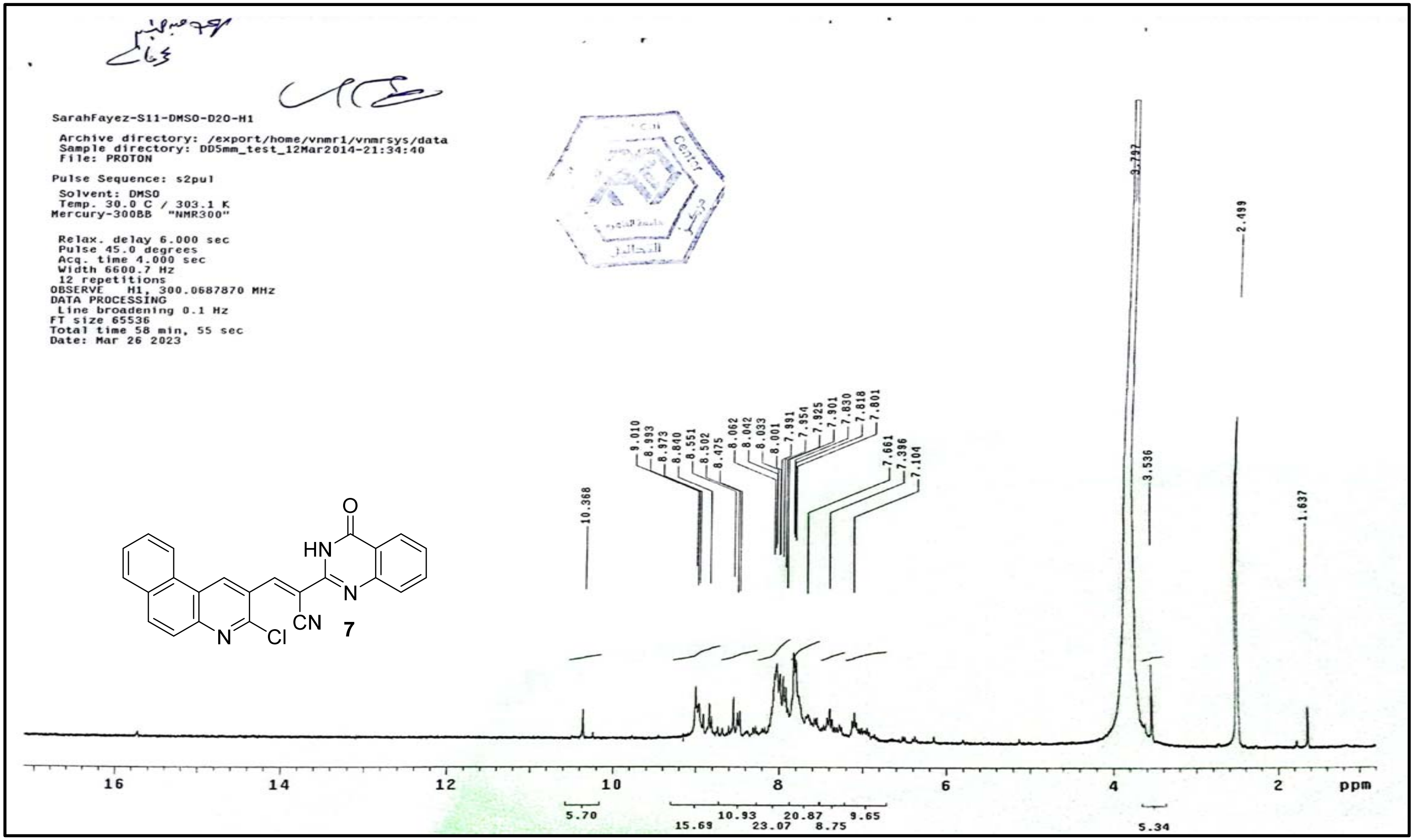

Fig. SD19. ^{1}H NMR spectrum (DMSO-d_6+D_2O) of compound **7**

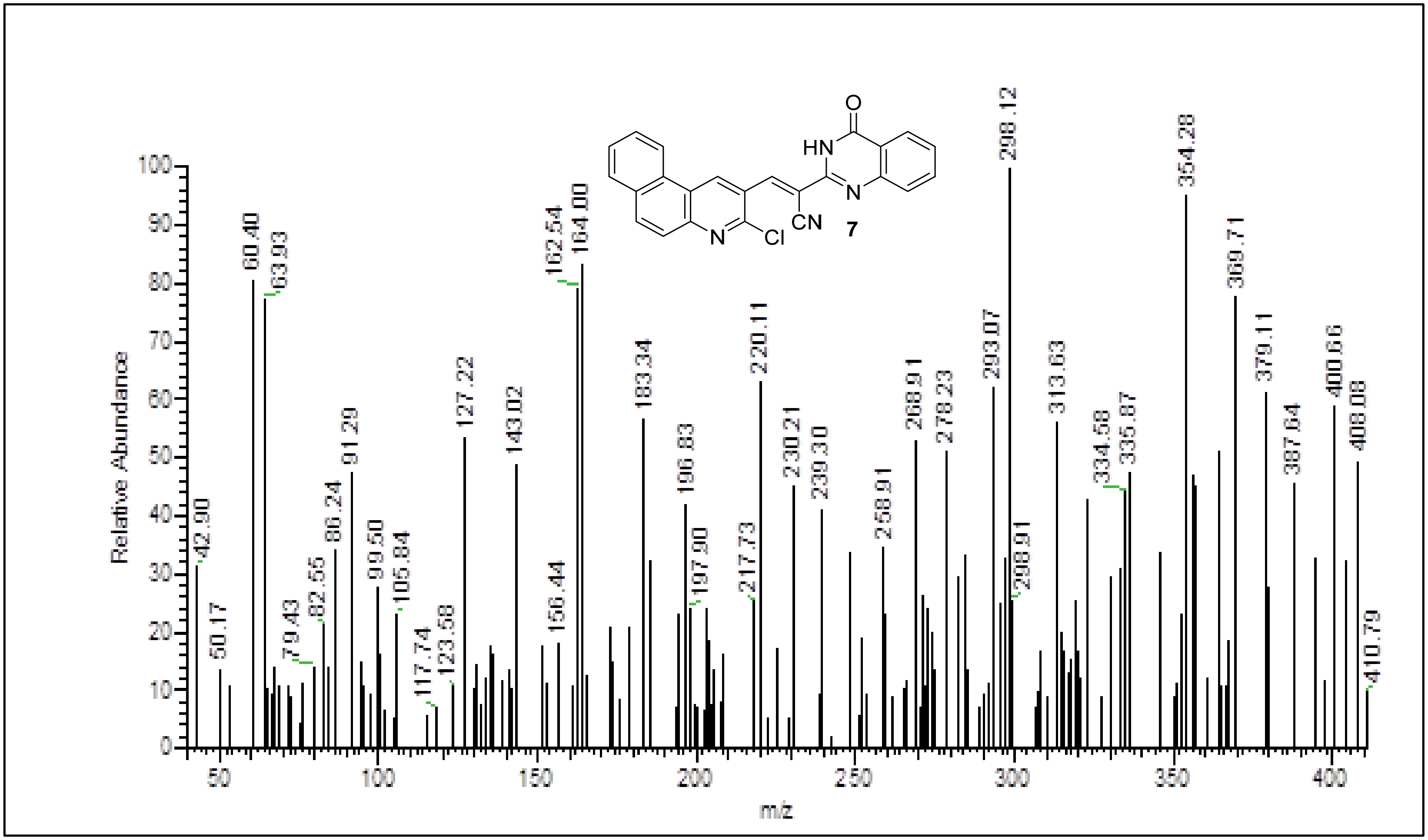

Fig. SD20. Mass spectrum of compound **7**

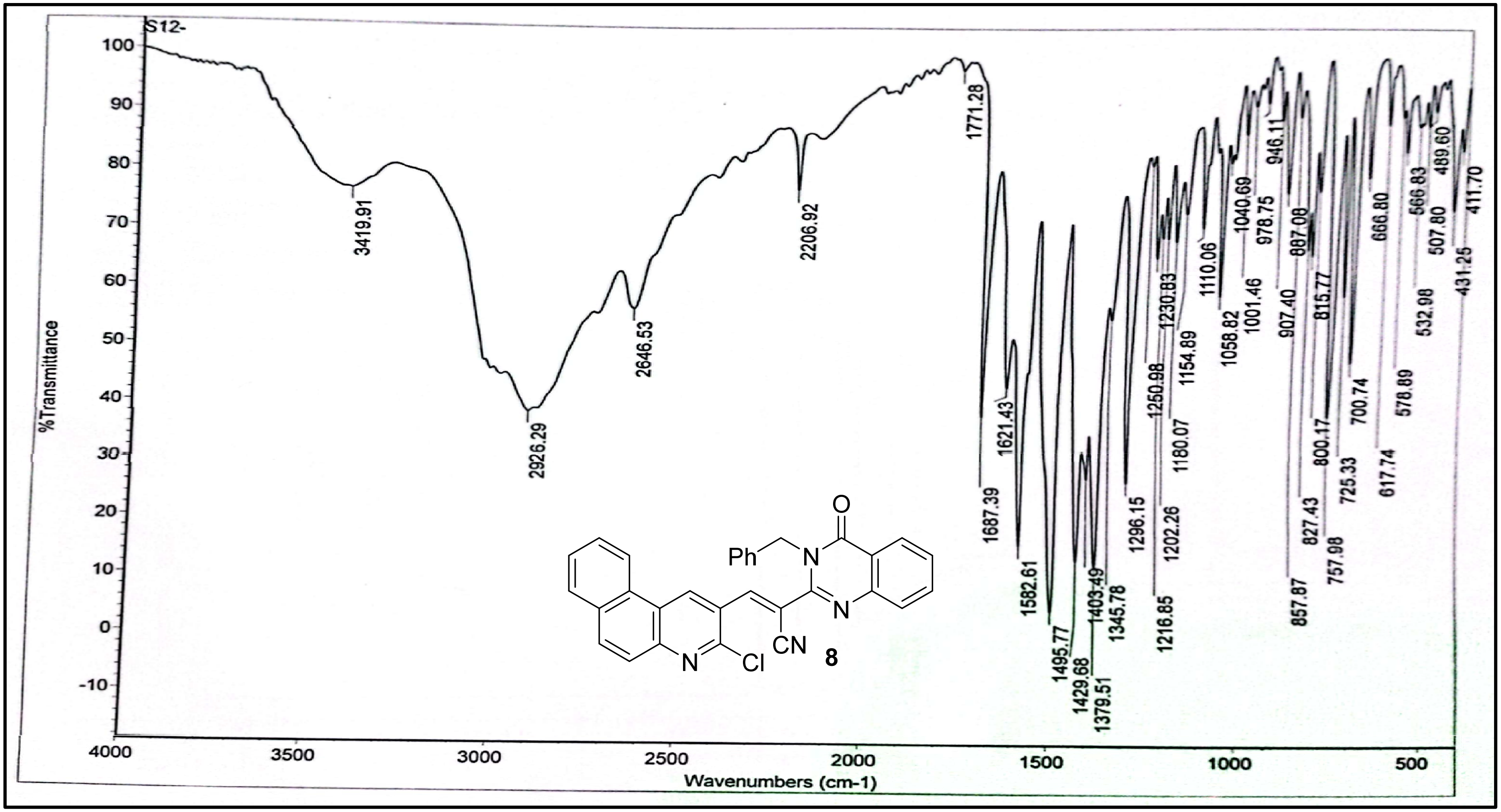

Fig. SD21. IR spectrum of compound **8**

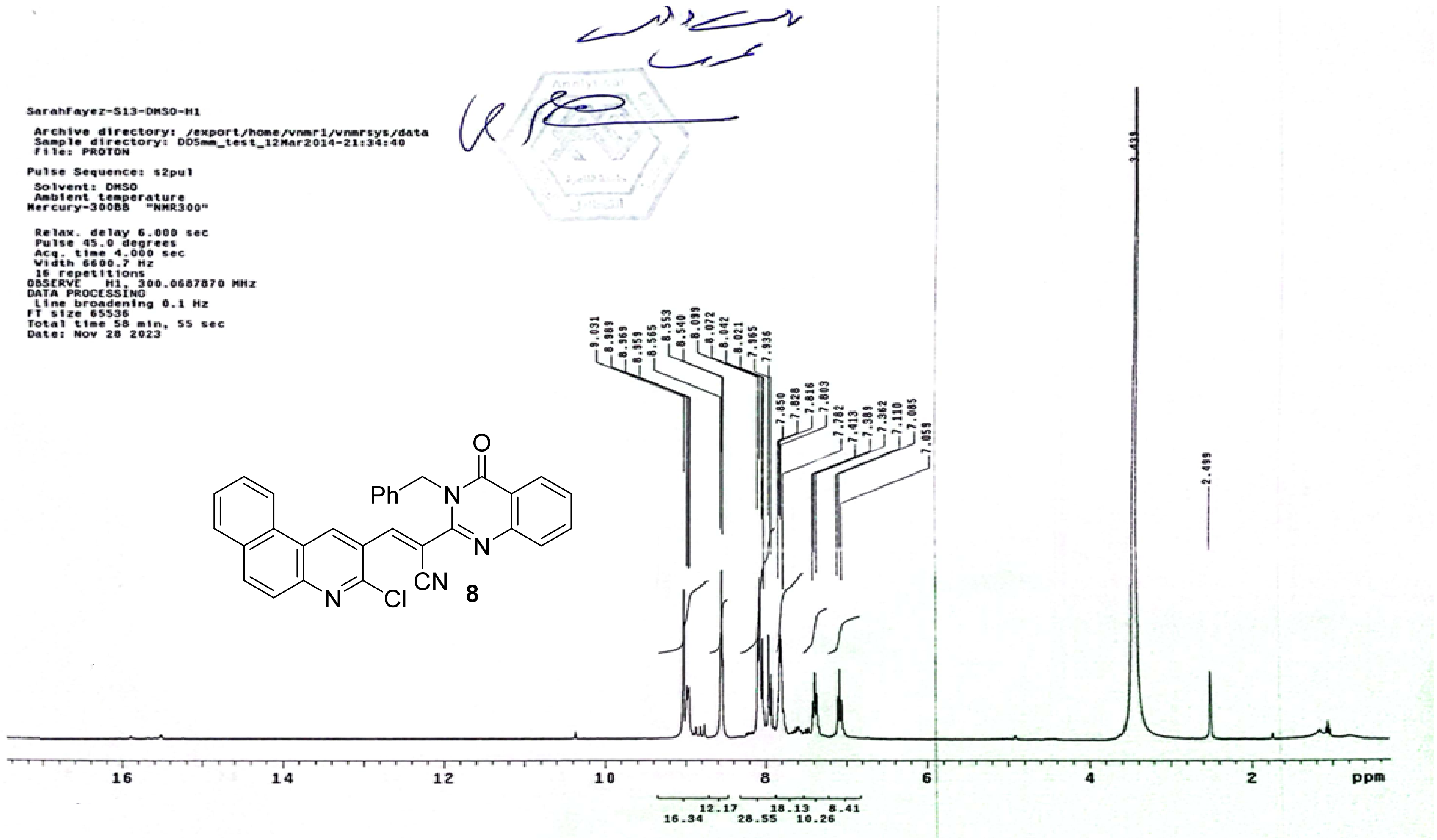

Fig. SD22. ^{1}H NMR spectrum (DMSO-d_6) of compound **8**

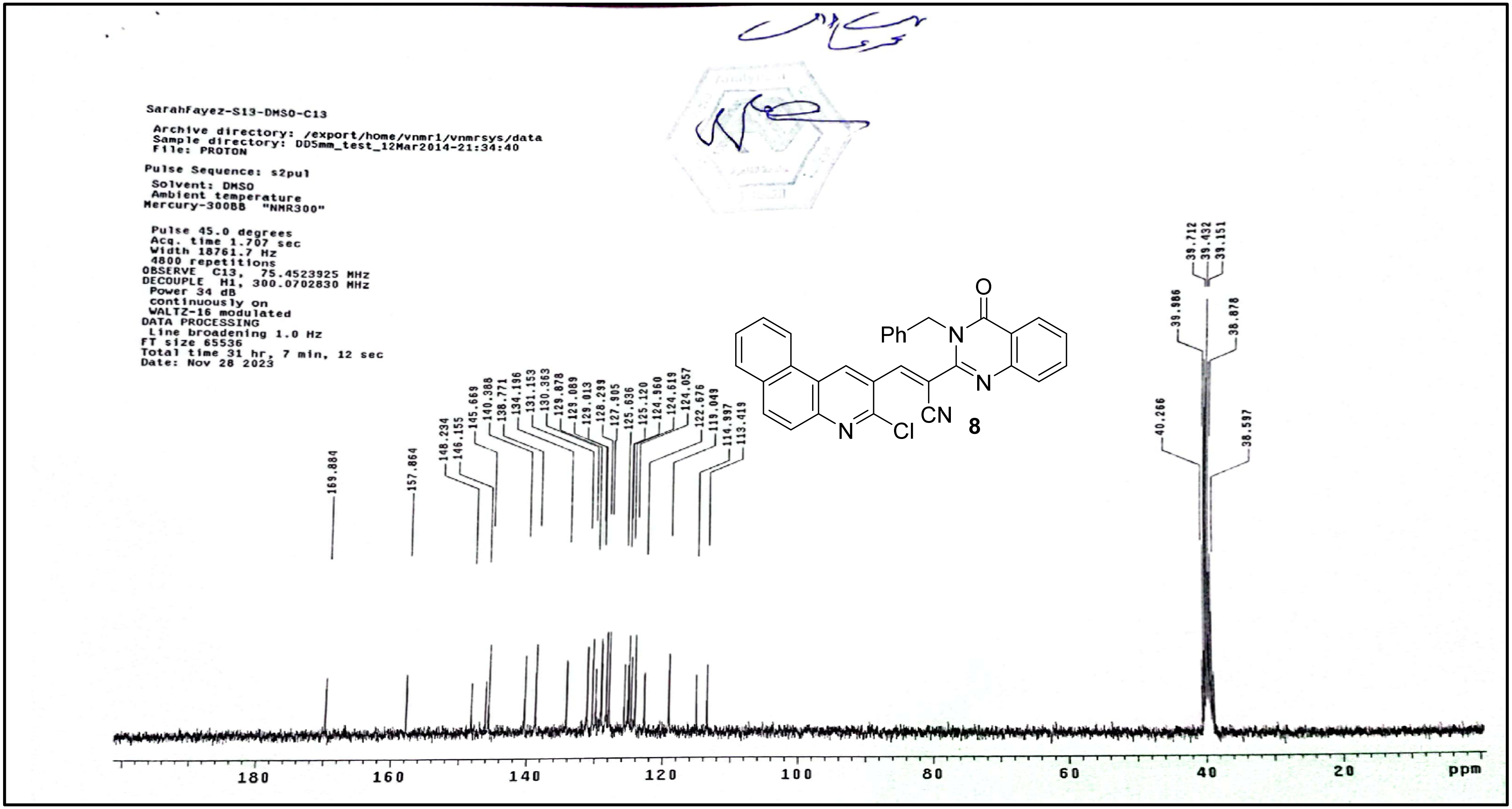

Fig. SD23. ^{13}C NMR spectrum (DMSO-d_6) of compound **8**

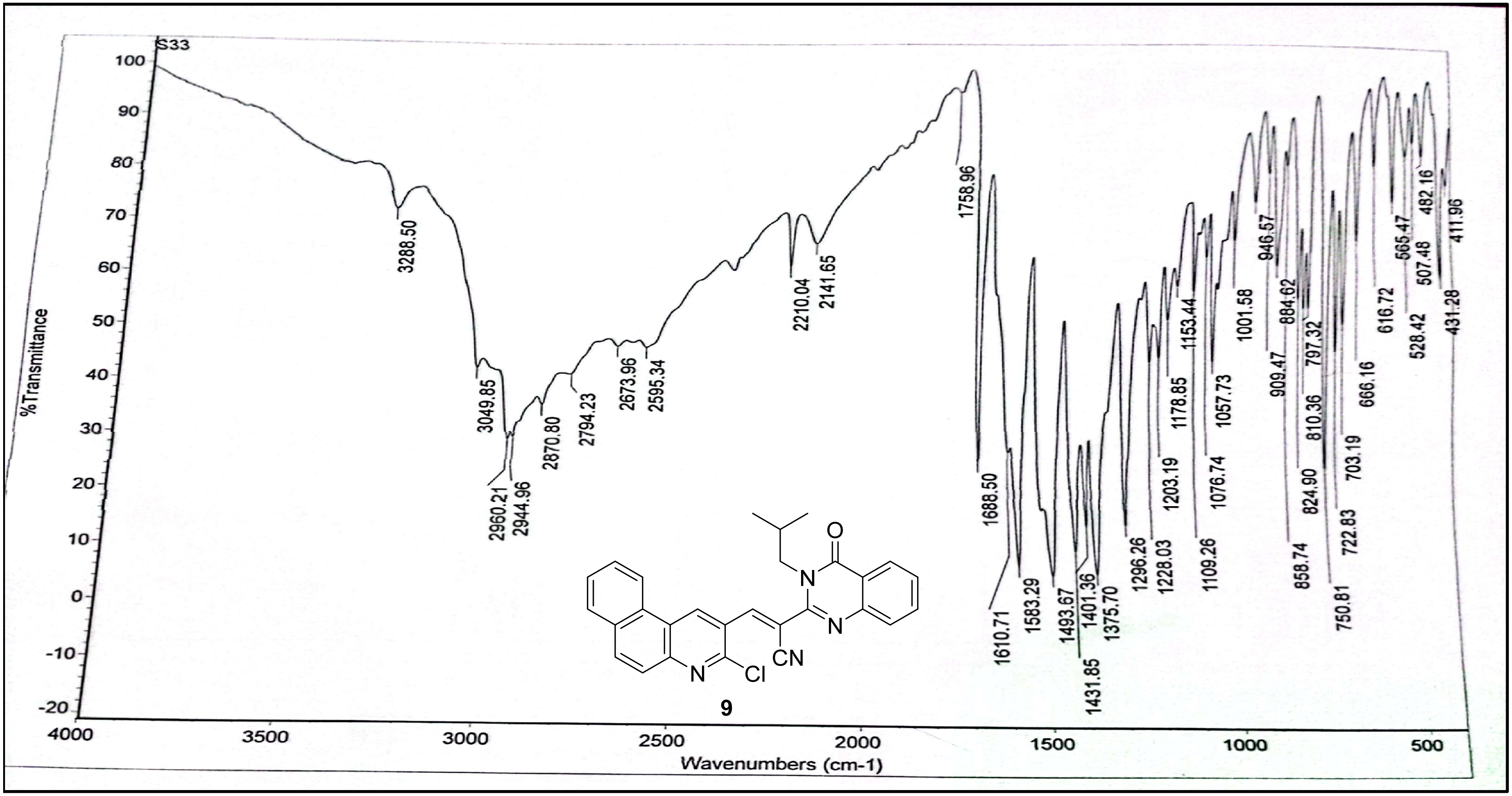

Fig. SD24. IR spectrum of compound **9**

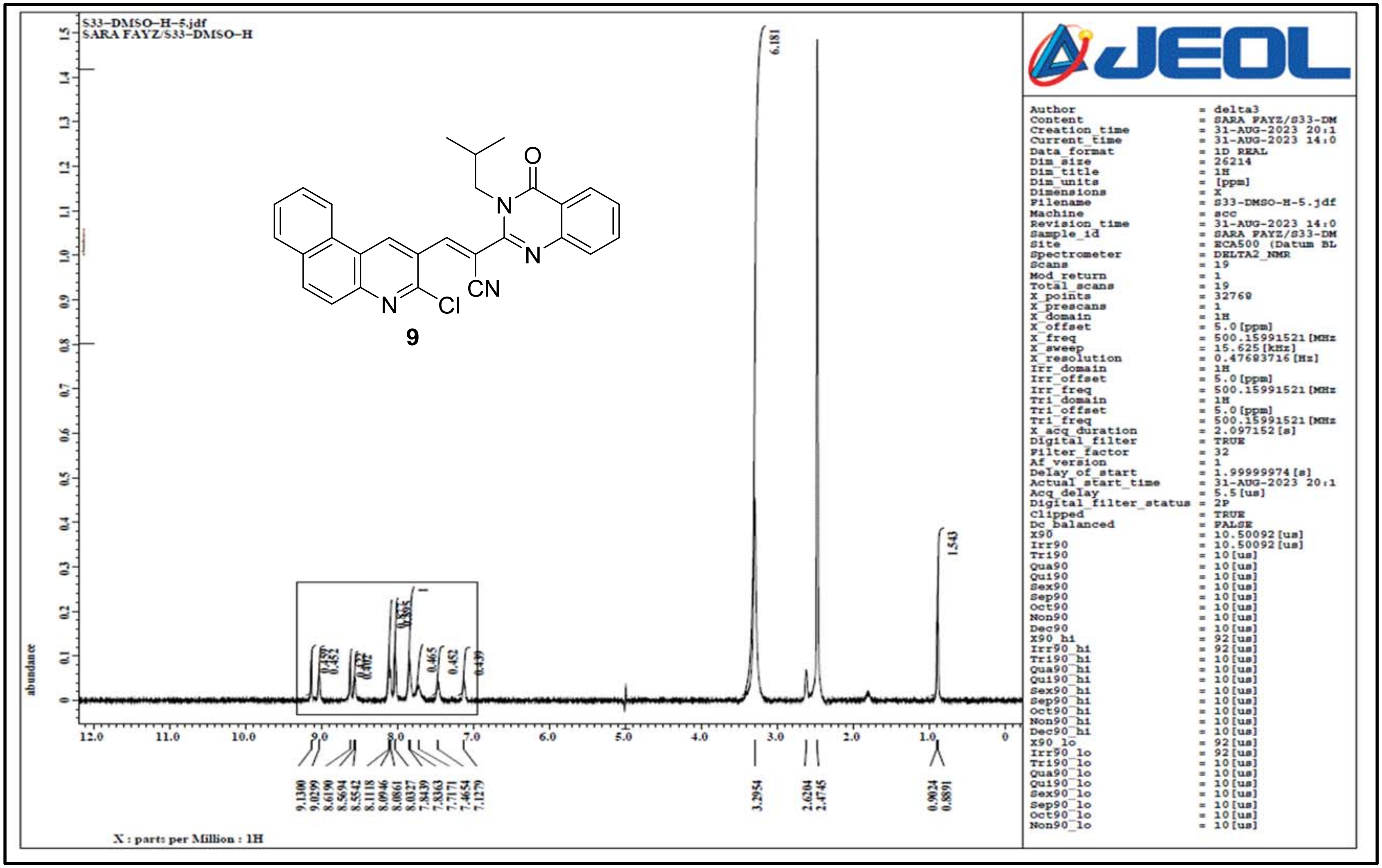

Fig. SD25. ^{1}H NMR spectrum (DMSO-d_6) of compound **9**

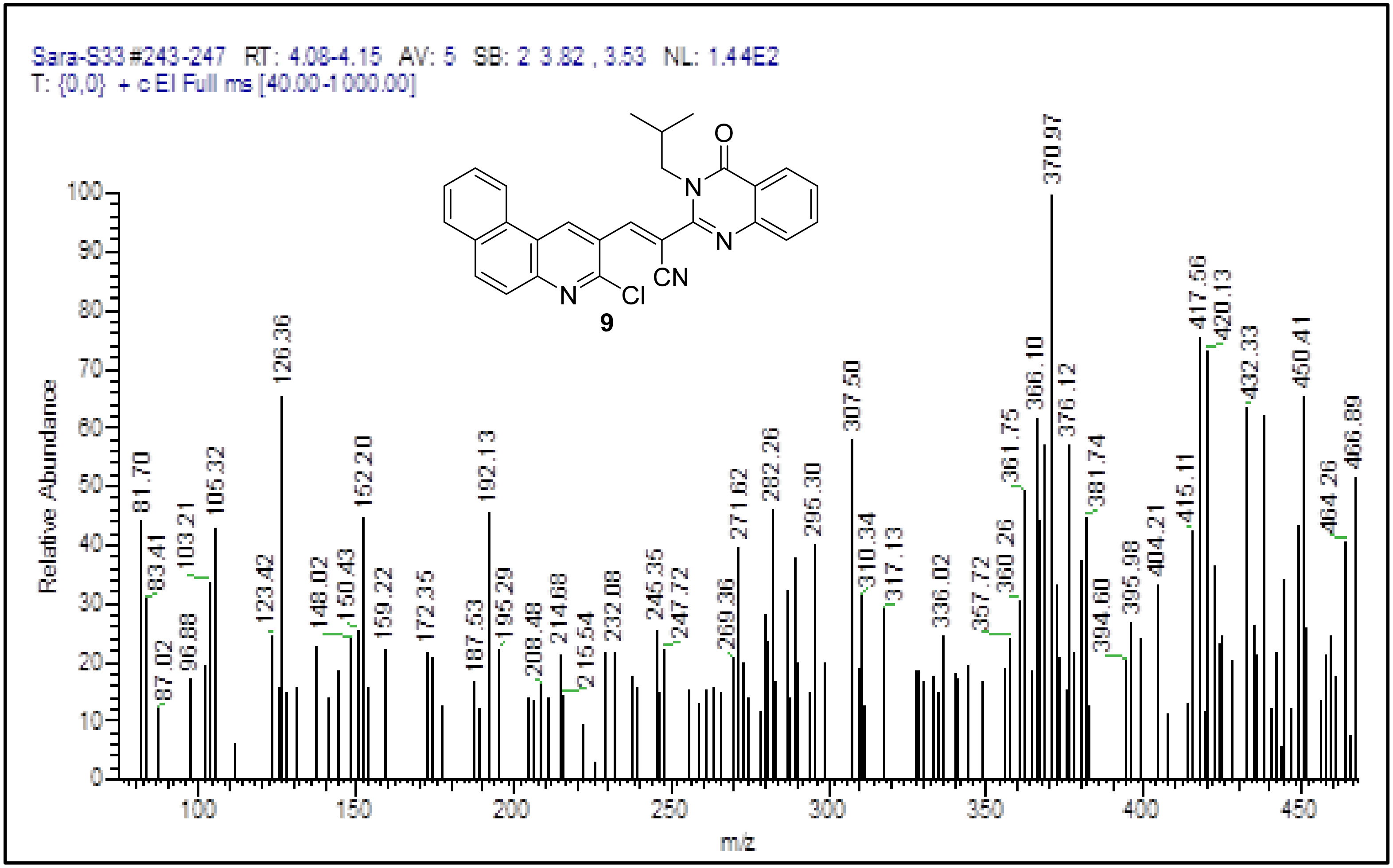

Fig. SD26. Mass spectrum of compound **9**

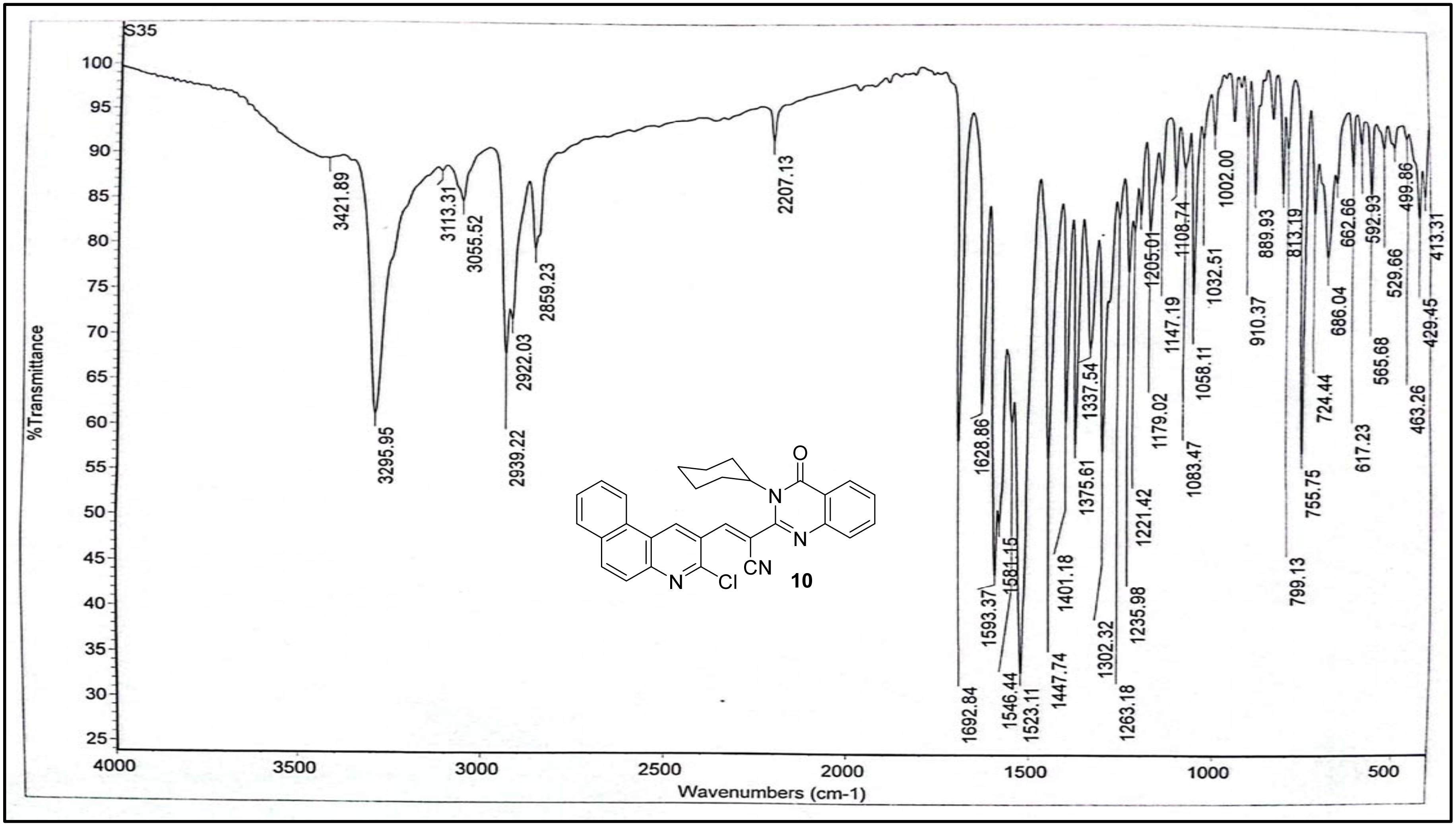

Fig. SD27. IR spectrum of compound **10**

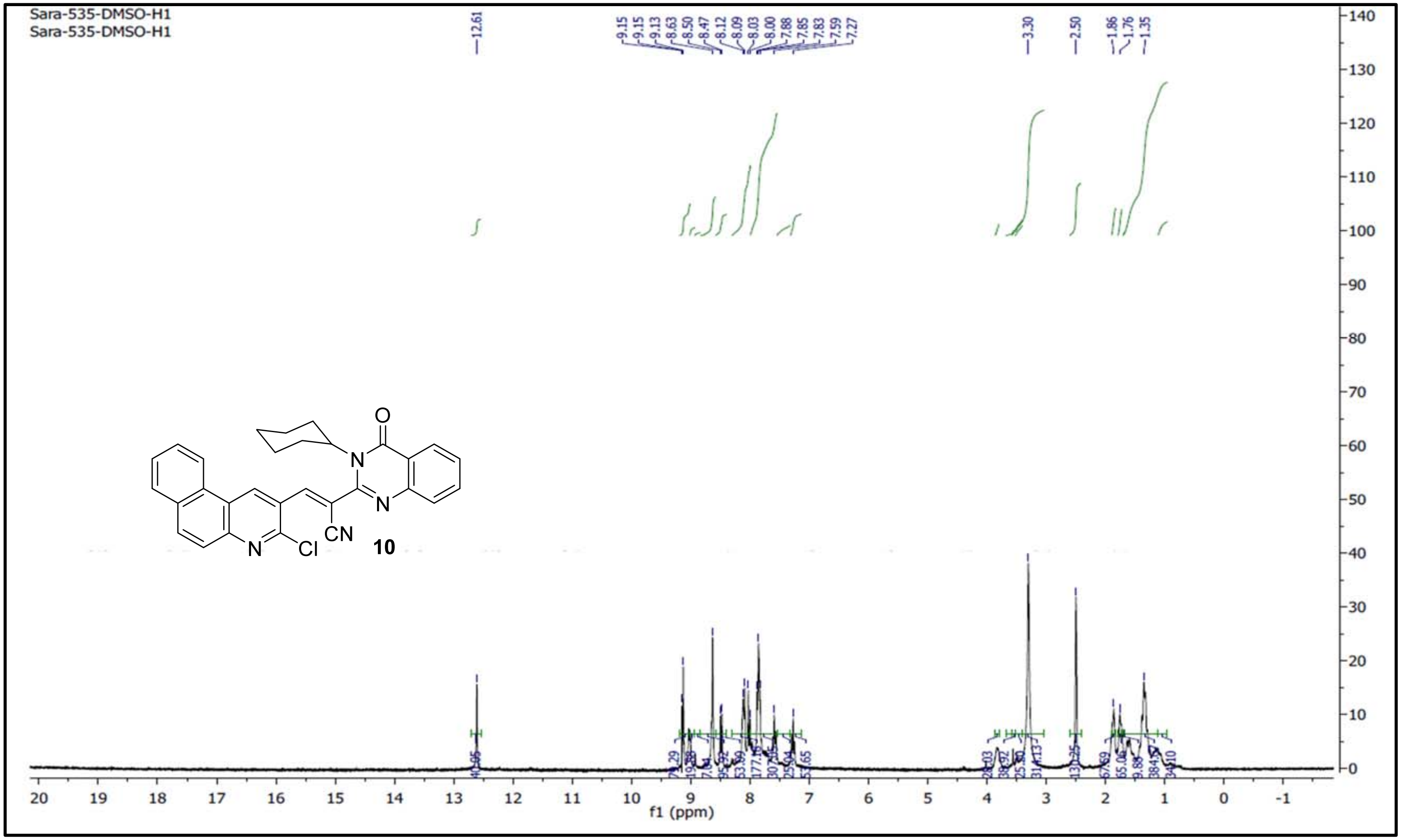

Fig. SD28. ^{1}H NMR spectrum (DMSO-d_6) of compound **10**

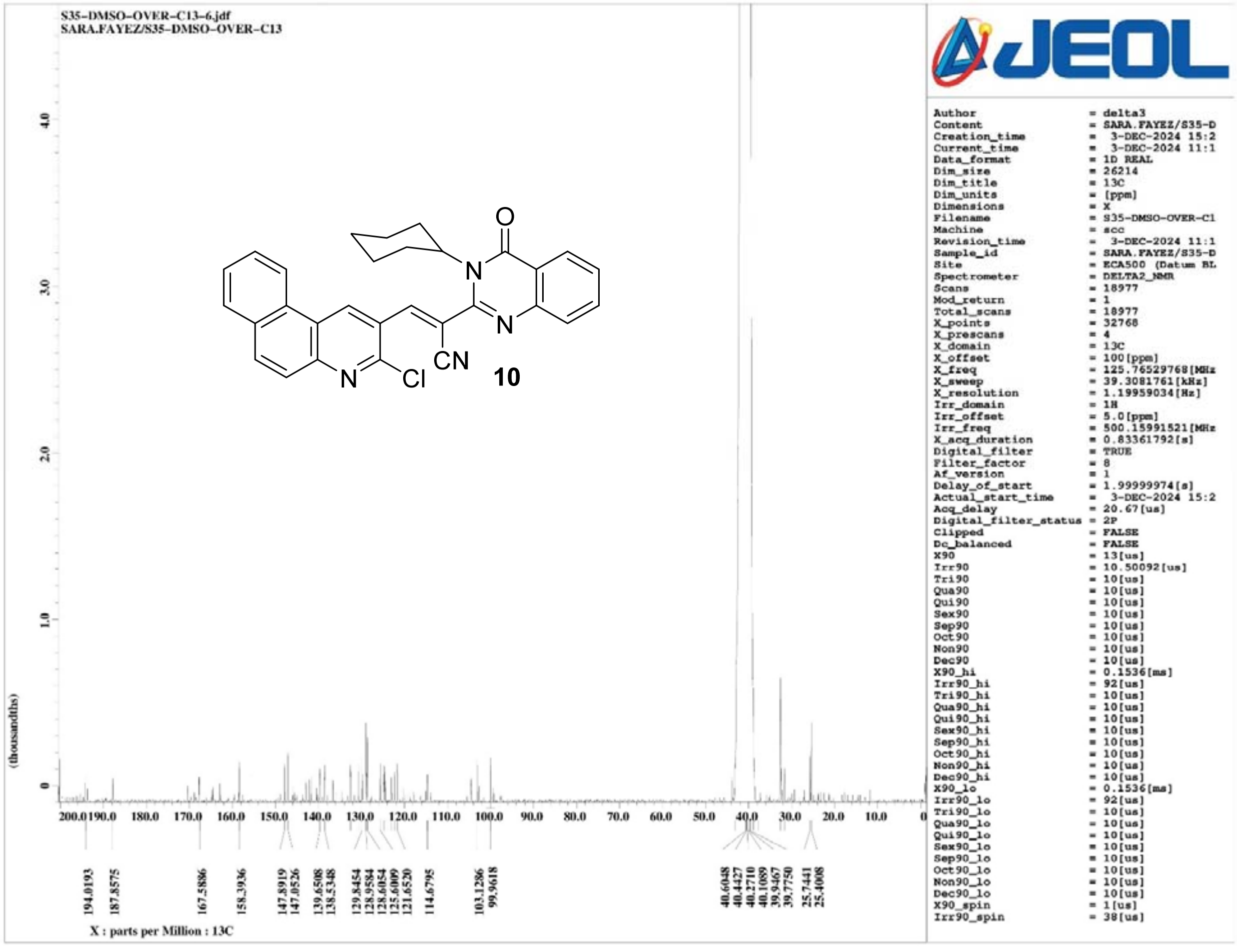

Cont. Fig. SD28. ^{13}C NMR spectrum (DMSO-d_6) of compound **10**

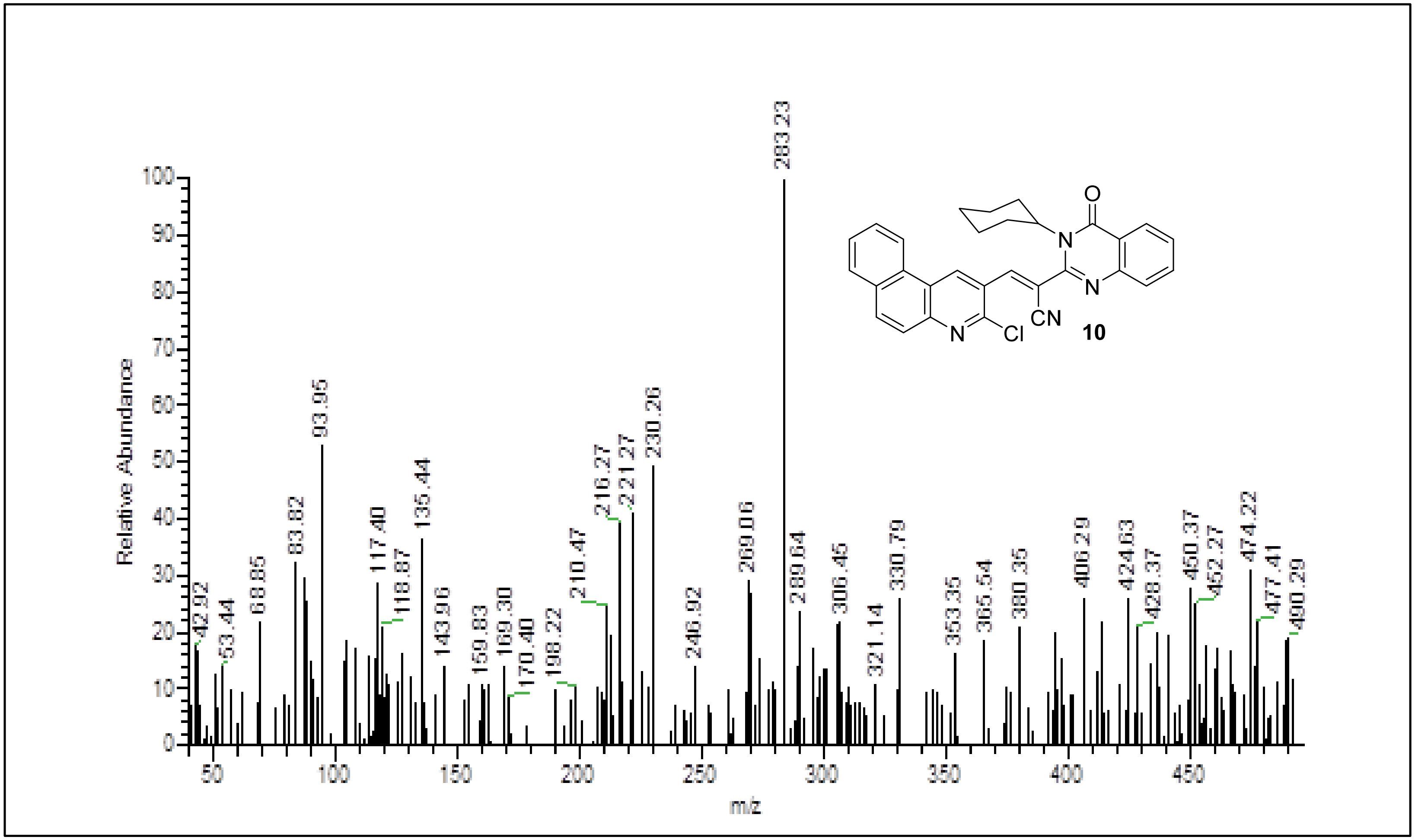

Fig. SD29. Mass spectrum of compound **10**

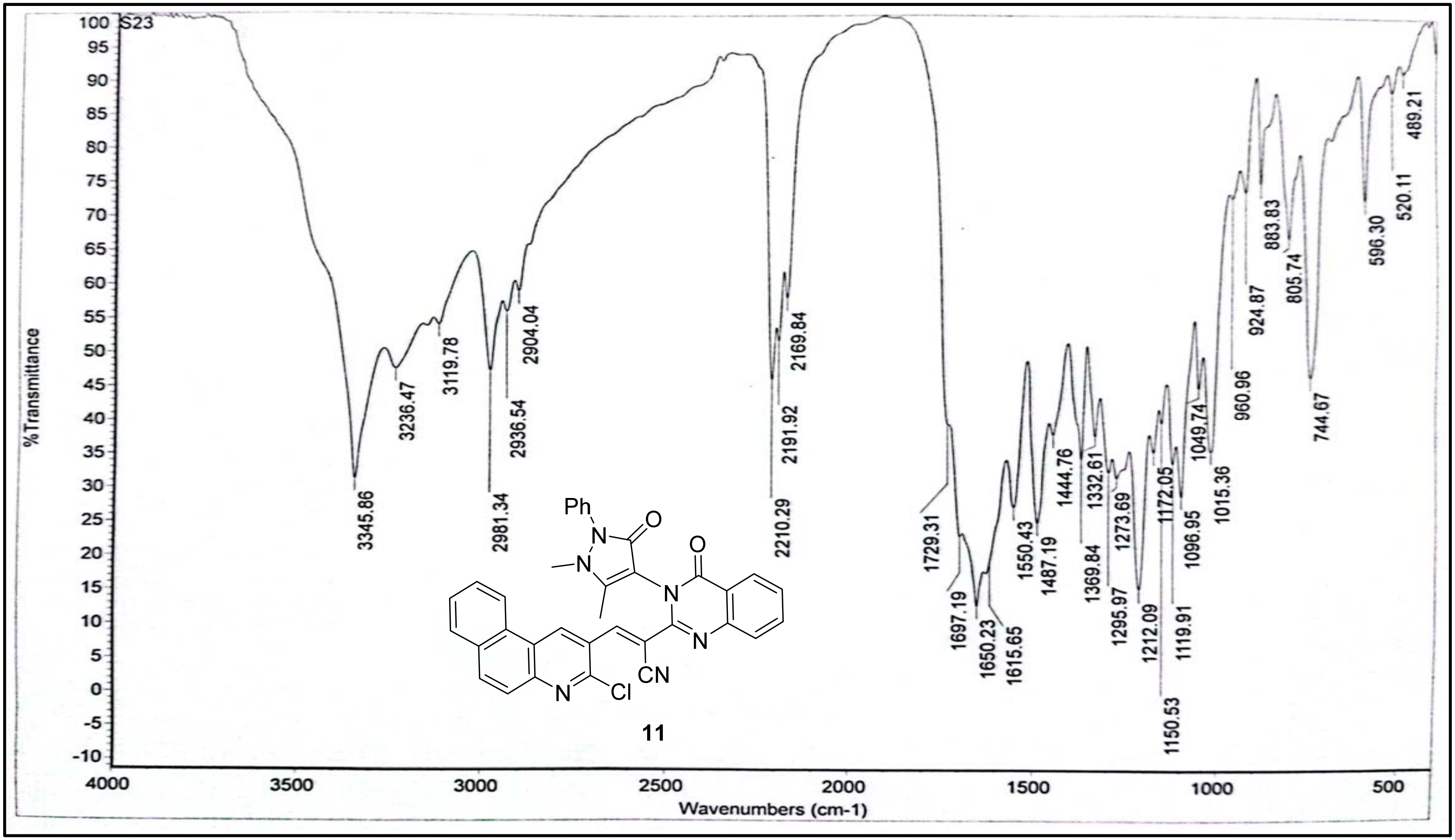

Fig. SD30. IR spectrum of compound **11**

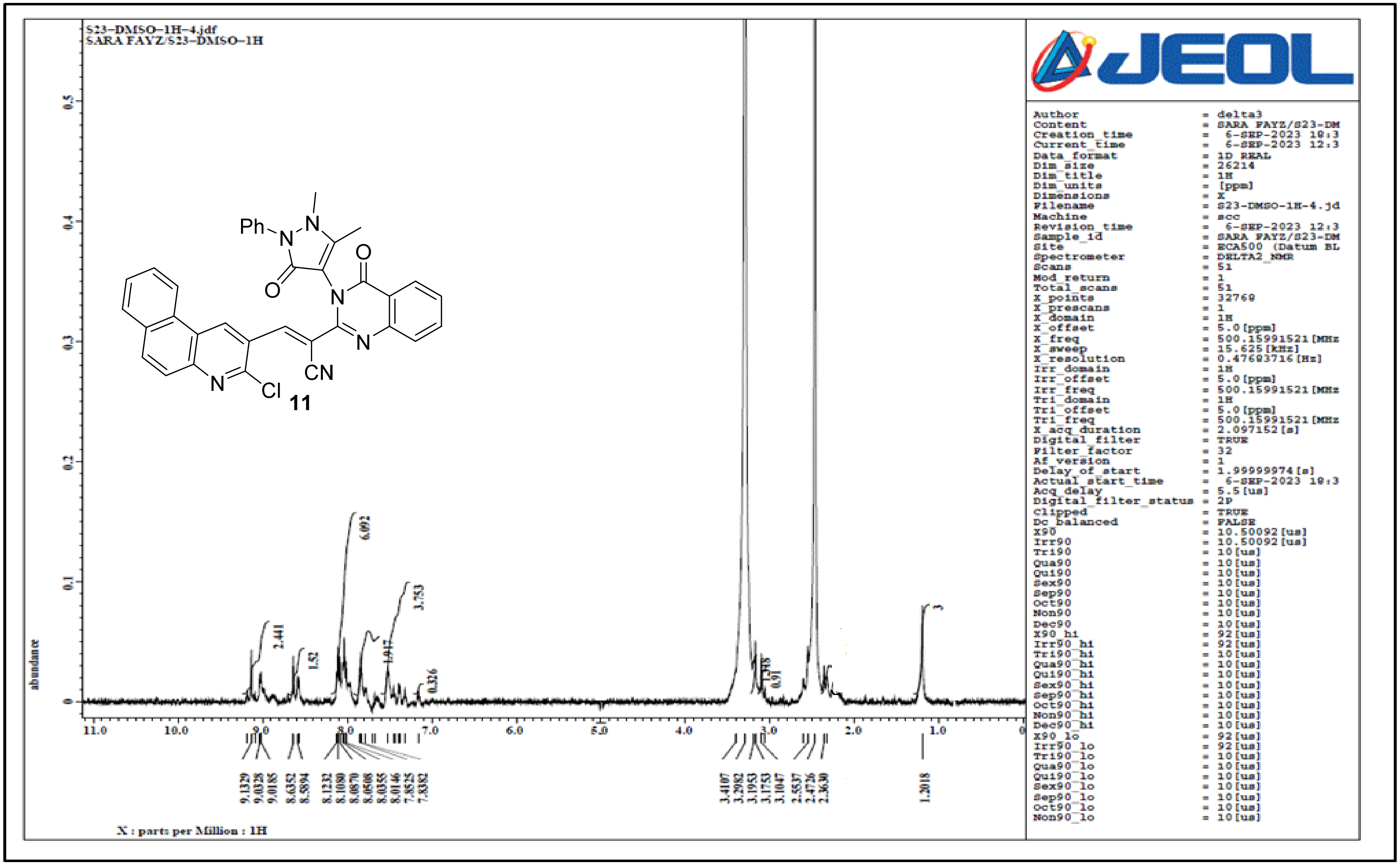

Fig. SD31. ^{1}H NMR spectrum (DMSO-d_6) of compound **11**

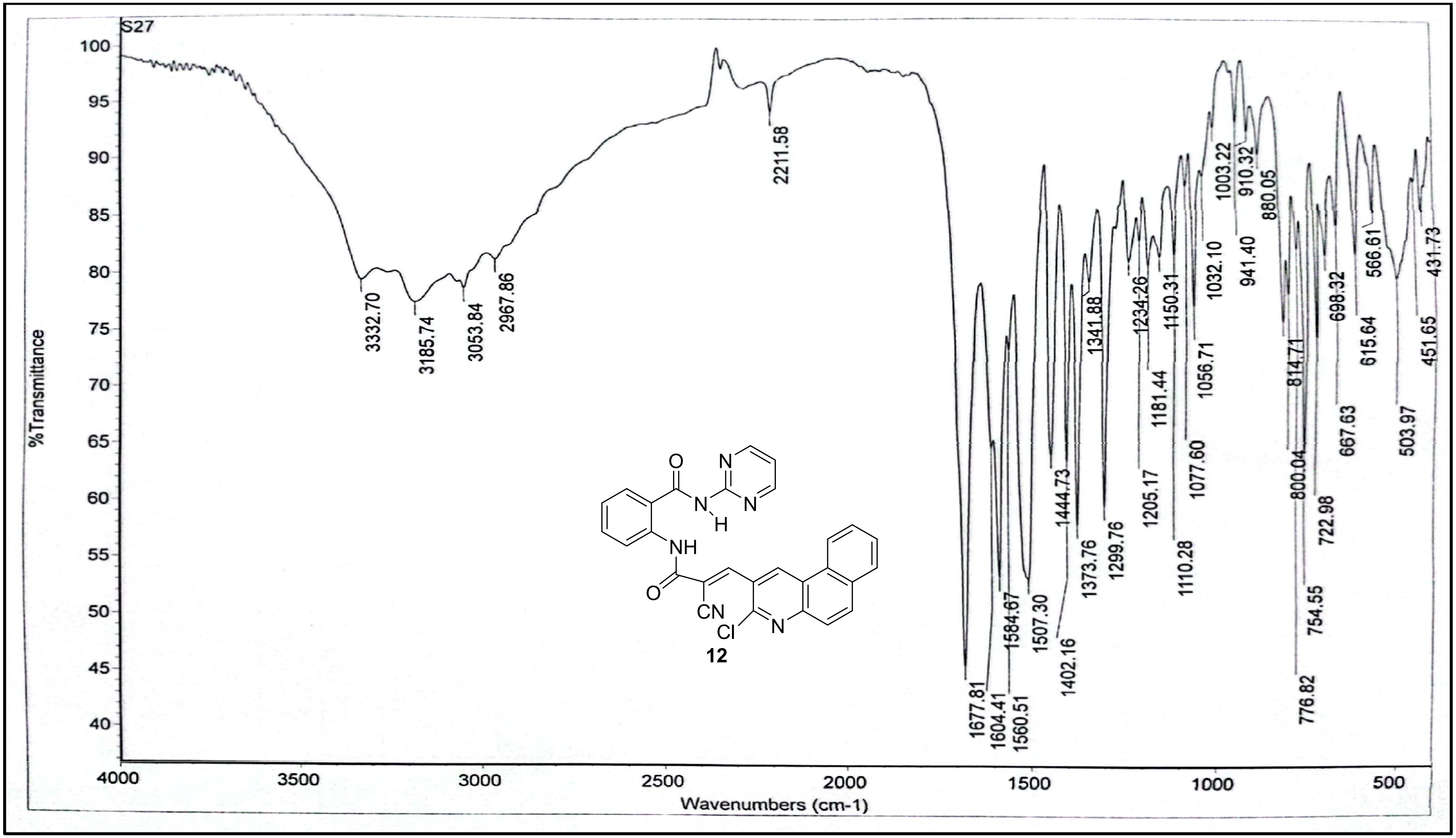

Fig. SD32. IR spectrum of compound **12**

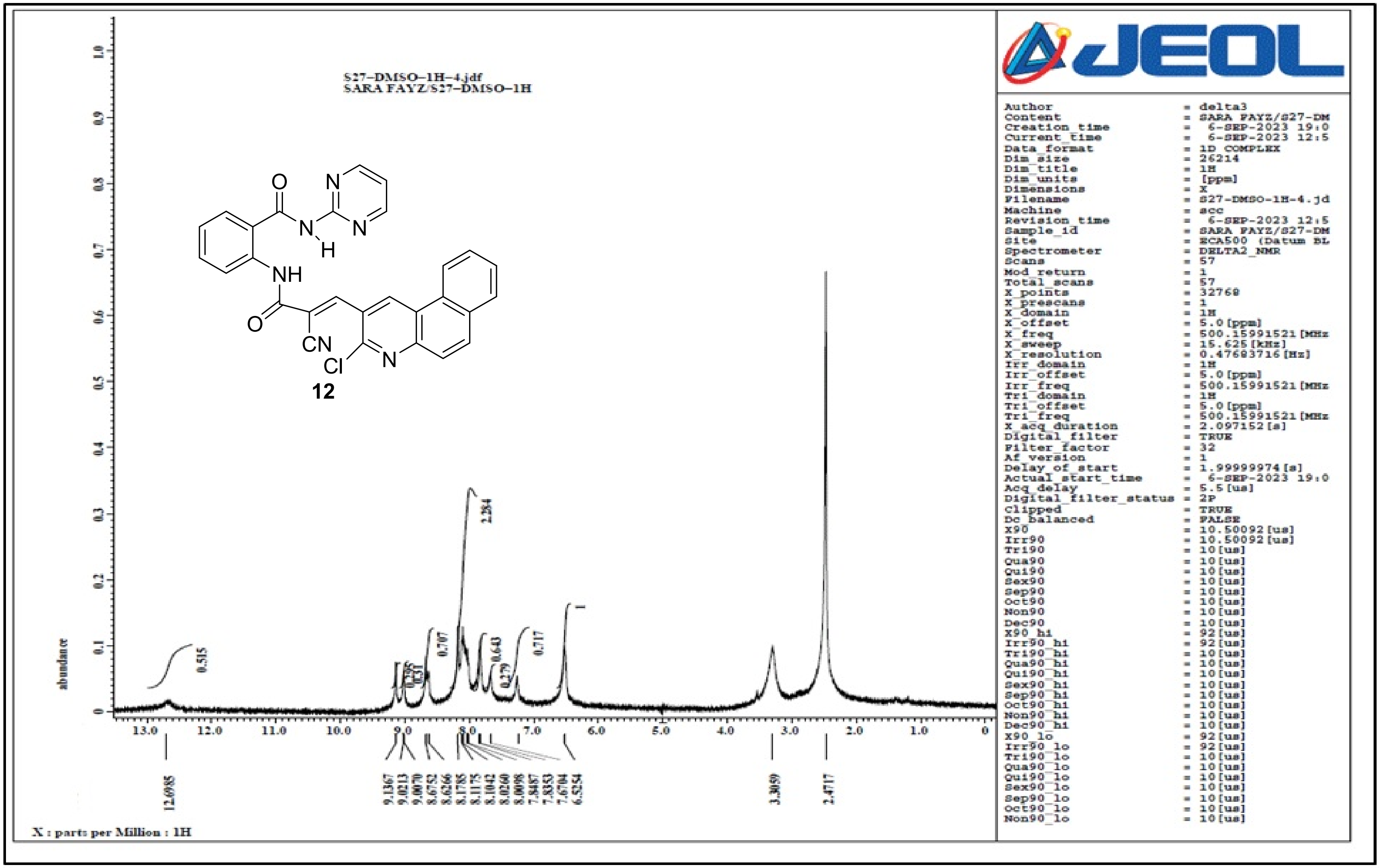

Fig. SD33. ^{1}H NMR spectrum (DMSO-d_6) of compound **12**

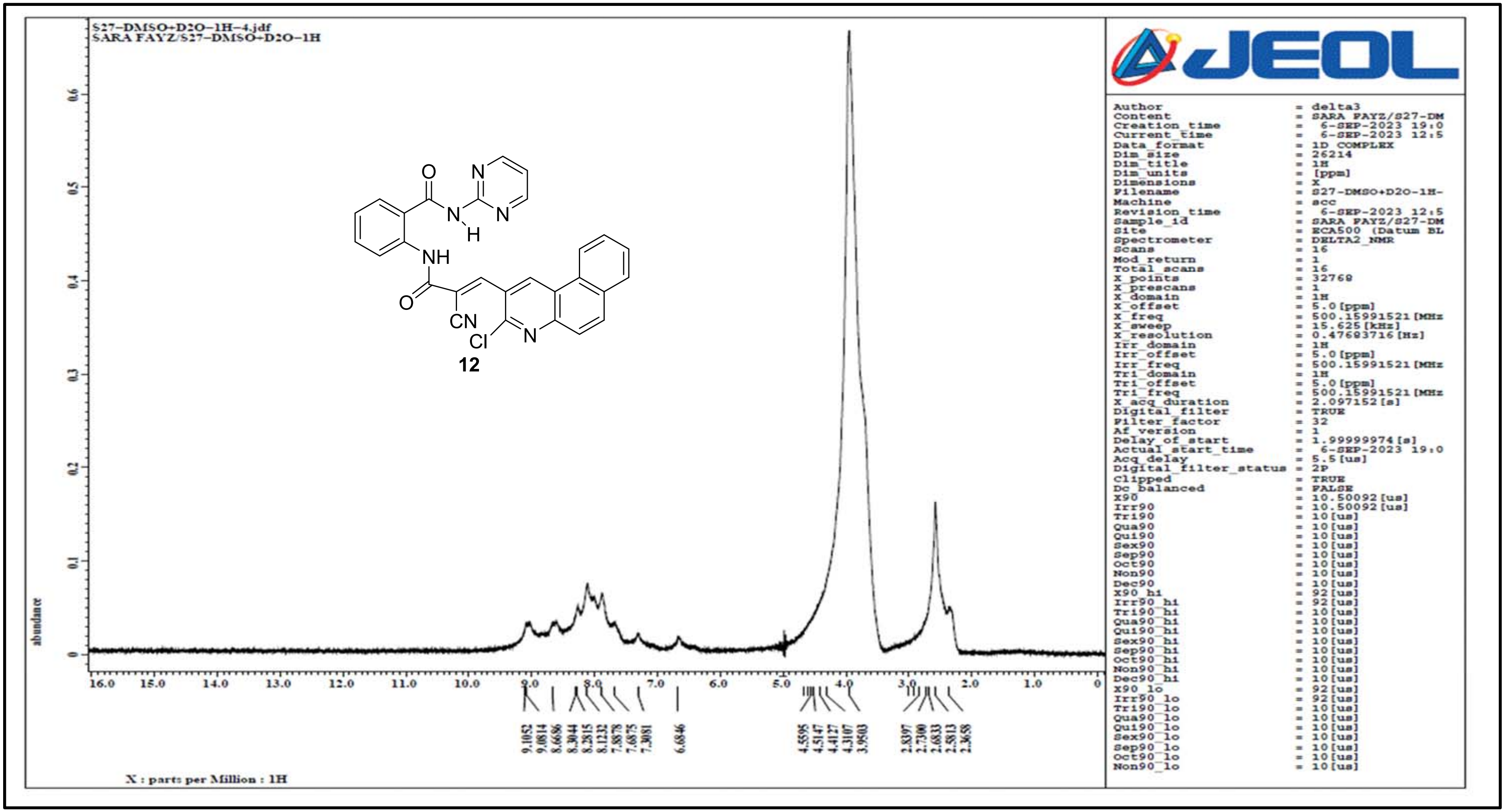

Fig. SD34. ^{1}H NMR spectrum (DMSO-d_6+D_2O) of compound **12**

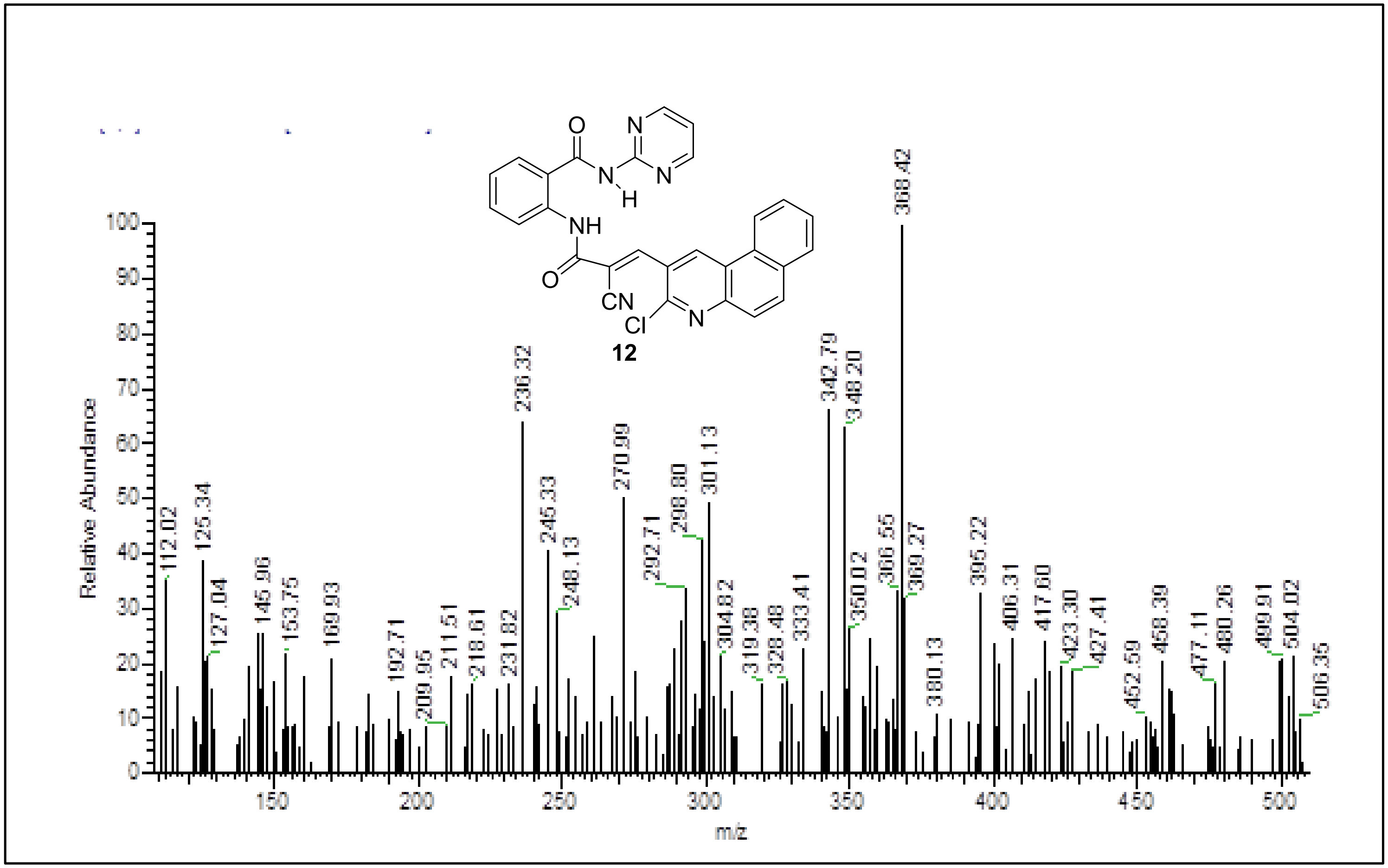

Fig. SD35. Mass spectrum of compound **12**

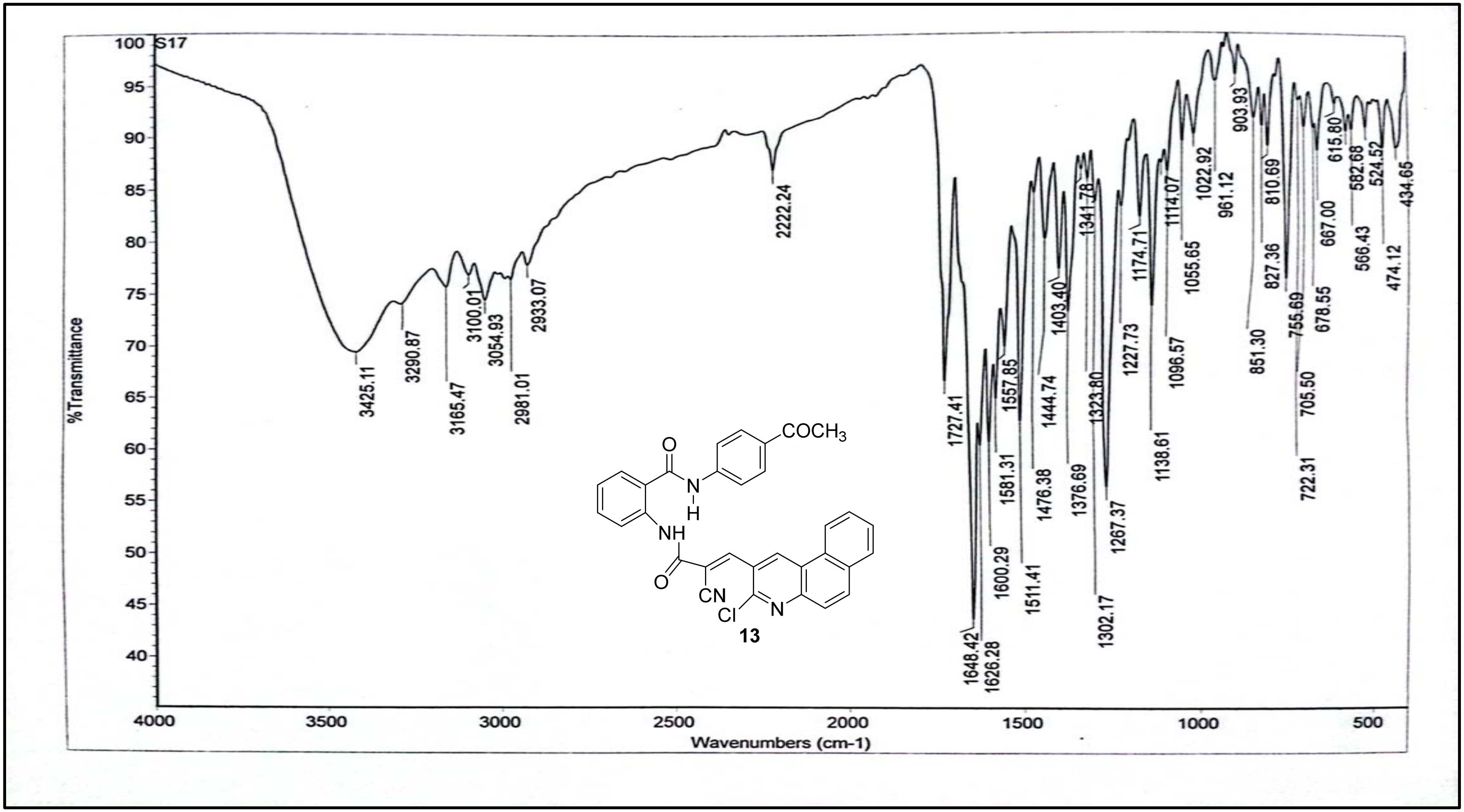

Fig. SD36. IR spectrum of compound **13**

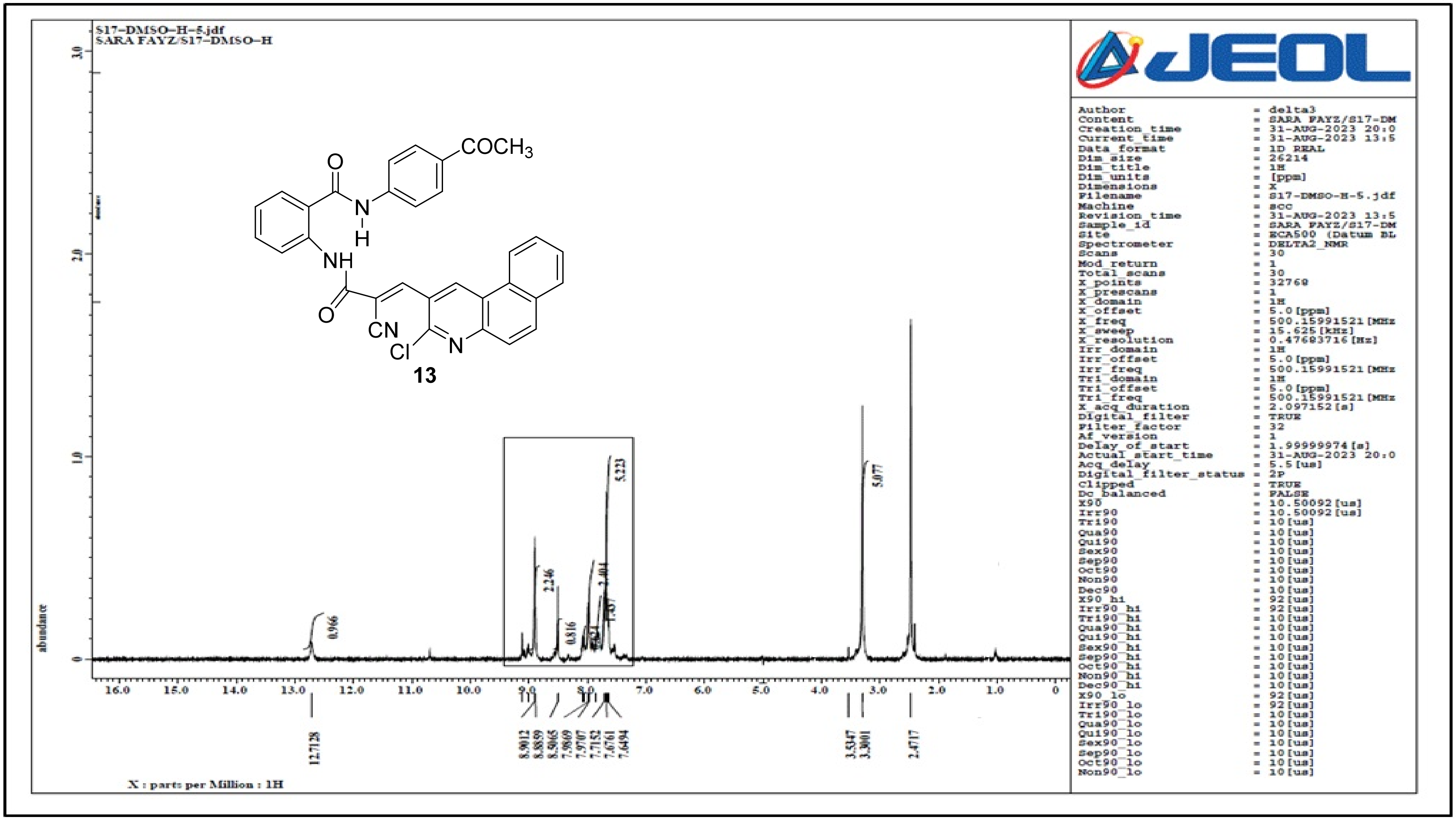

Fig. SD37. ^{1}H NMR spectrum (DMSO-d_6) of compound **13**

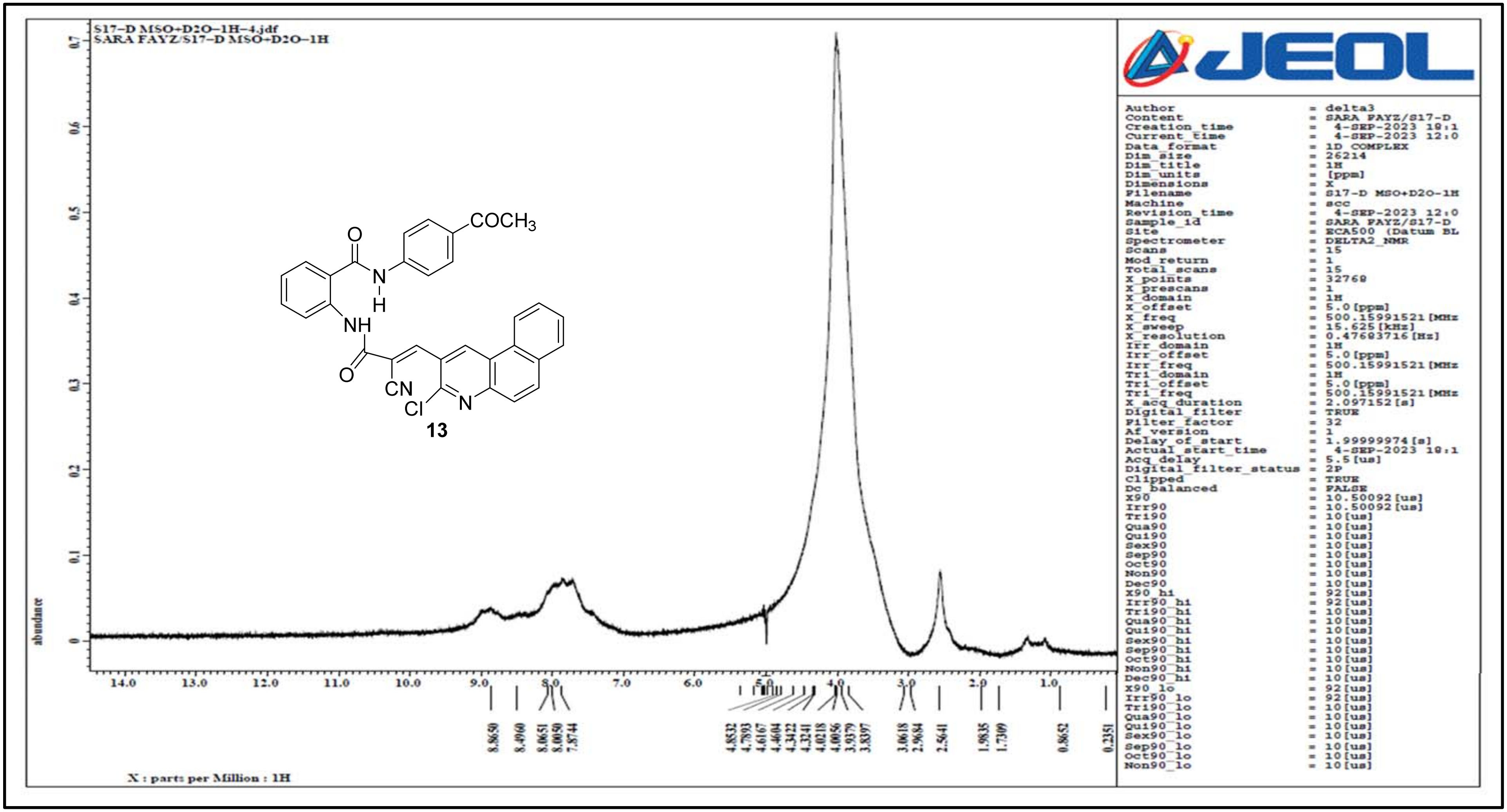

Fig. SD38. ^{1}H NMR spectrum (DMSO-d_6+D_2O) of compound **13**

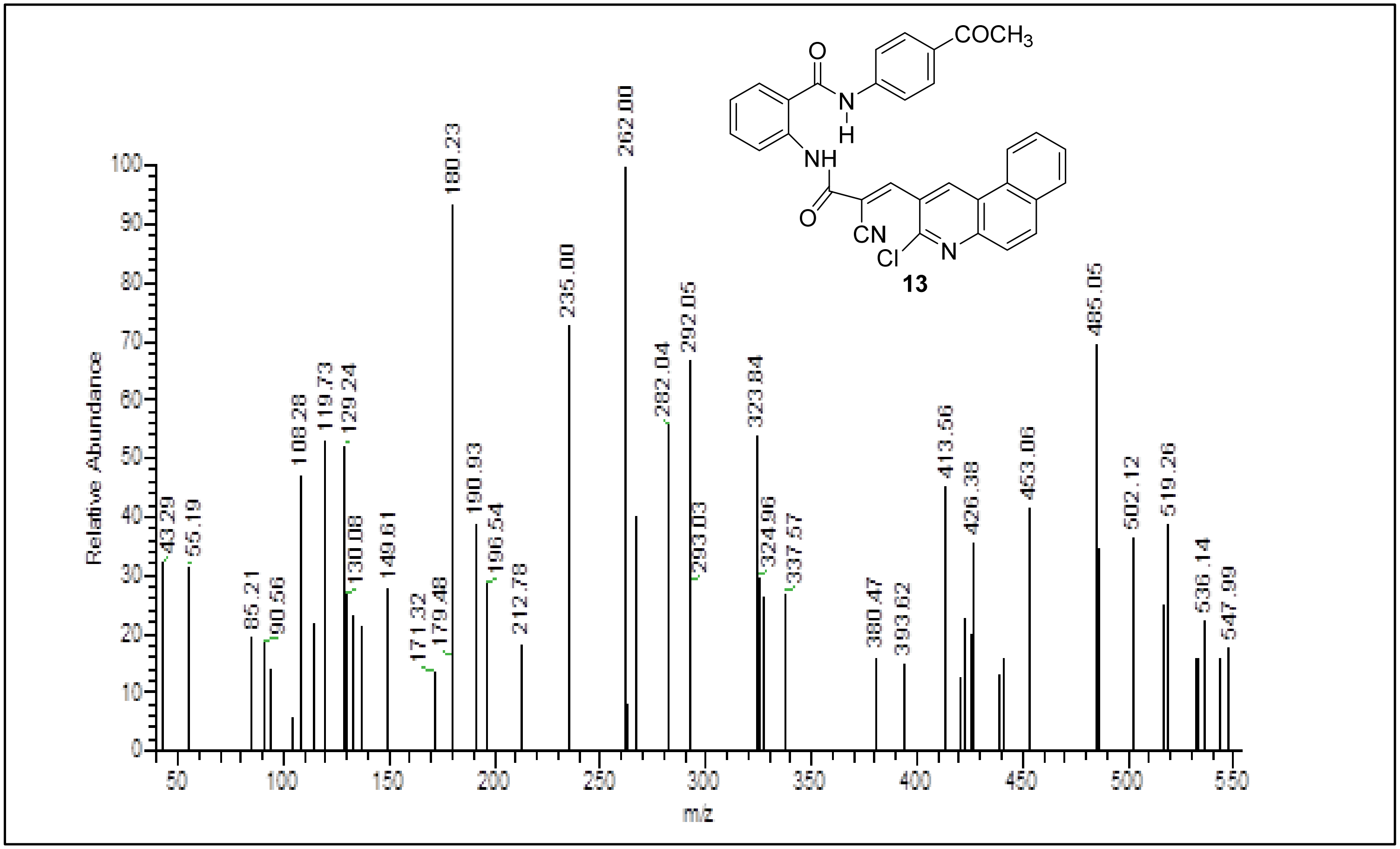

Fig. SD39. Mass spectrum of compound **13**

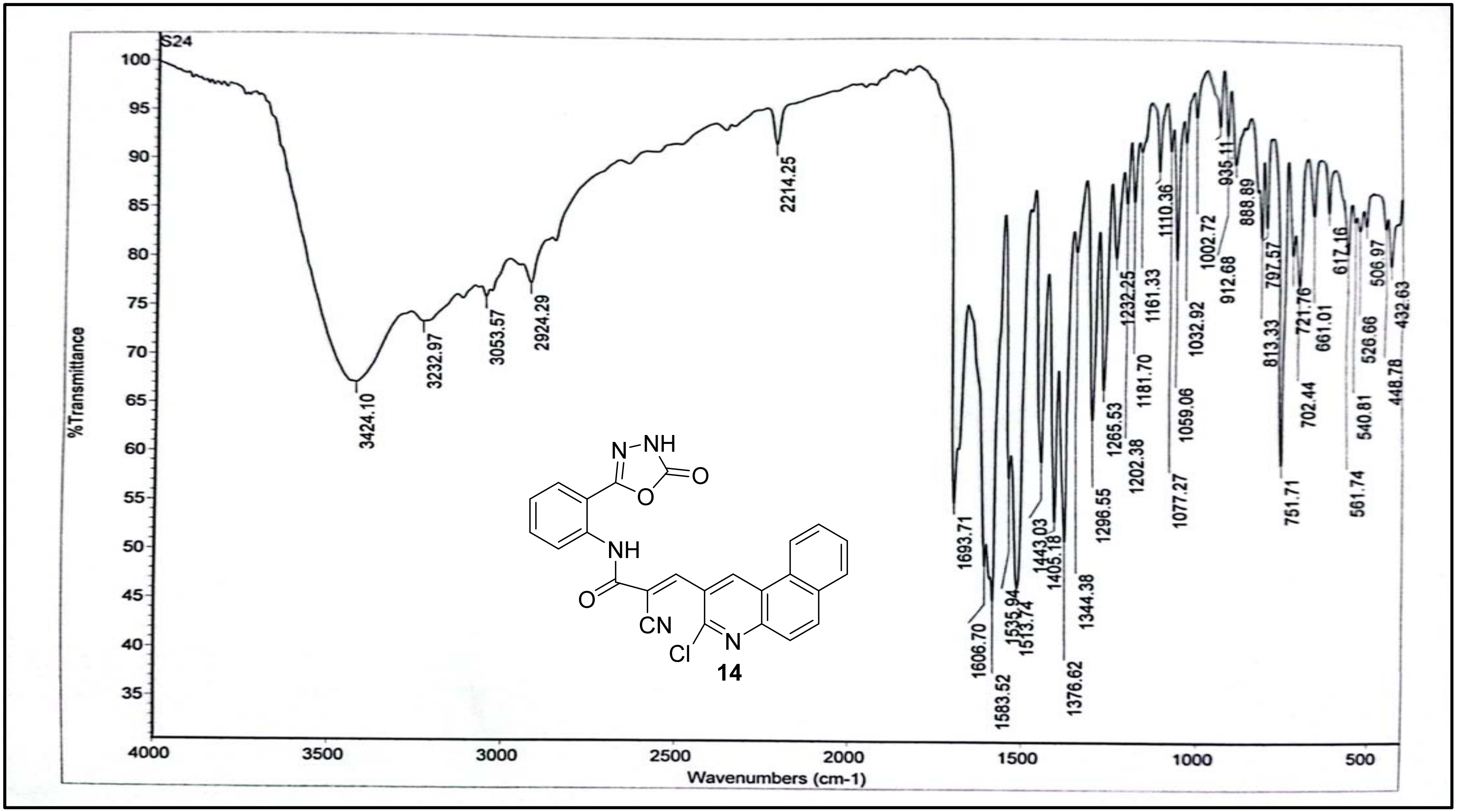

Fig. SD40. IR spectrum of compound **14**

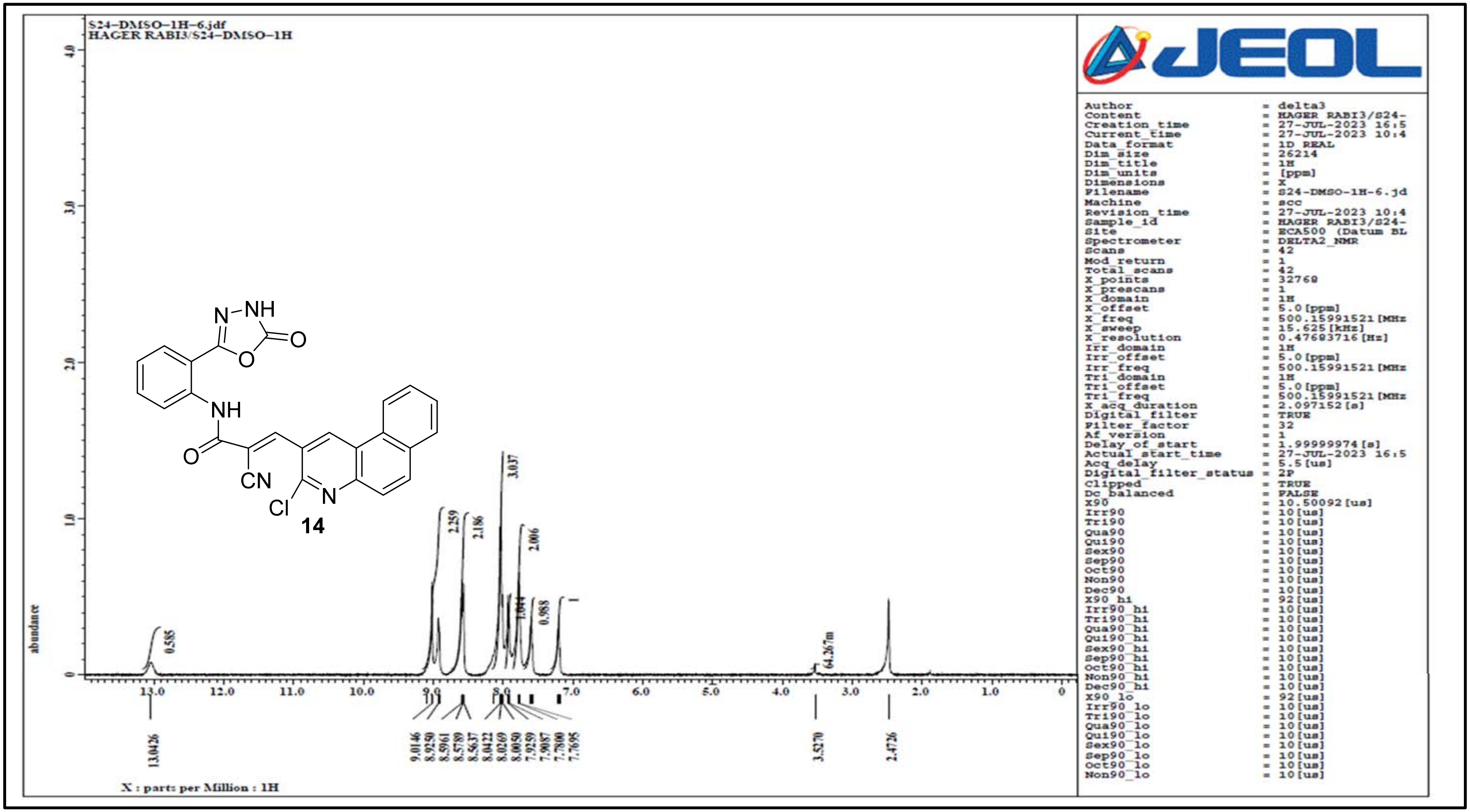

Fig. SD41. ^{1}H NMR spectrum (DMSO-d_6) of compound **14**

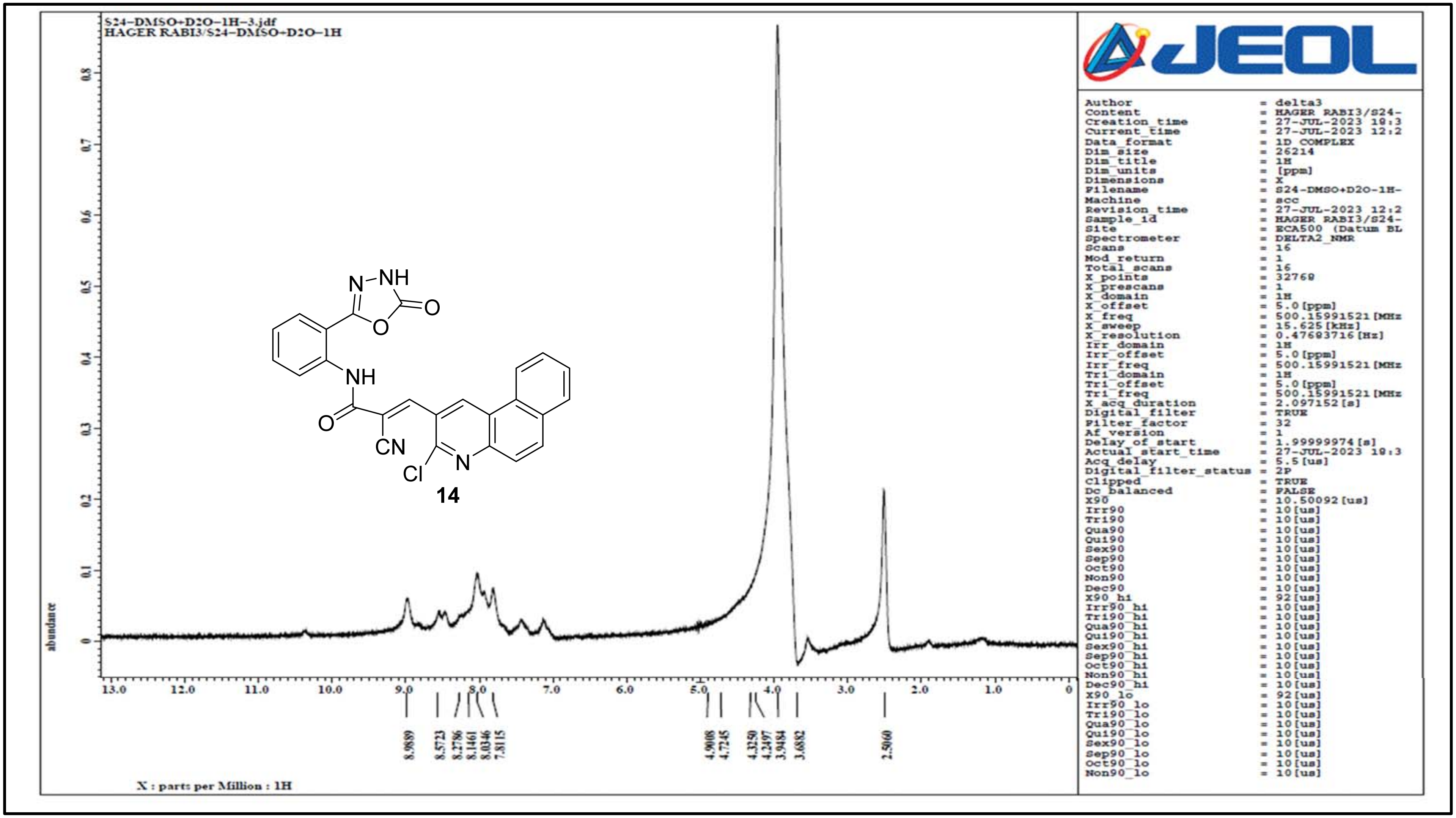

Fig. SD42. ^{1}H NMR spectrum (DMSO-d_6+D_2O) of compound **14**

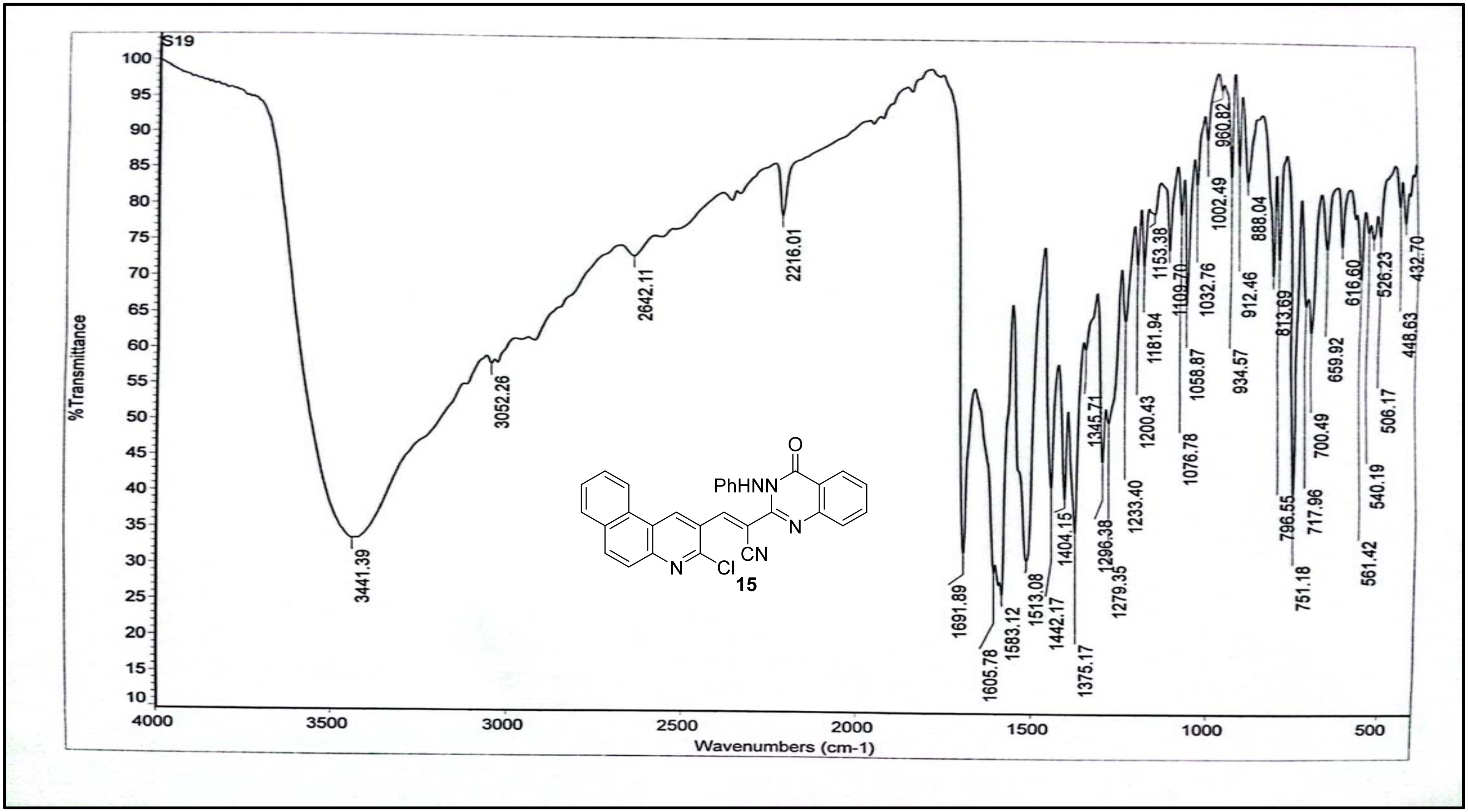

Fig. SD43. IR spectrum of compound **15**

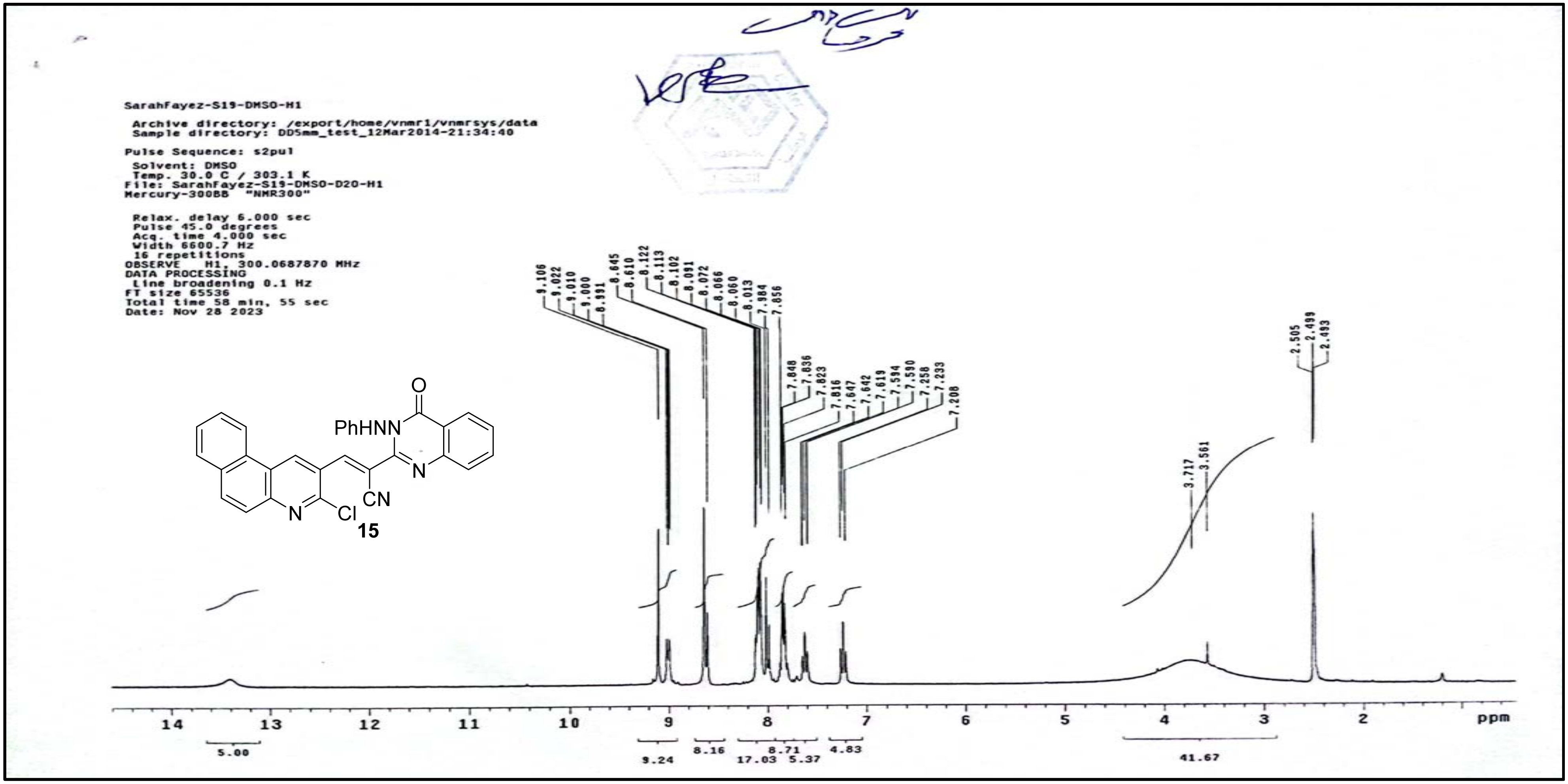

Fig. SD44. ^{1}H NMR spectrum (DMSO-d_6) of compound **15**

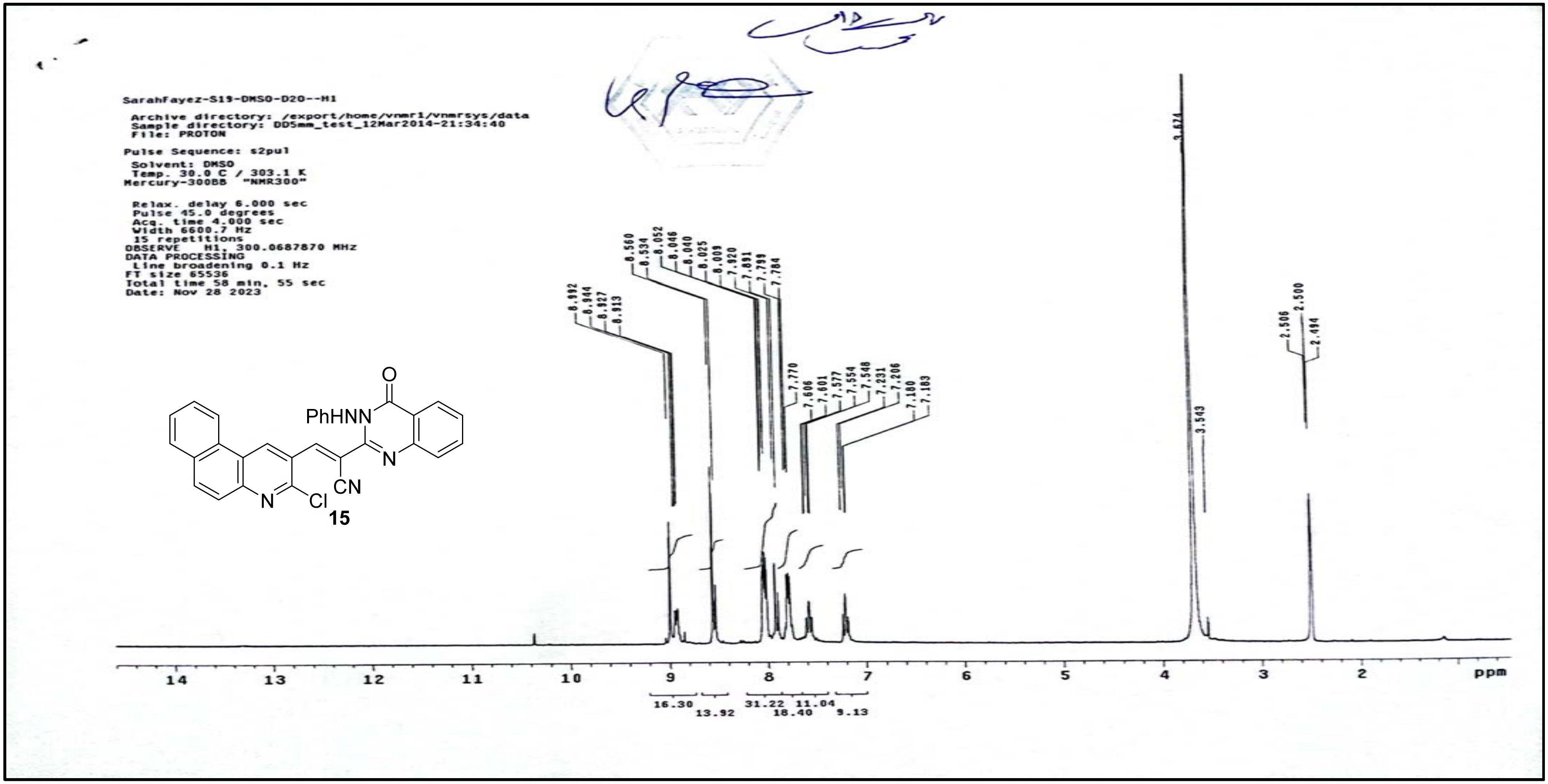

Fig. SD45. ^{1}H NMR spectrum (DMSO-d_6+D_2O) of compound **15**

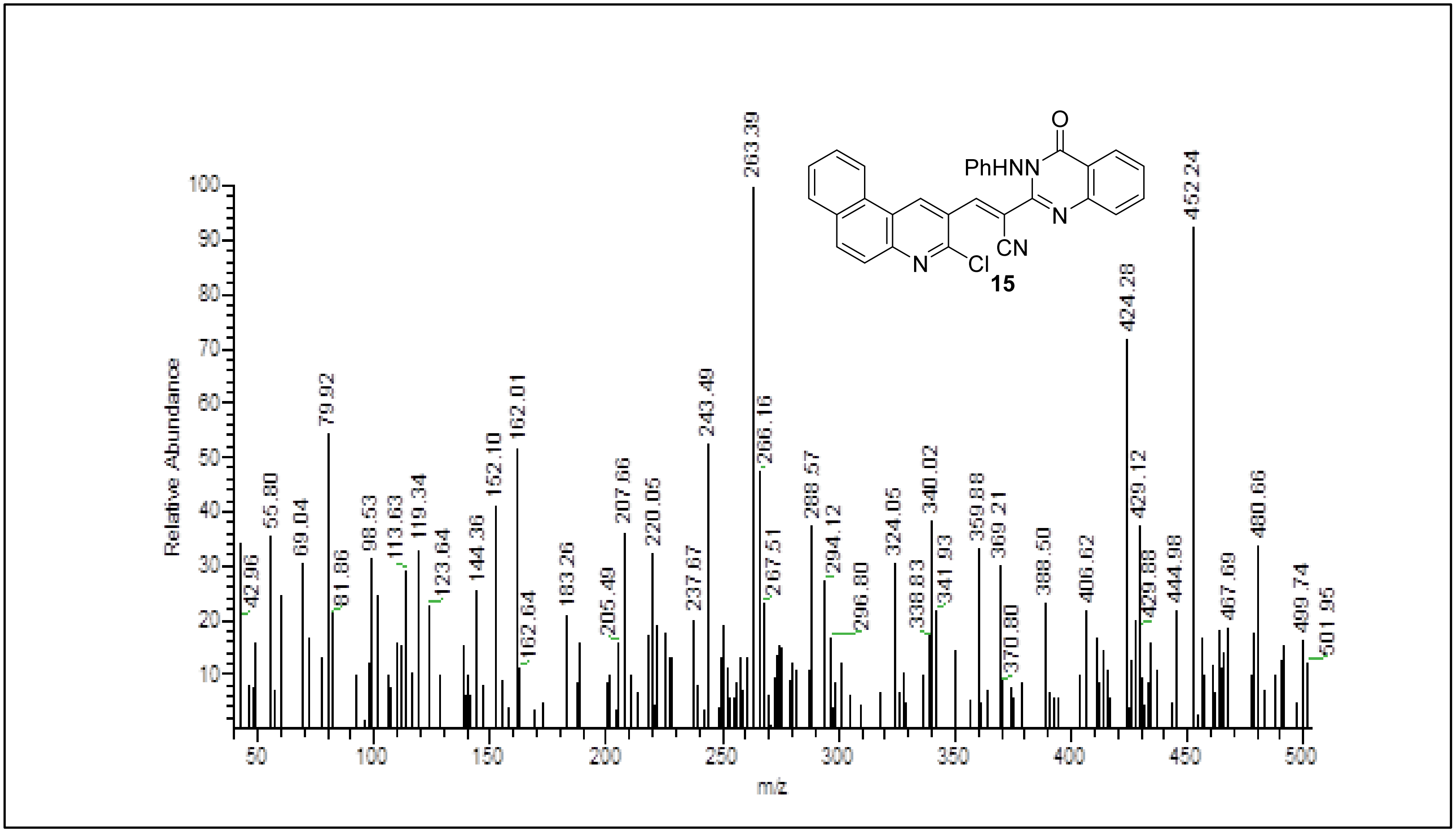

Fig. SD46. Mass spectrum of compound **15**

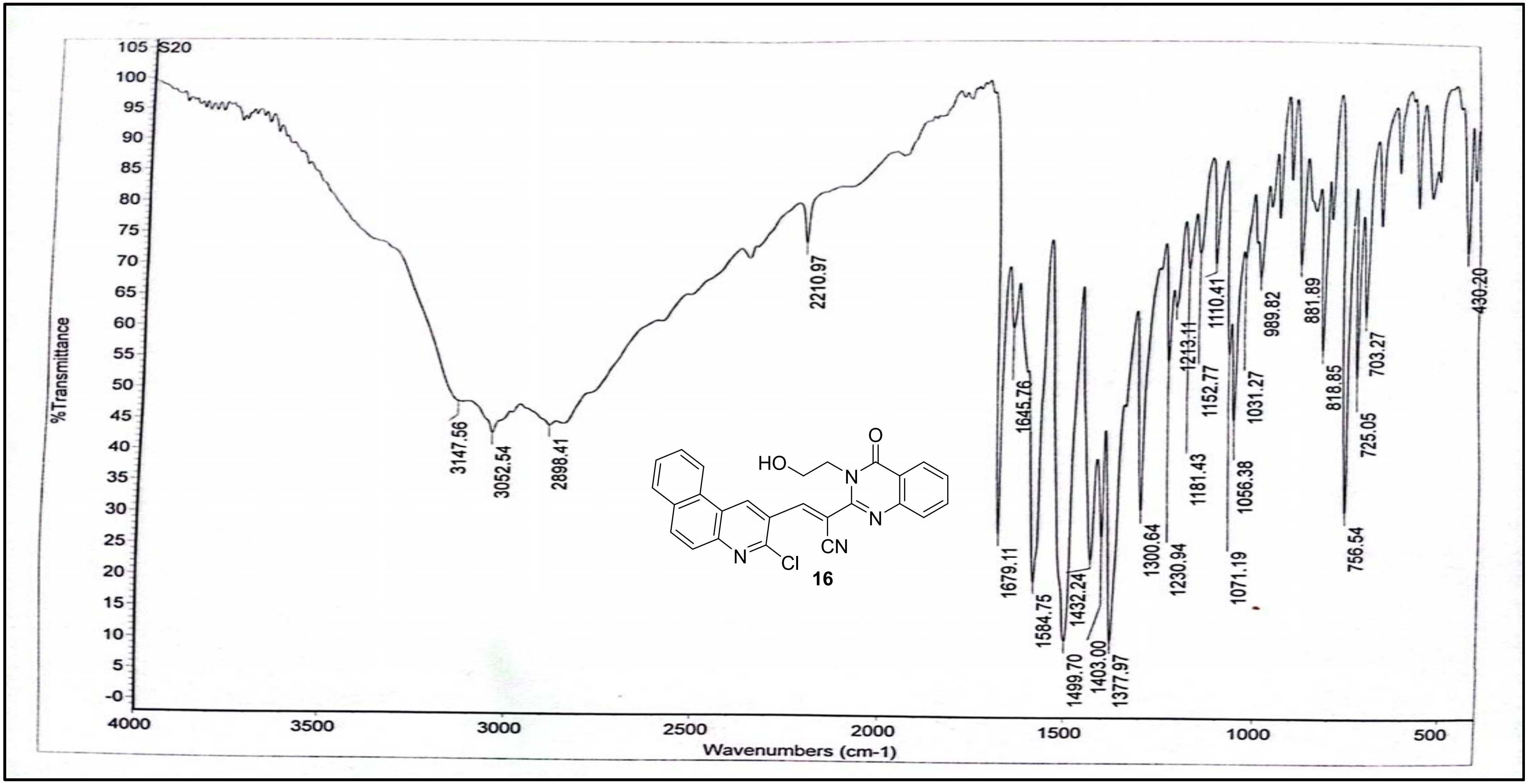

Fig. SD47. IR spectrum of compound **16**

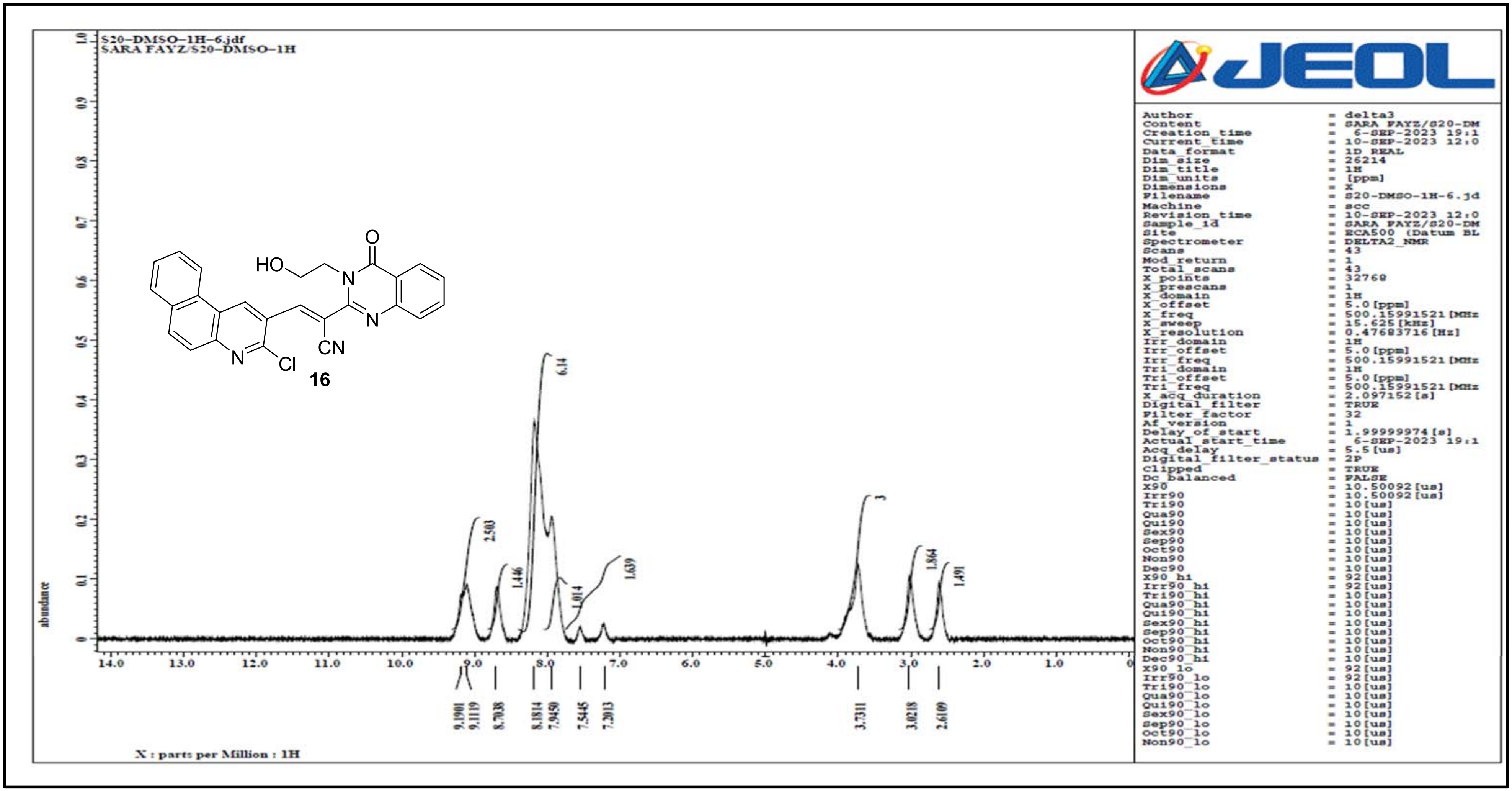

Fig. SD48. ^{1}H NMR spectrum (DMSO-d_6) of compound **16**

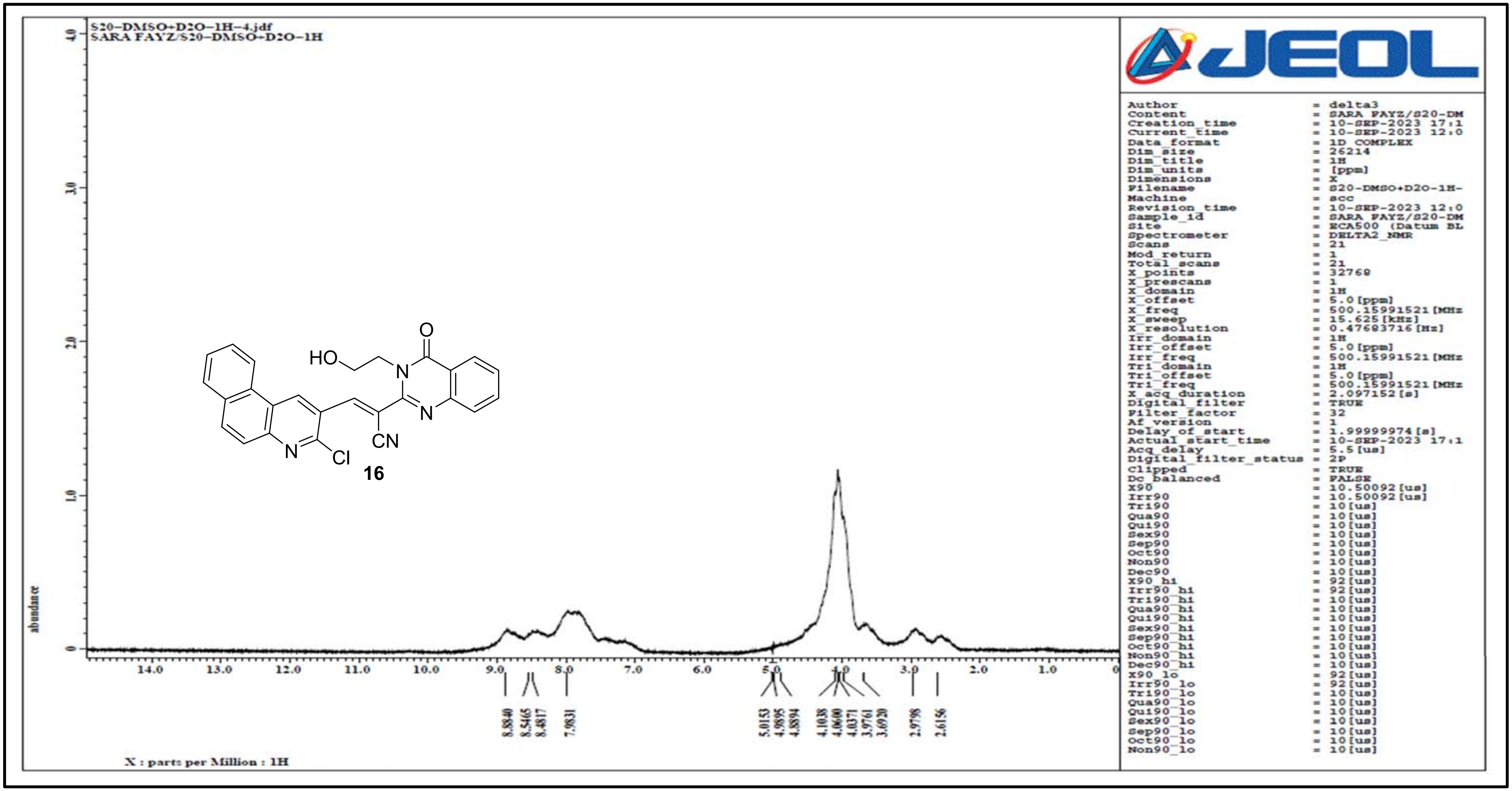

Fig. SD49. ^{1}H NMR spectrum (DMSO-d_6+D_2O) of compound **16**

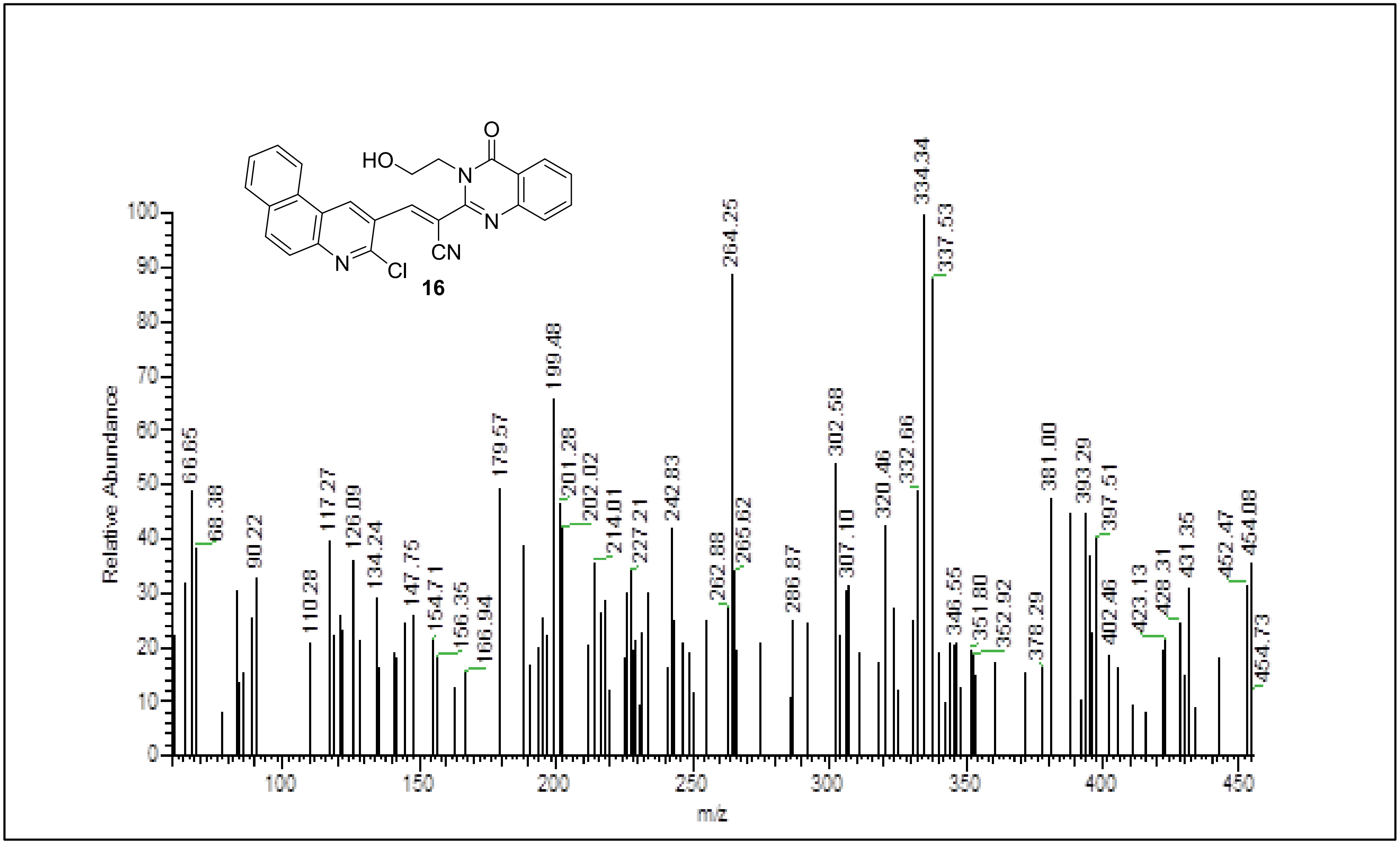

Fig. SD50. Mass spectrum (DMSO-d_6) of compound **16**

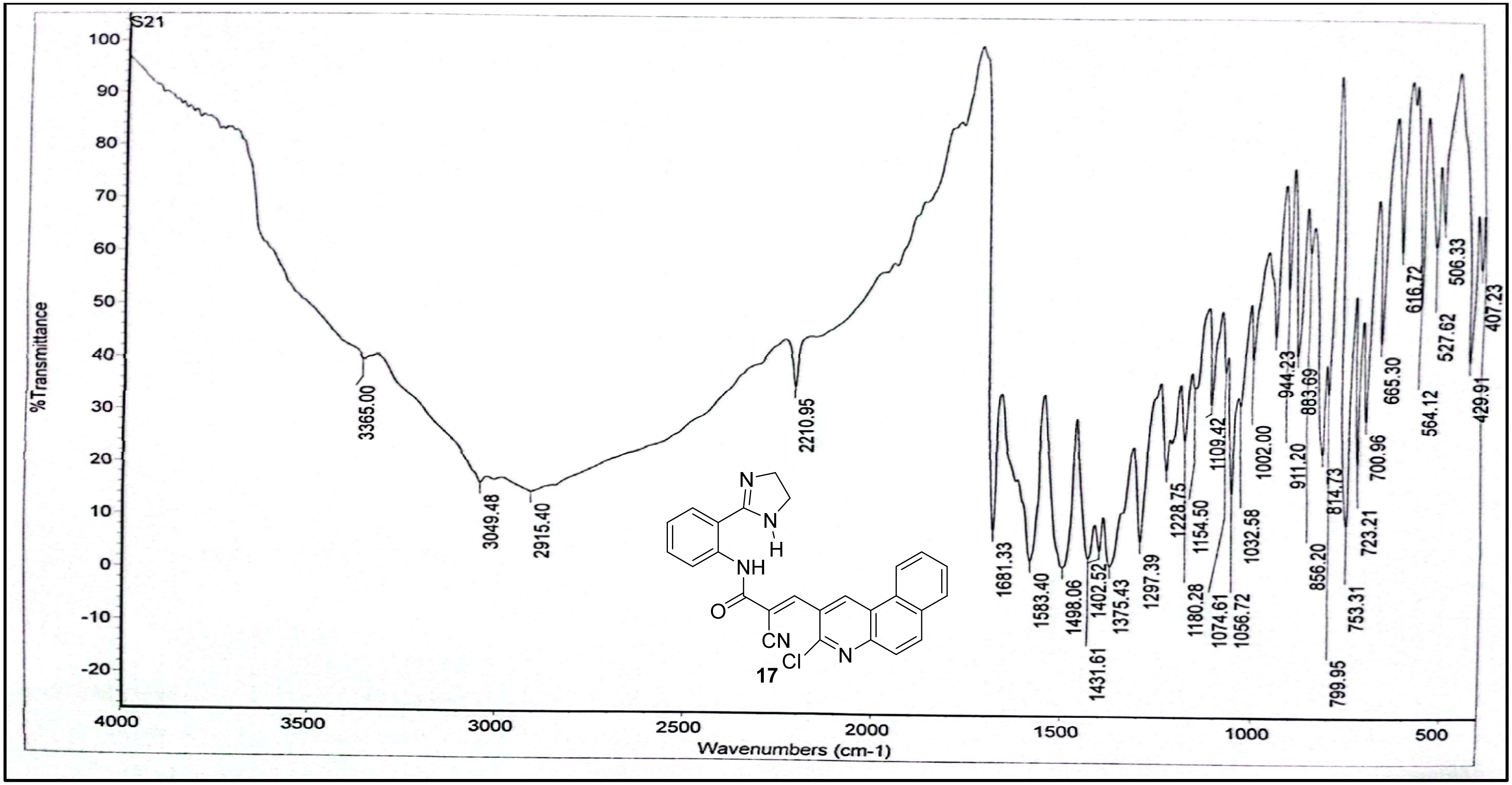

Fig. SD51. IR spectrum of compound **17**

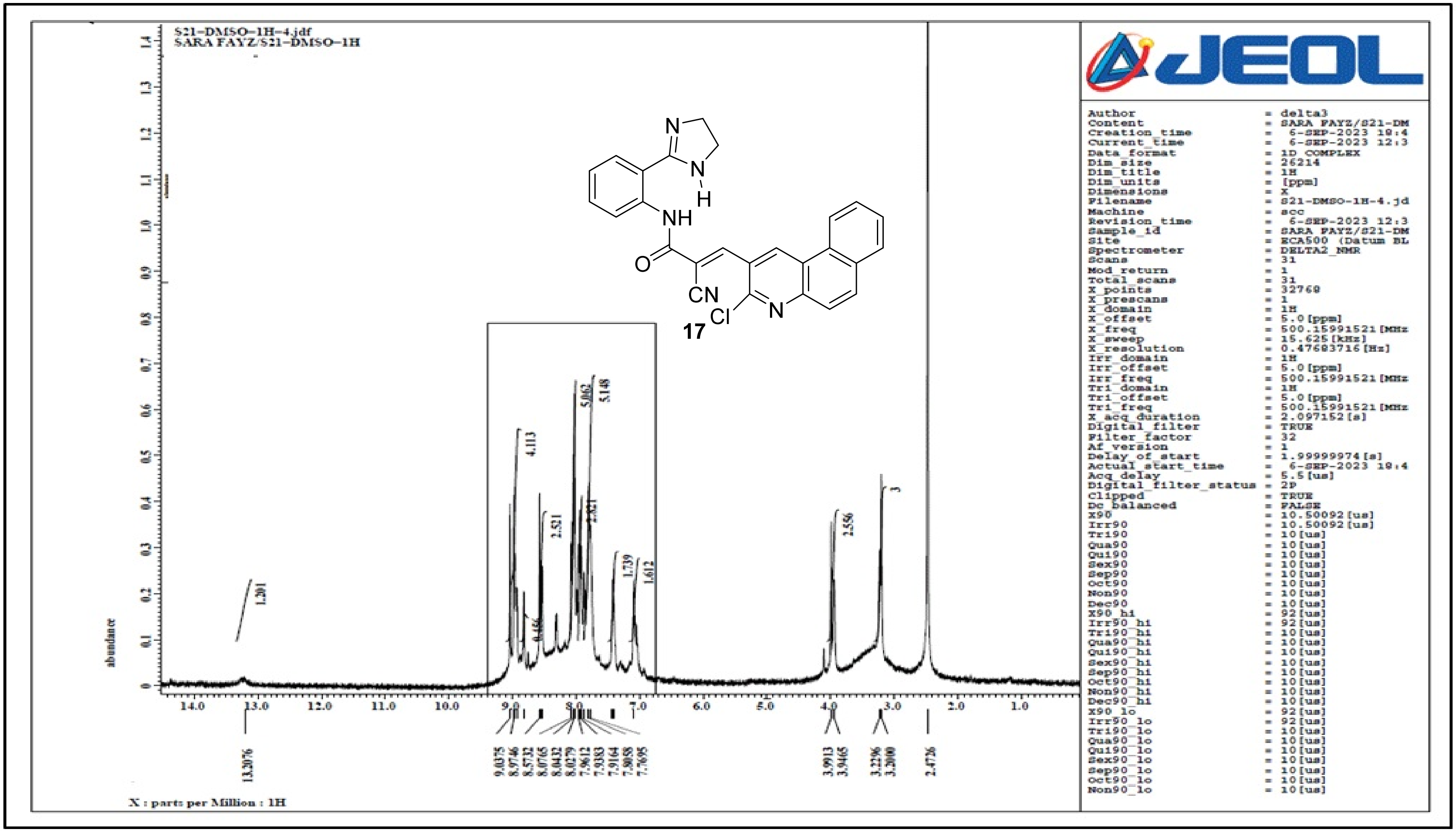

Fig. SD52. ^{1}H NMR spectrum (DMSO-d_6) of compound **17**

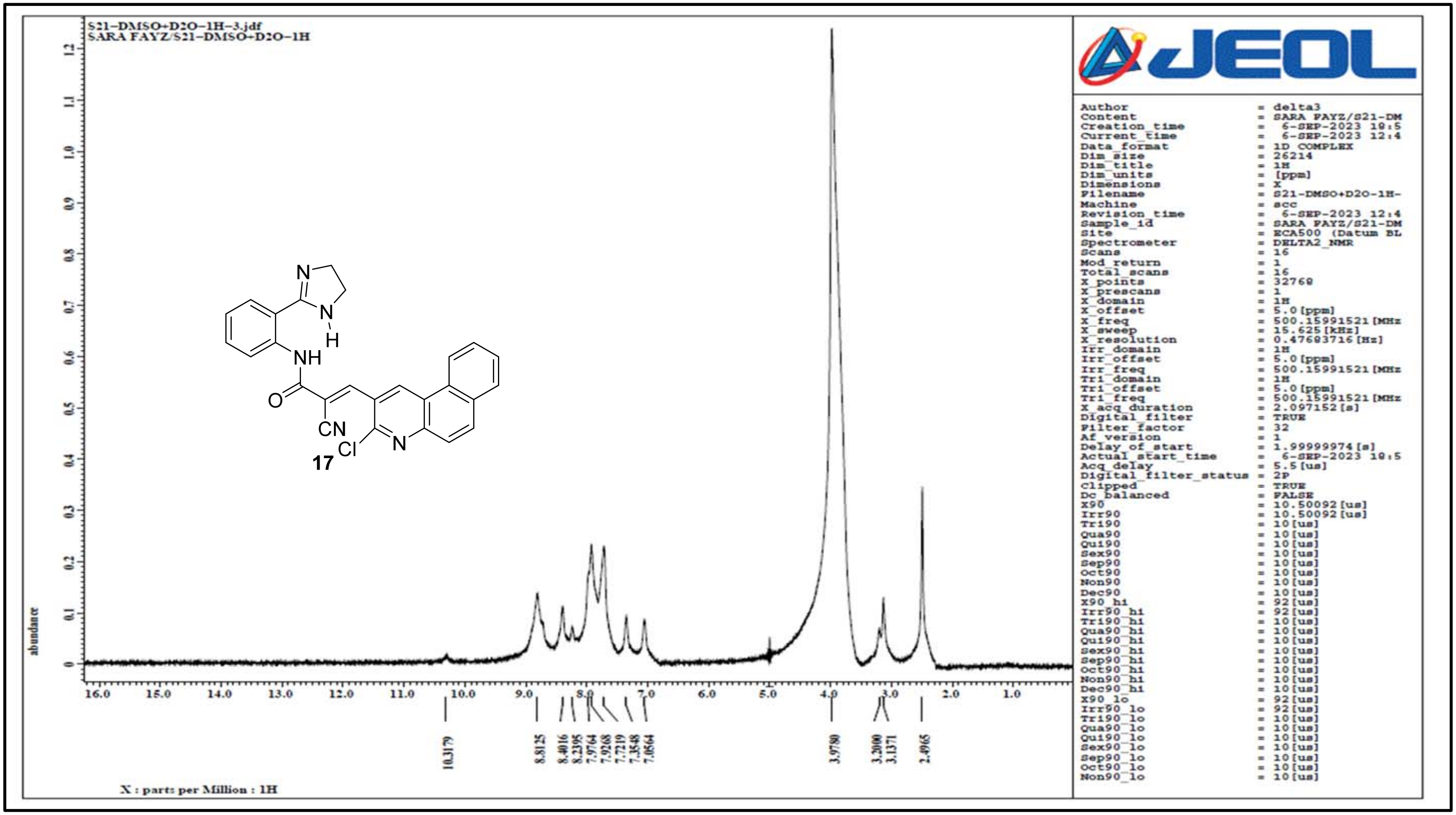

Fig. SD53. ^{1}H NMR spectrum (DMSO-d_6+D_2O) of compound **17**

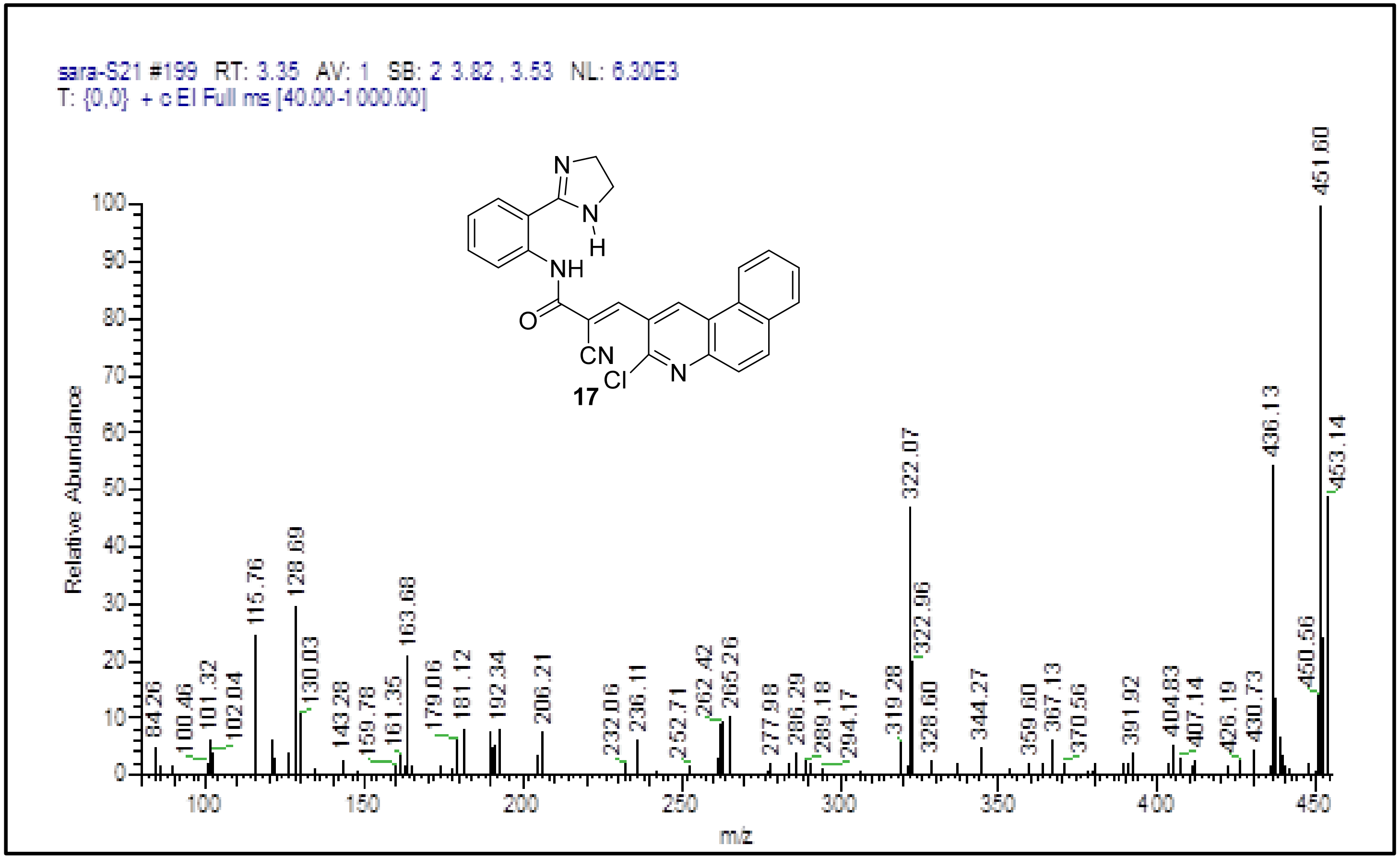

Fig. SD54. Mass spectrum of compound **17**

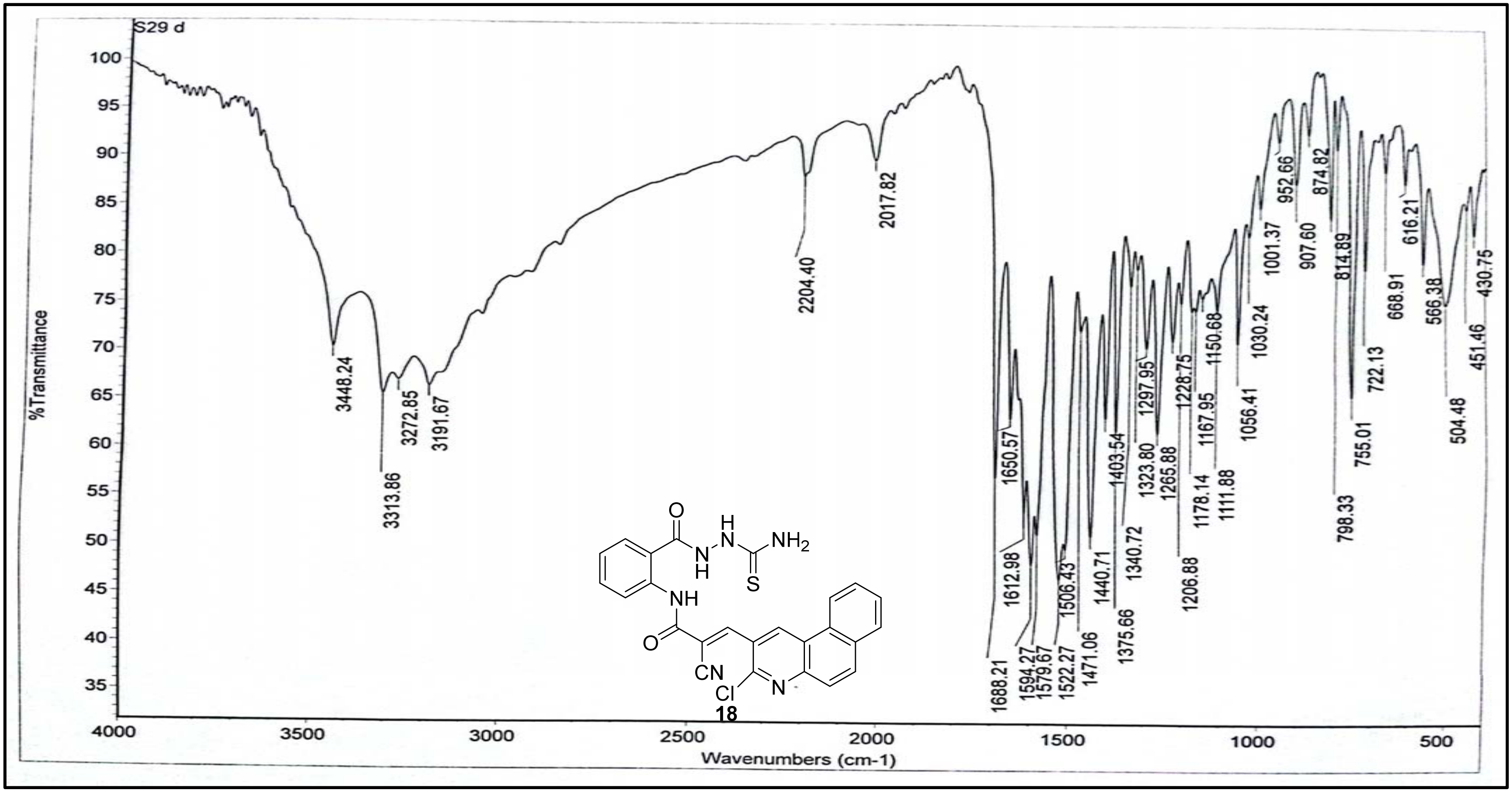

Fig. SD55. IR spectrum of compound **18**

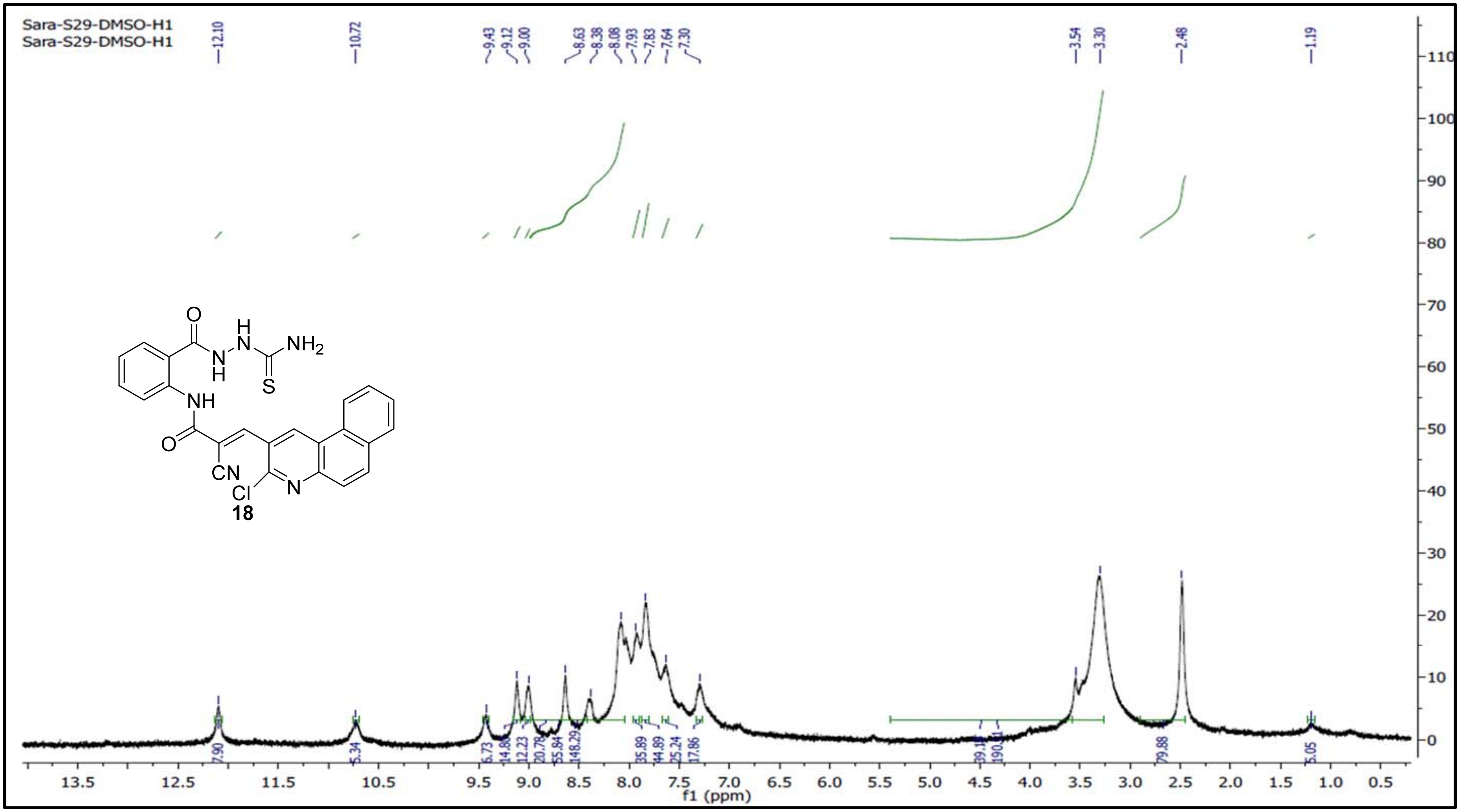

Fig. SD56. ^{1}H NMR spectrum (DMSO-d_6) of compound **18**

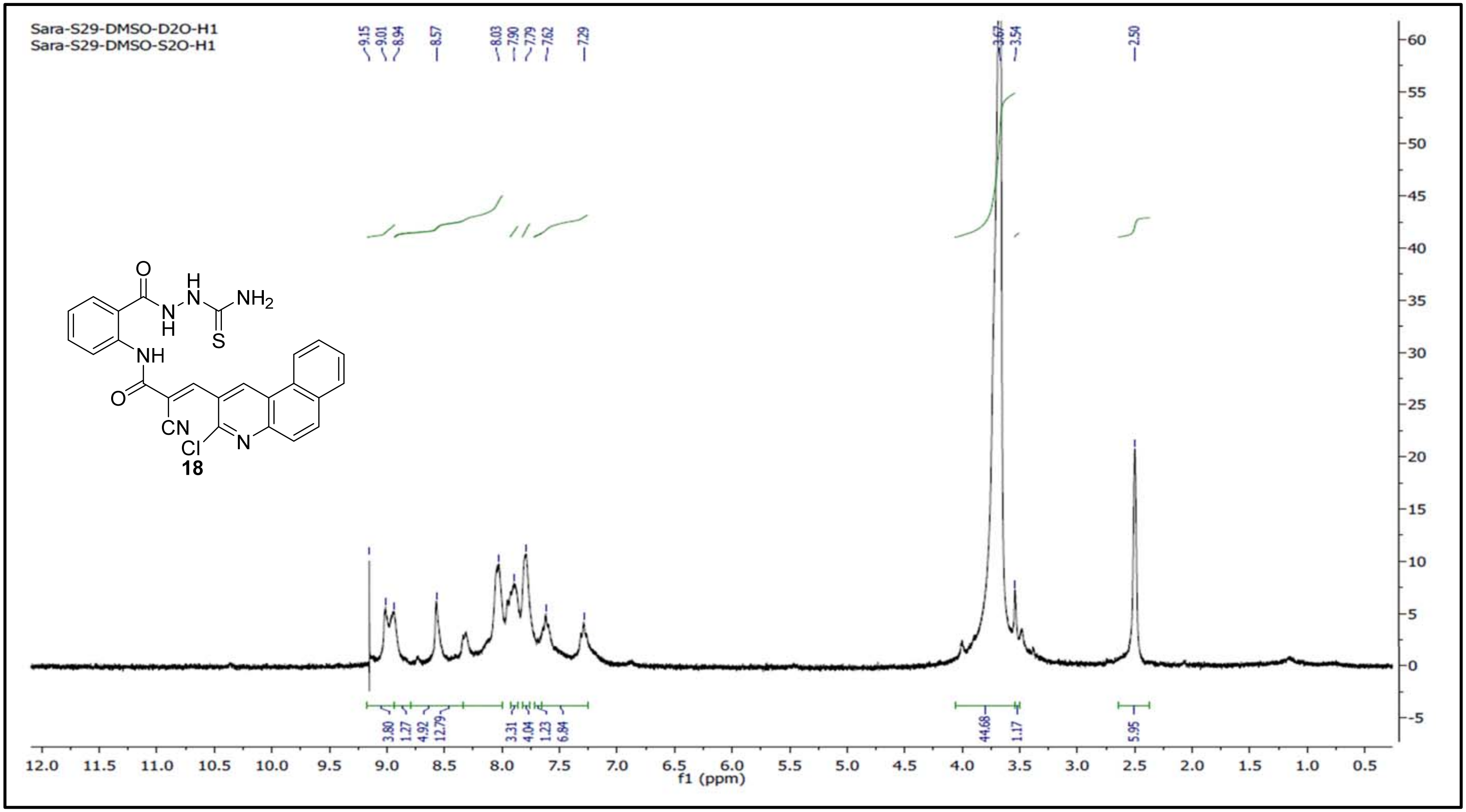

Fig. SD57. ^{1}H NMR spectrum (DMSO-d_6+D_2O) of compound **18**

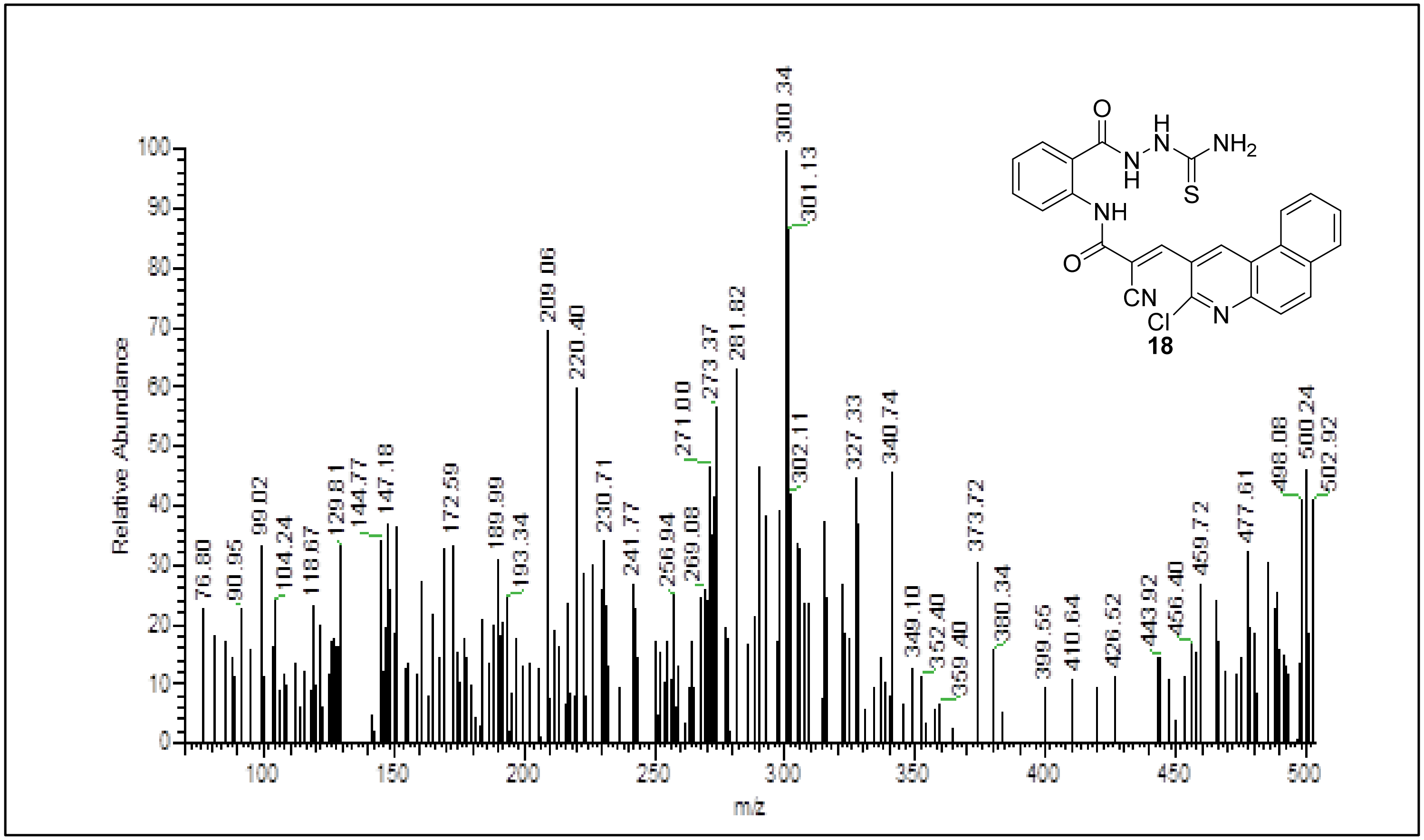

Fig. SD58. Mass spectrum of compound **18**

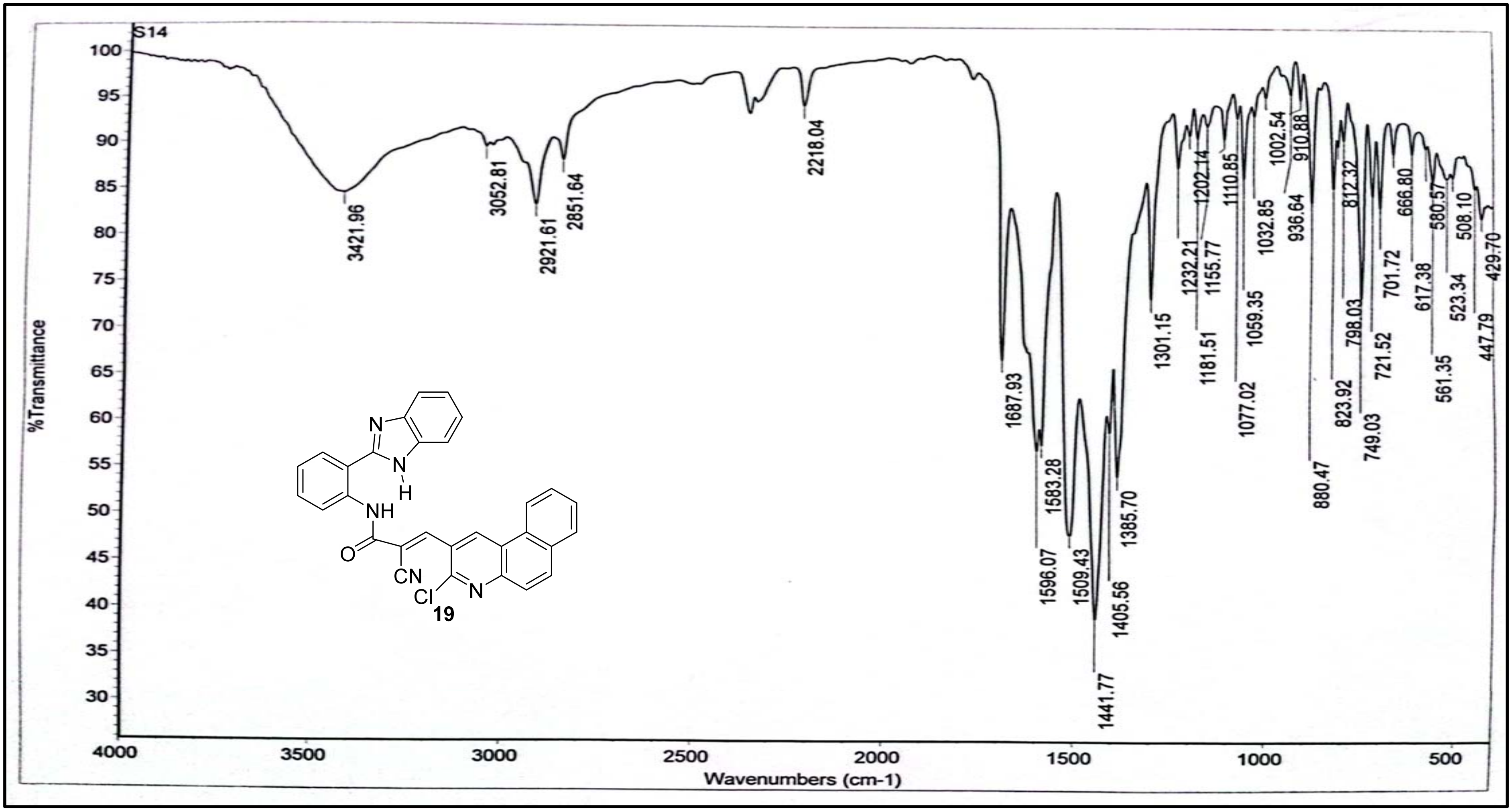

Fig. SD59. IR spectrum of compound **19**

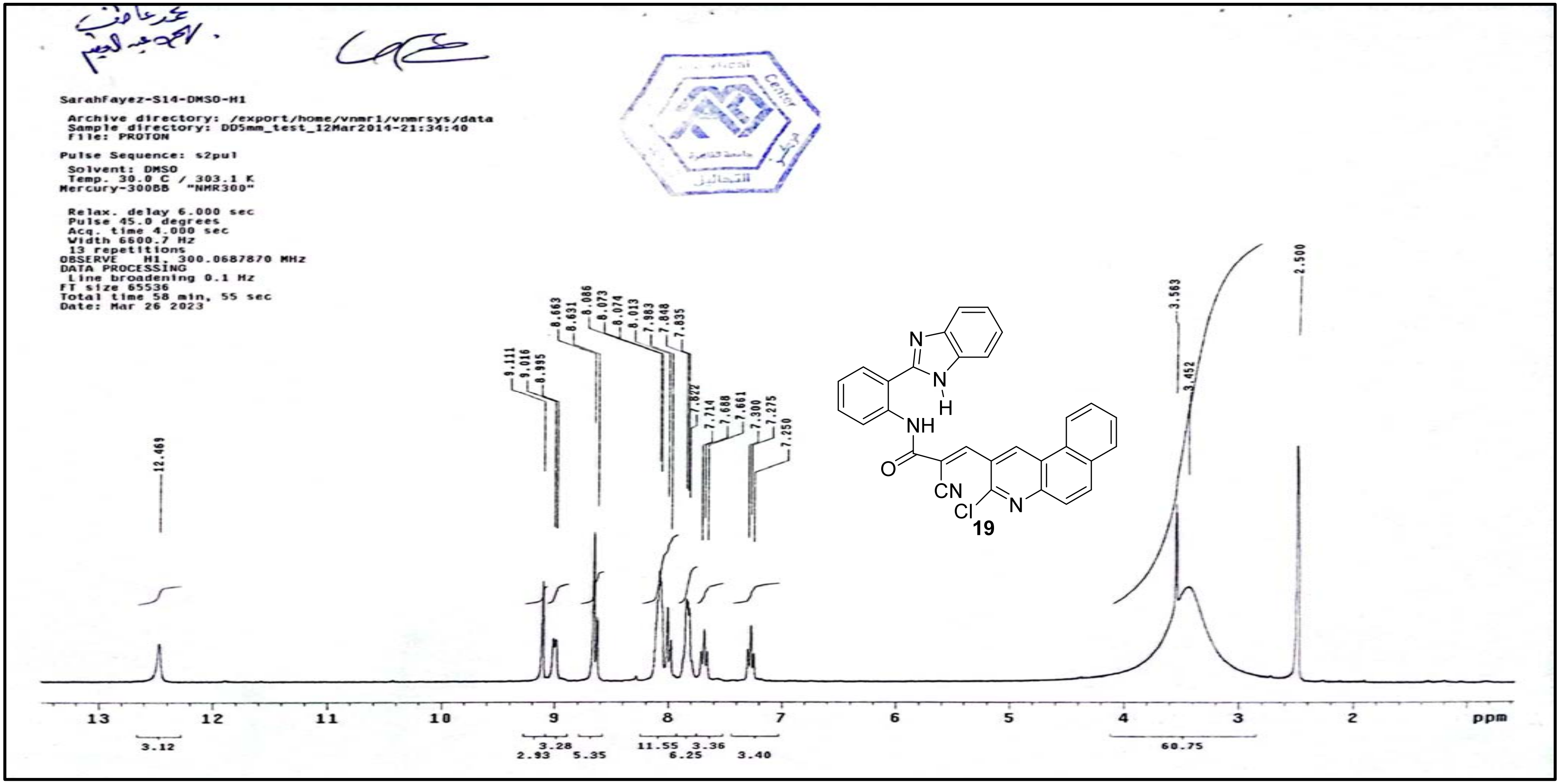

Fig. SD60. ^{1}H NMR spectrum (DMSO-d_6) of compound **19**

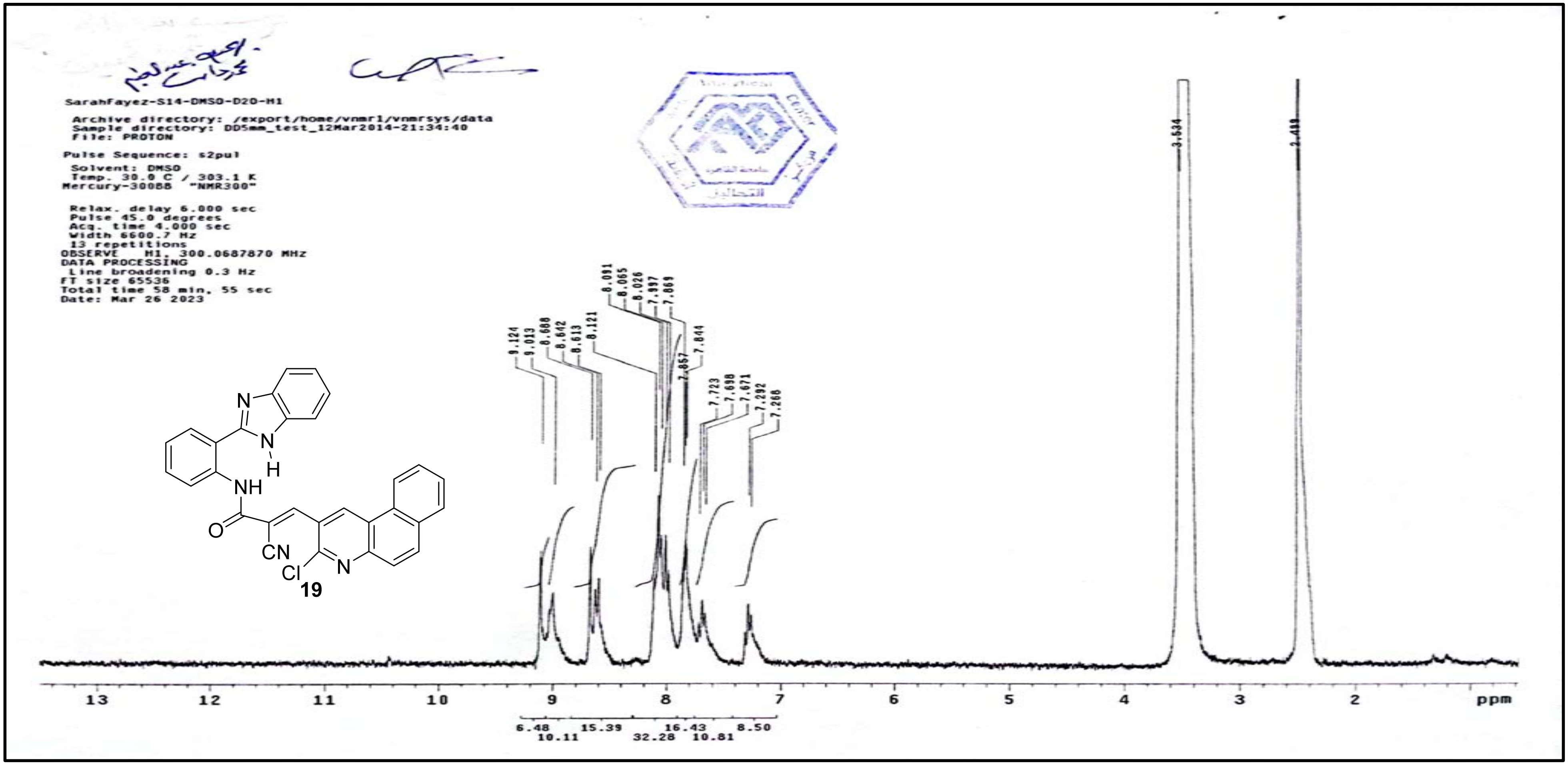

Fig. SD61. ^{1}H NMR spectrum (DMSO-d_6) of compound **19**

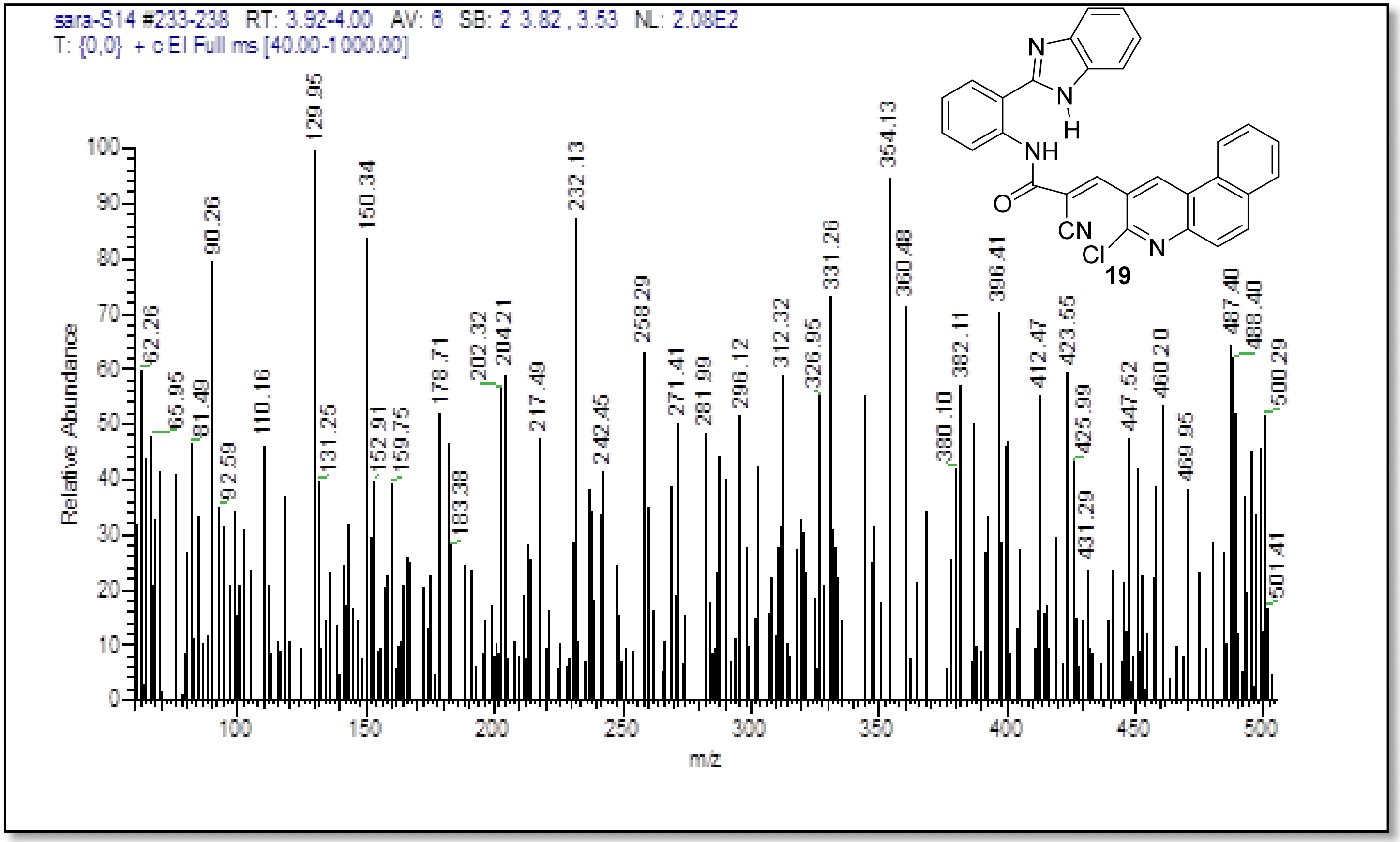

Fig. SD62. Mass spectrum of compound **19**
