# Supplementary material for: Antioxidant activity, molecular docking, and modeling pharmacokinetics study of some benzo[f]quinoline candidates
Source: Sci Rep. 2025 May 13;15:16522. doi: 10.1038/s41598-025-99811-1 (PMC12075698; doi:10.1038/s41598-025-99811-1)
Supplement: Supplementary file 2 — Supplementary Information 2. [file 41598_2025_99811_MOESM2_ESM.docx]

**Antioxidant Activity, Molecular Docking, and Modeling Pharmacokinetics Study of Some Benzo[*f*]quinoline Candidates**

**Sara F. El-Fagal ^1,^**^Ψ^**, Eman A. E. El-Helw ^1,^**^Ψ,^**^*^, Eman A. El-Bordany ^1^, Eman A. Ghareeb^1^**

^1^ Chemistry Department, Faculty of Science, Ain Shams University, Cairo, 11566, Egypt

****E-mail*:** [**eman.abdelrahman@sci.asu.edu.eg**](mailto:eman.abdelrahman@sci.asu.edu.eg)

Ψ: The first and second authors are equally contributed to this work.

**Supporting information:**

**Fig. S1**. A plausible pathway for the formation of imidazoline **17**.

**Fig. S2**. A plausible pathway for the formation of imidazoline **18**.

**Fig. S3**. A plausible pathway for the formation of benzimidazole **19**.

**Table S1.** 2D and 3D interactions of compound **19** with the active sites of HCV NS5B polymerase with respect to the co-crystallized ligand (053).

| **Compd.** | **2D** | **3D** |
| --- | --- | --- |
| **19** | 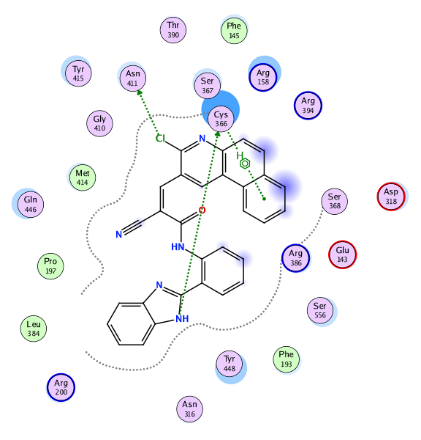 | 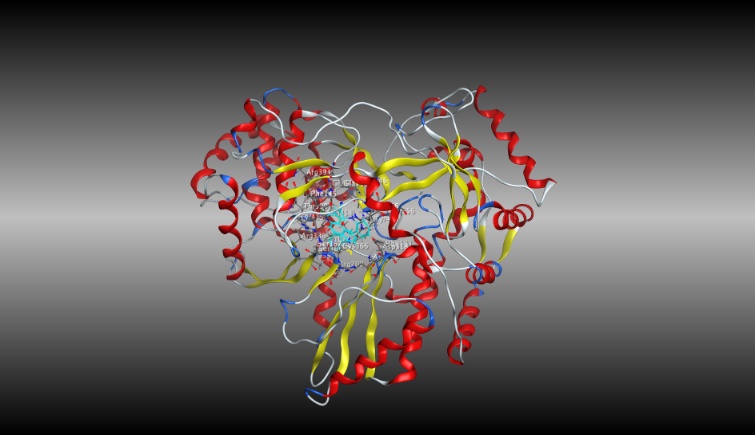 |
| **Co-crystallized ligand (053)** | 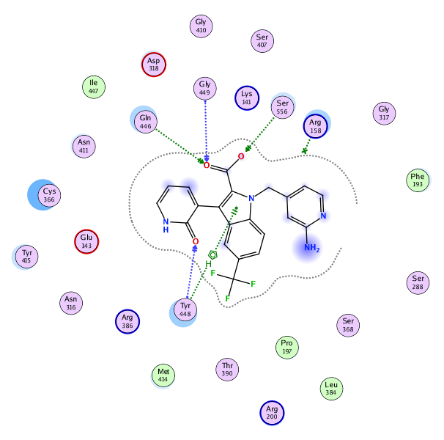 | 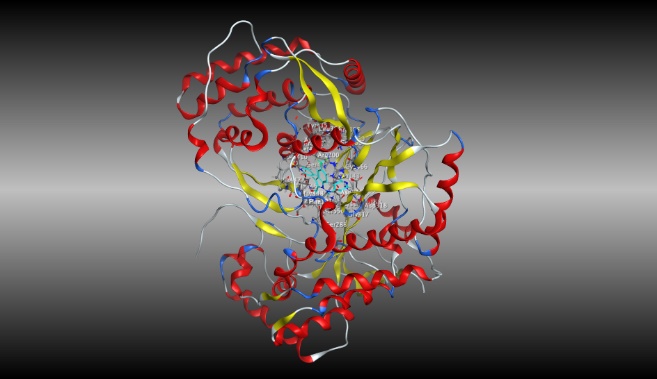 |

**Table S2**. DFT parameters of the potent compounds.

| **Compds**. | **Optimized structure** | **∆E** | **E^a^** | **Dipole/dipole** | **1,4-VDW^b^** | **Ip^c^** |
| --- | --- | --- | --- | --- | --- | --- |
| **3** |  | 1.351 | 29.970 | 10.274 | 22.494 | 7.512 |
| **9** |  | 2.108 | 38.285 | -5.215 | 38.698 | 7.497 |
| **13** |  | 1.558 | 30.074 | -14.953 | 36.208 | 7.507 |
| **17** |  | 1.711 | 37.774 | -6.191 | 29.578 | 7.504 |
| **19** |  | 1.587 | 37.041 | -8.674 | 33.076 | 7.507 |
| **Ascorbic acid** |  | 9.139 | 8.483 | 3.412 | 4.705 | 10.533 |

^a^E: Total energy (kcal/mol) ^b^VDW: van-der Waal interaction ^c^Ip: Ionization potential (eV)

**Table S3.** Physicochemical Properties / Lipophilicity / Drug-likeness properties of the potent compounds.

| **Entry** | | **Compounds** | | | | |
| --- | --- | --- | --- | --- | --- | --- |
|  |  | **3** | **9** | **13** | **17** | **19** |
| Molecular weight (g/mol) | | 308.72 | 464.95 | 544.99 | 451.91 | 499.95 |
| Num. heavy atoms | | 22 | 34 | 40 | 33 | 37 |
| Num. arom. heavy | | 14 | 24 | 26 | 20 | 29 |
| Fraction Csp3 | | 0.00 | 0.14 | 0.03 | 0.08 | 0.00 |
| Num. rotatable bonds | | 2 | 4 | 8 | 5 | 5 |
| Num. H-bond acceptor | | 4 | 4 | 5 | 4 | 4 |
| Num. H-bond donors | | 1 | 0 | 2 | 2 | 2 |
| Molar Refractivity | | 85.48 | 139.43 | 157.15 | 138.9 | 147.83 |
| TPSA (Å^2^) | | 73.98 | 71.57 | 111.95 | 90.17 | 94.46 |
| Consensus Log P_o/w_ | | 3.25 | 5.52 | 5.24 | 4.26 | 5.62 |
| Lipinskiˈs Rule | | **Yes** | **Yes** | **Yes** | **Yes** | **Yes** |
| Bioavailability Score | | **0.85** | **0.55** | **0.55** | **0.55** | **0.55** |
| **Pharmacokinetics** | | | | | | |
| GI absorption | **High** | | **High** | Low | **High** | Low |
| BBB permeant | Yes | | No | No | No | No |
| P-gp substrate | No | | No | No | No | No |
| CYP1A2 inhibitor | Yes | | No | No | Yes | No |
| CYP2C19 inhibitor | Yes | | Yes | Yes | Yes | Yes |
| CYP2C9 inhibitor | Yes | | Yes | Yes | Yes | No |
| CYP2D6 inhibitor | No | | No | No | No | No |
| CYP3A4 inhibitor | No | | No | Yes | Yes | No |
| Log K_p_ (Skin permeation) (cm/s) | -5.14 | | -4.38 | -4.90 | -5.44 | -4.12 |


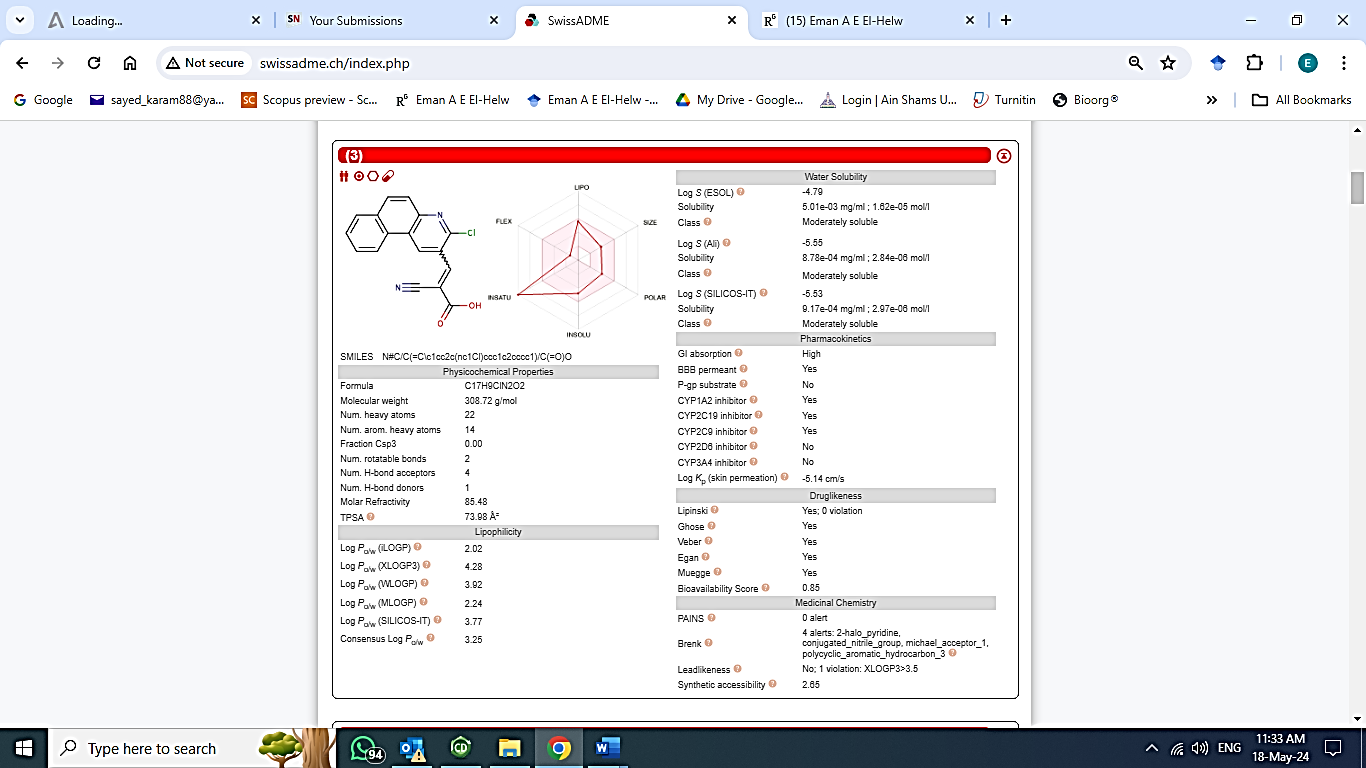


**Fig**. **S4**. ADME of compound **3**.


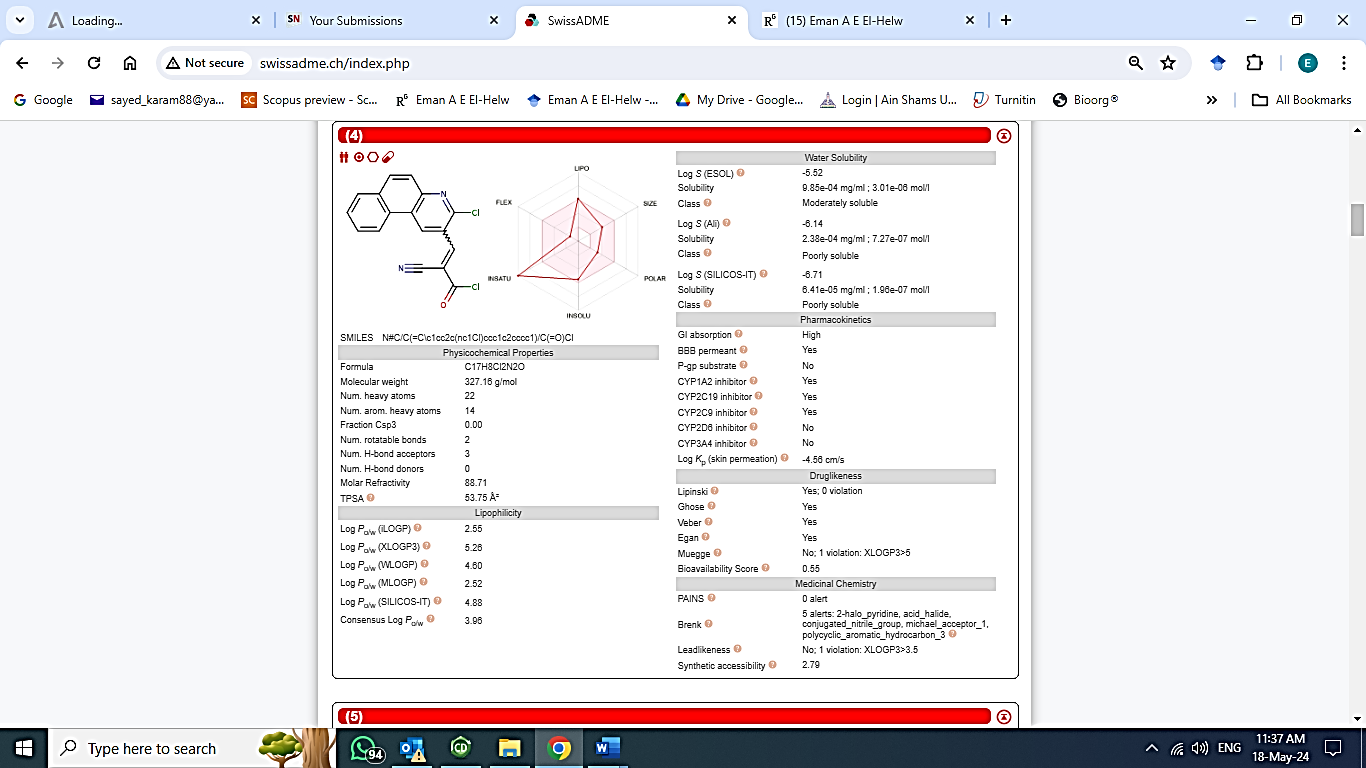


**Fig**. **S5**. ADME of compound **4**.


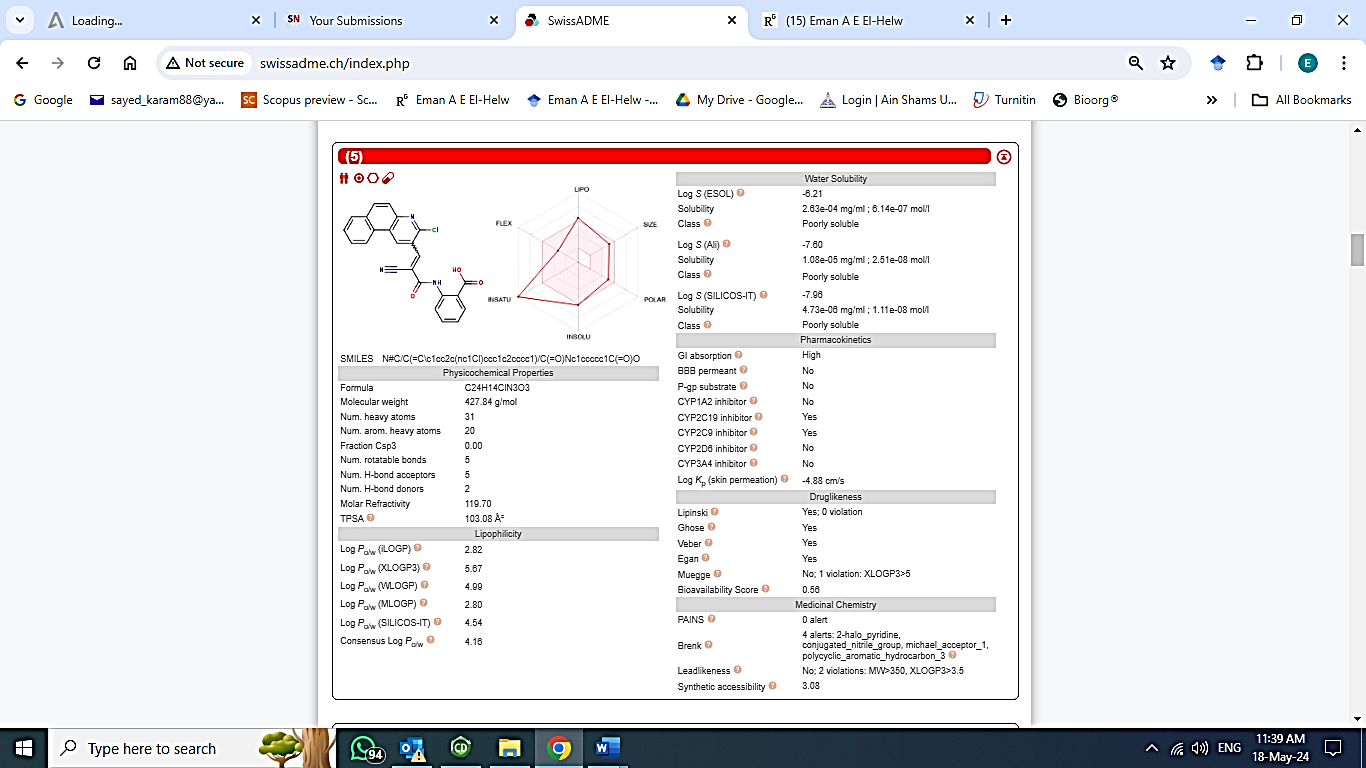


**Fig. S6.** ADME of compound **5**.


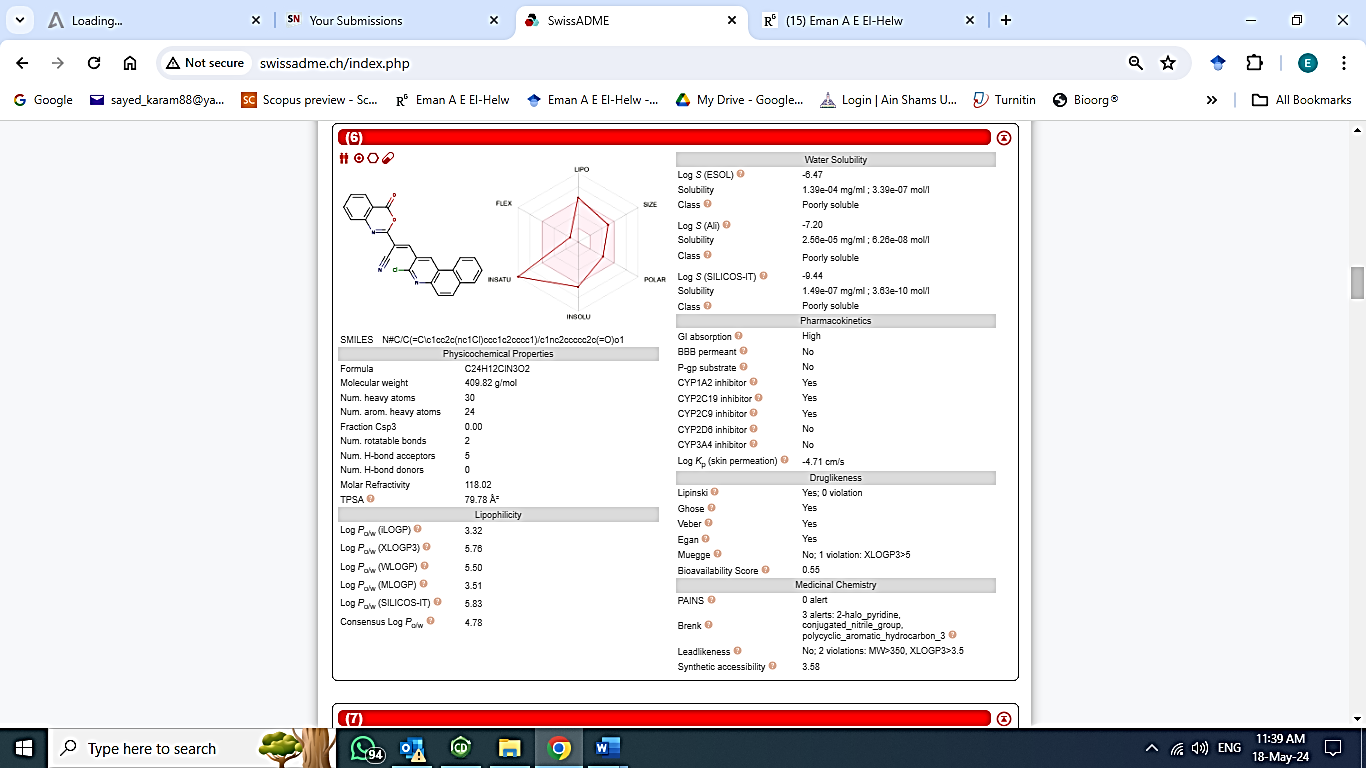


**Fig. S7.** ADME of compound **6**.


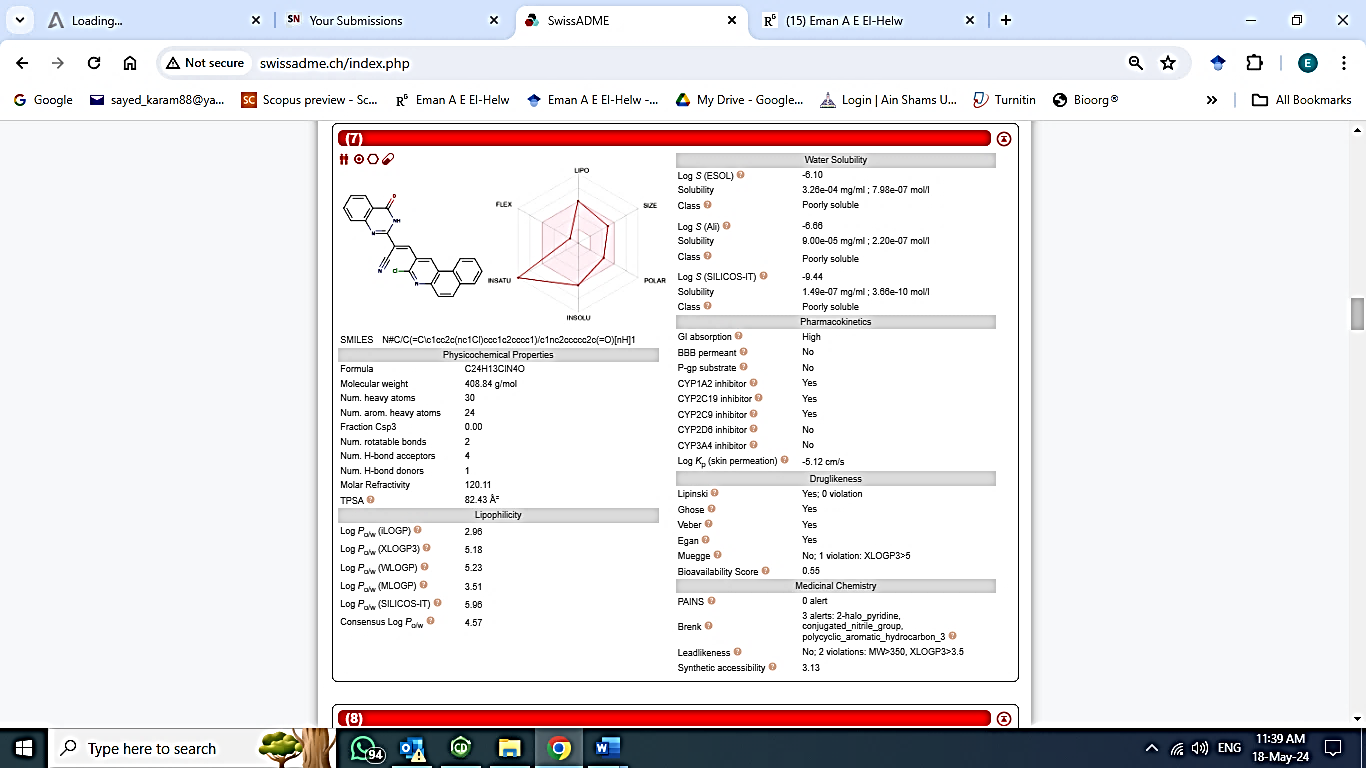


**Fig.** **S8**. ADME of compound **7**.


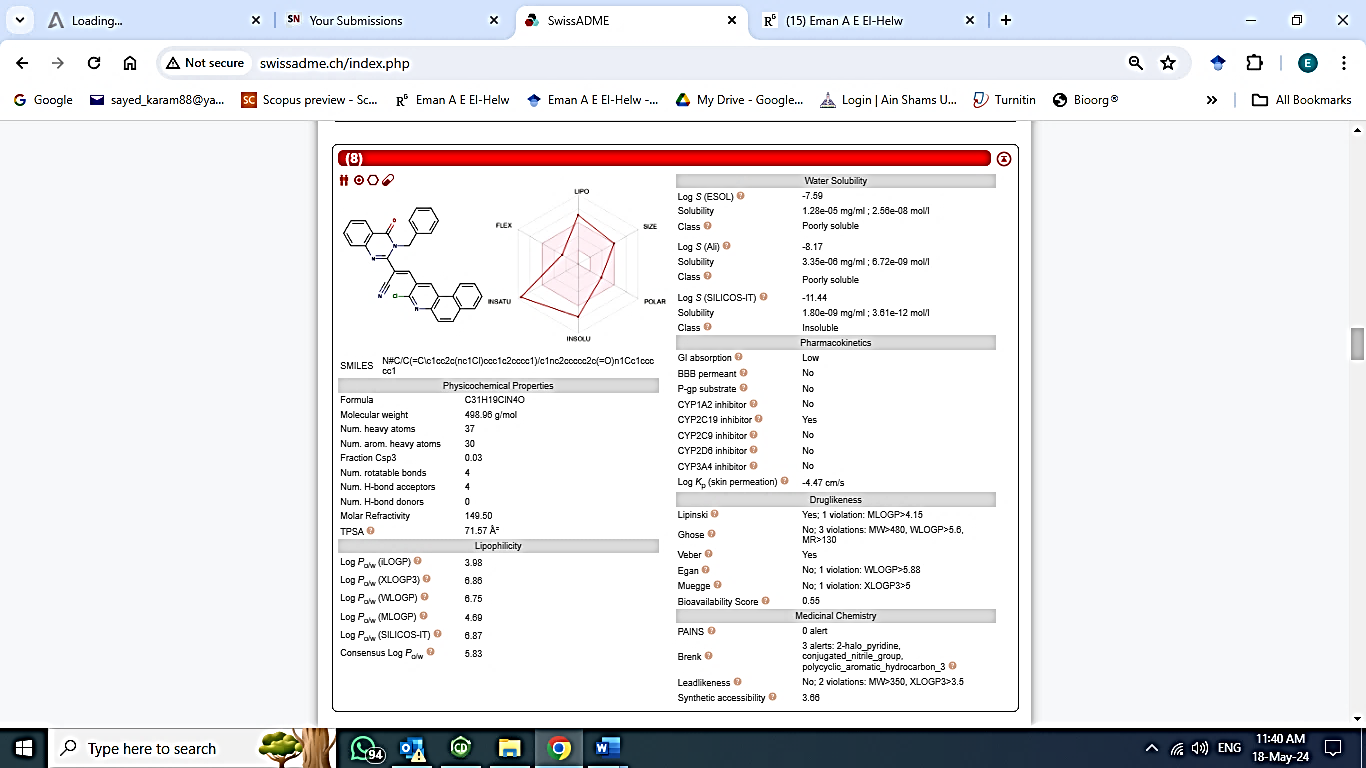


**Fig. S9.** ADME of compound **8**.


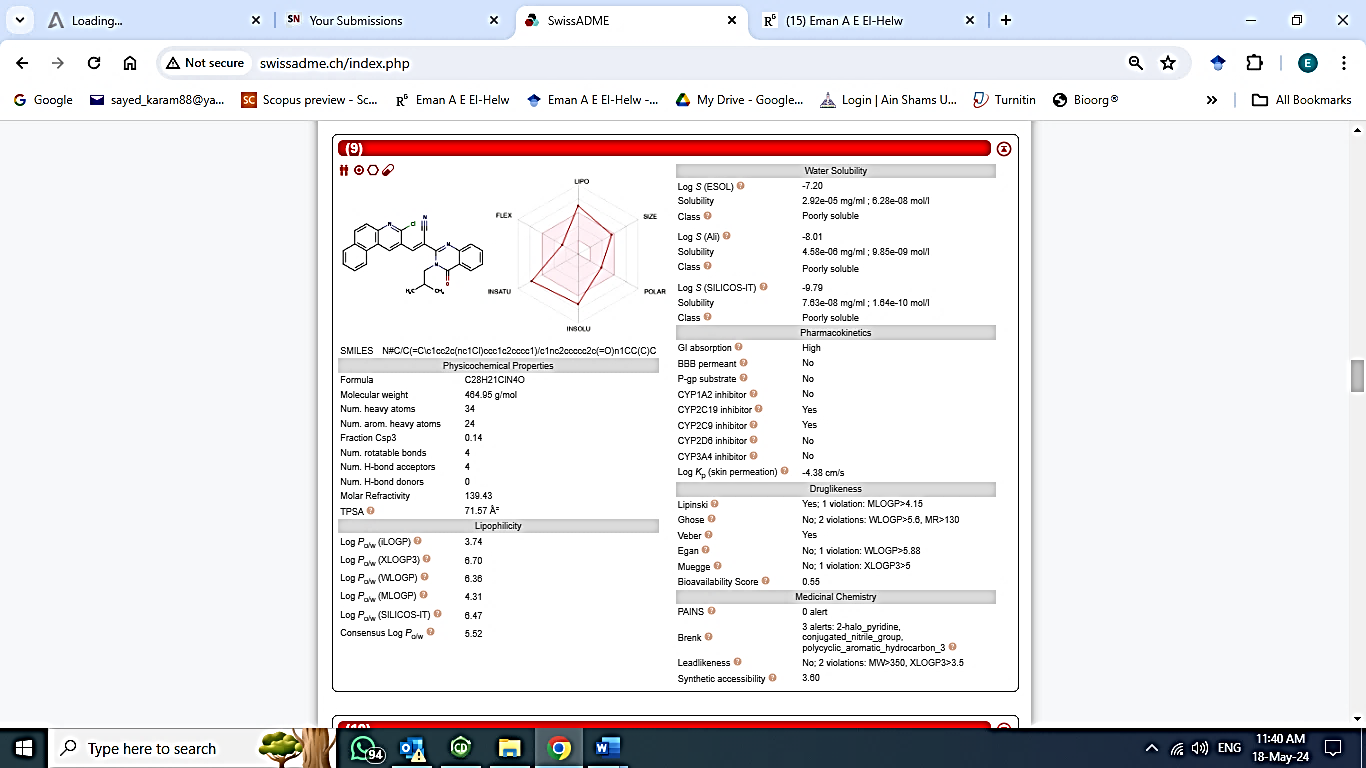


**Fig. S10**. ADME of compound **9**.


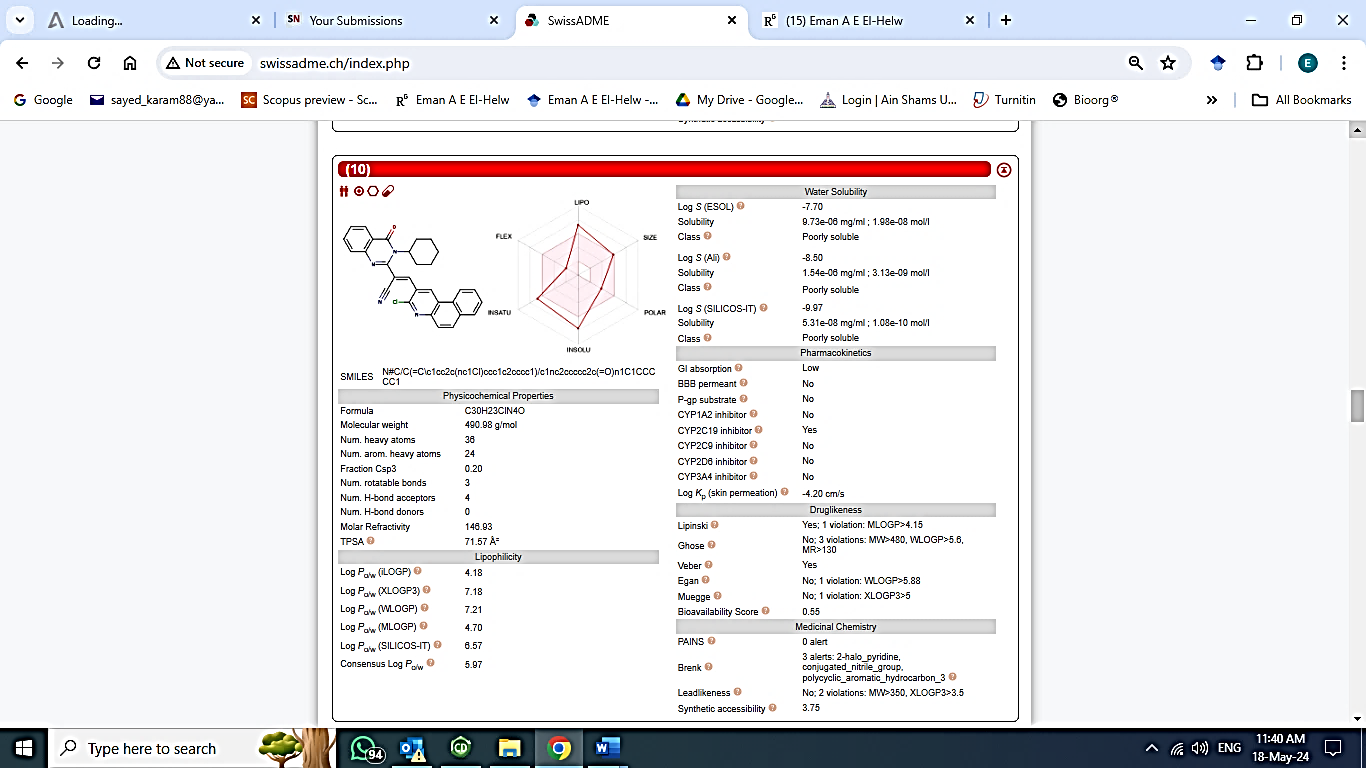


**Fig. S11**. ADME of compound **10**.


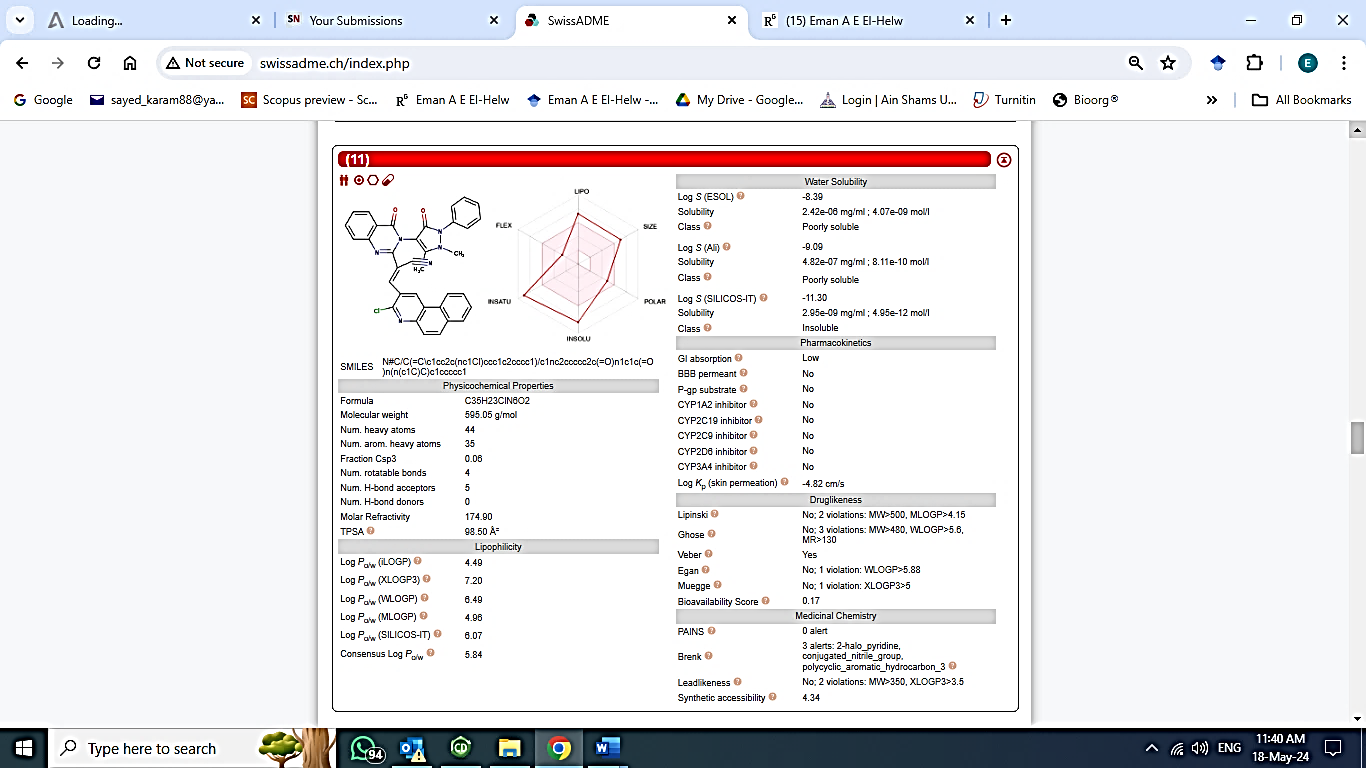


**Fig. S12**. ADME of compound **11**.


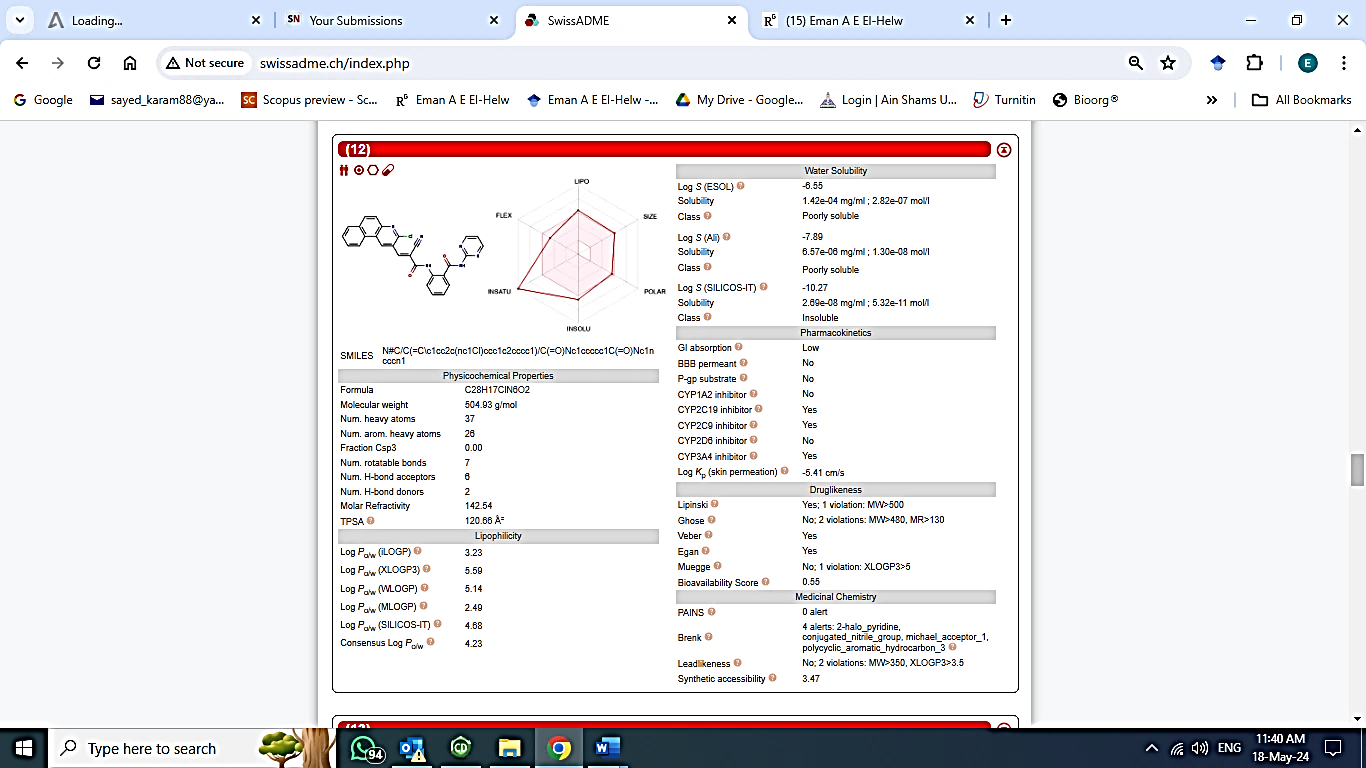


**Fig. S13**. ADME of compound **12**.


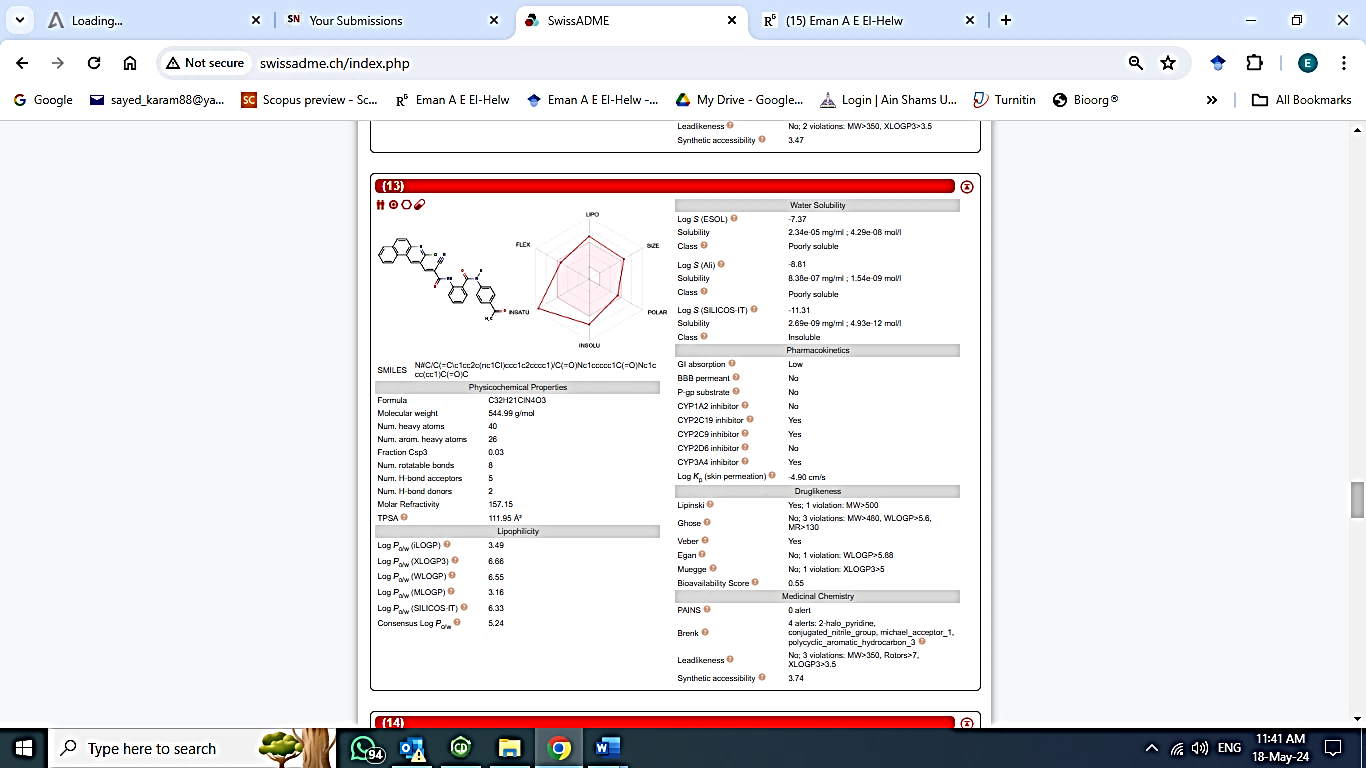


**Fig**. **S14**. ADME of compound **13**.


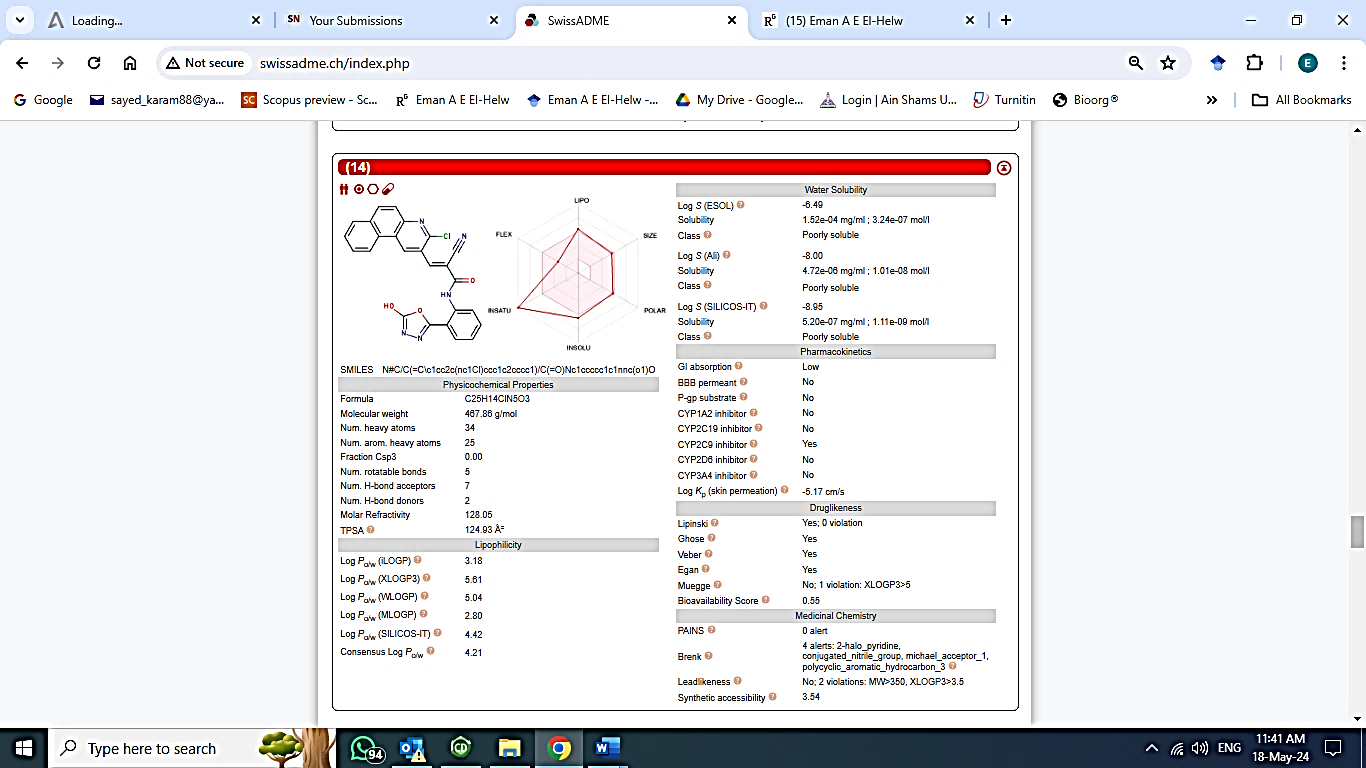


**Fig**. **S15**. ADME of compound **14**.


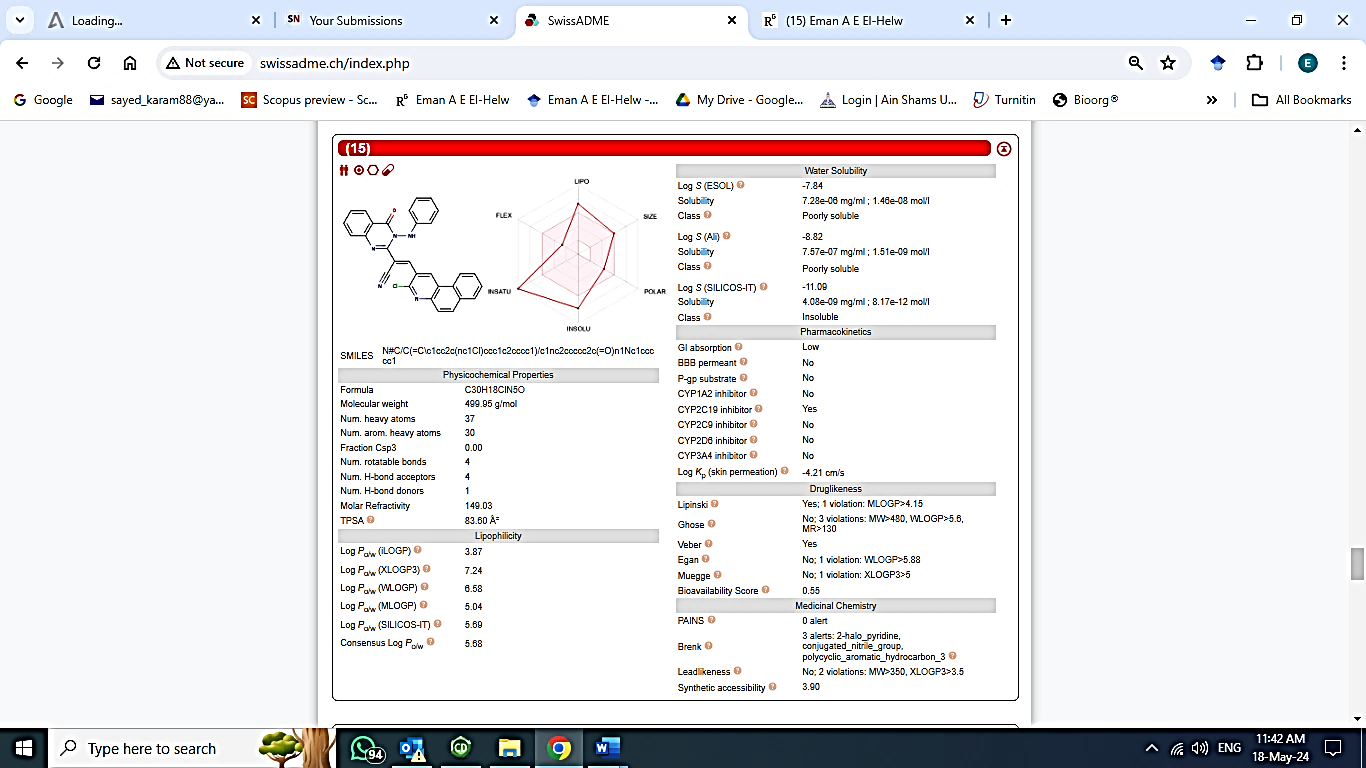


**Fig**. **S16**. ADME of compound **15**.


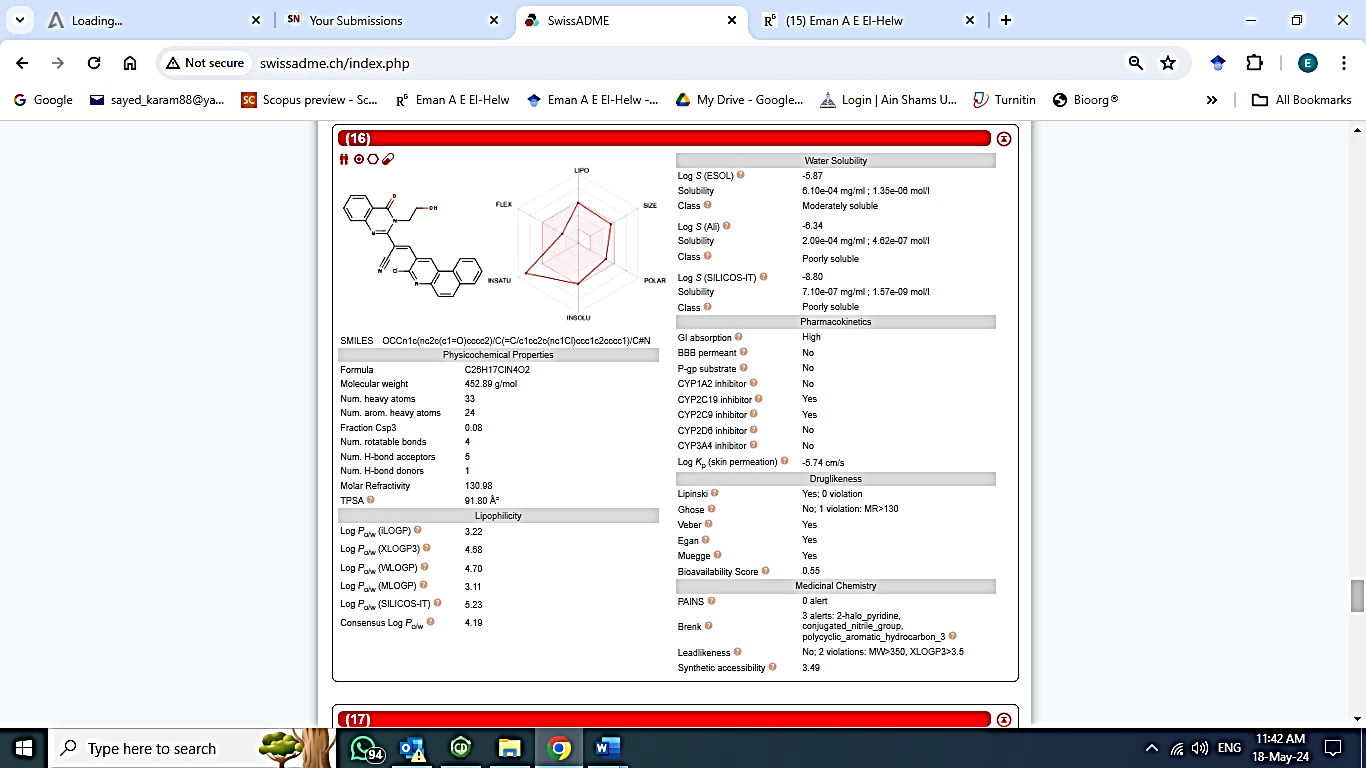


**Fig**. **S17**. ADME of compound **16**.


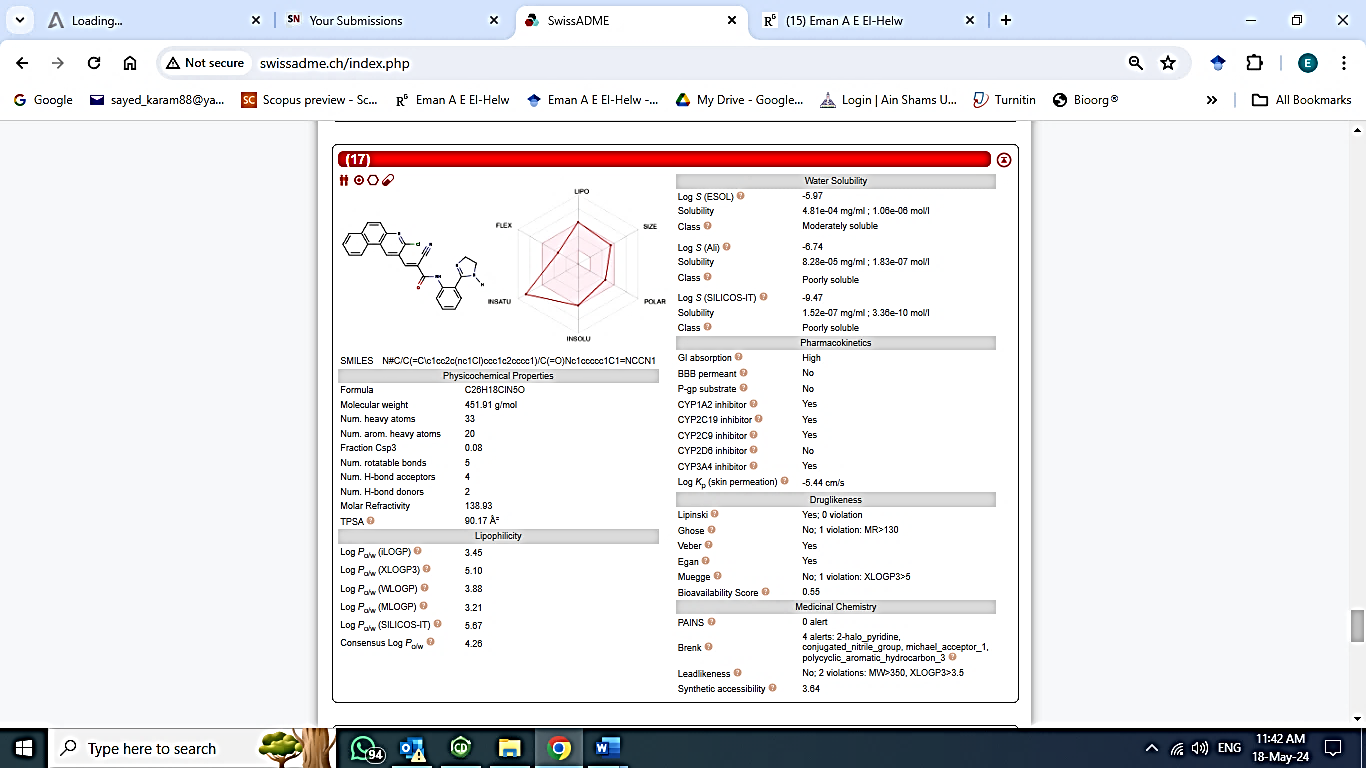


**Fig**. **S18**. ADME of compound **17**.


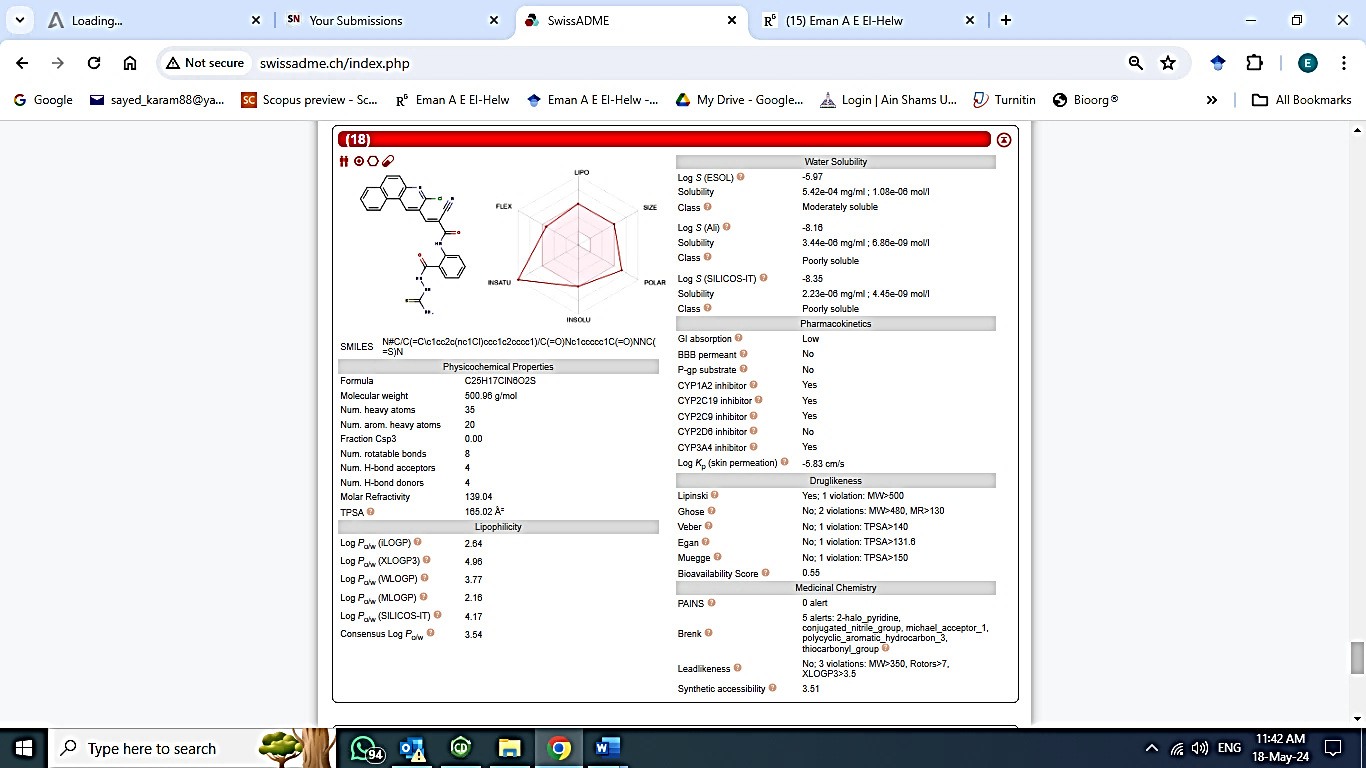


**Fig**. **S19**. ADME of compound **18**.


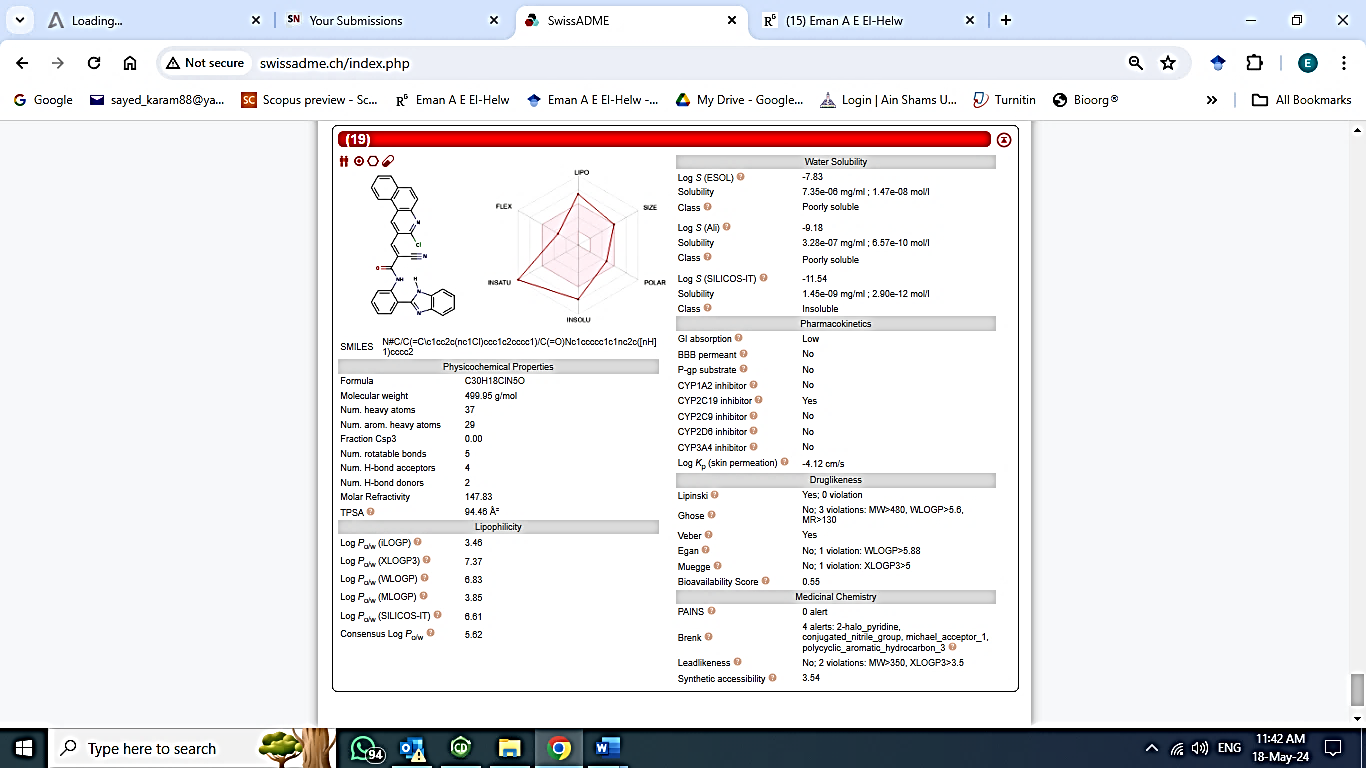


**Fig**. **S20**. ADME of compound **19**.
